# Synthesis of α-Aminophosphonic Acid Derivatives Through the Addition of *O*- and *S*-Nucleophiles to 2*H*-Azirines and Their Antiproliferative Effect on A549 Human Lung Adenocarcinoma Cells

**DOI:** 10.3390/molecules25153332

**Published:** 2020-07-22

**Authors:** Victor Carramiñana, Ana M. Ochoa de Retana, Francisco Palacios, Jesús M. de los Santos

**Affiliations:** Department of Organic Chemistry I, Faculty of Pharmacy and Lascaray Research Center, University of the Basque Country (UPV/EHU), 01006 Vitoria, Spain; victor.carraminana@ehu.eus (V.C.); anamaria.ochoaderetana@ehu.eus (A.M.O.d.R.)

**Keywords:** allylic α-aminophosphorus compounds, α-aminophosphine oxide or phosphonate acetals, antiproliferative effect, aziridines, 2*H*-azirines

## Abstract

This work reports a straightforward regioselective synthetic methodology to prepare α-aminophosphine oxides and phosphonates through the addition of oxygen and sulfur nucleophiles to the C–N double bond of 2*H*-azirine derivatives. Determined by the nature of the nucleophile, different α-aminophosphorus compounds may be obtained. For instance, aliphatic alcohols such as methanol or ethanol afford α-aminophosphine oxide and phosphonate acetals after N–C3 ring opening of the intermediate aziridine. However, addition of 2,2,2-trifluoroethanol, phenols, substituted benzenthiols or ethanethiol to 2*H*-azirine phosphine oxides or phosphonates yields allylic α-aminophosphine oxides and phosphonates in good to high general yields. In some cases, the intermediate aziridine attained by the nucleophilic addition of *O*- or *S*-nucleophiles to the starting 2*H*-azirine may be isolated and characterized before ring opening. Additionally, the cytotoxic effect on cell lines derived from human lung adenocarcinoma (A549) and non-malignant cells (MCR-5) was also screened. Some α-aminophosphorus derivatives exhibited very good activity against the A549 cell line in vitro. Furthermore, selectivity towards cancer cell (A549) over non-malignant cells (MCR-5) has been detected in almost all compounds tested.

## 1. Introduction

α-Aminophosphonic acids are structural bioisosteres of amino acids displaying a wide range of biological properties and applications in many areas ranging from agrochemistry to medicine [1,2,3,4]. Some of their varied applications include antitumor agents [5,6,7], potent antibiotics [8,9], as antibacterial agents [10,11], antiviral [12], and enzyme inhibitors [13,14,15] such as renin [14,16], or HIV protease [17,18], among others. Joined with their structural similarity to natural carboxylic acids, the intriguing properties of α-aminophosphonic acids also stem from the fact that the tetrahedral geometry of phosphonic acid functionality resembles in a stable manner the high-energy transition state of peptide bond hydrolysis [19]. The last-mentioned feature is directly responsible for the biological activity of α-amino-phosphonic acids, mostly as enzyme inhibitors involved in peptide metabolism.

**Figure 1:**
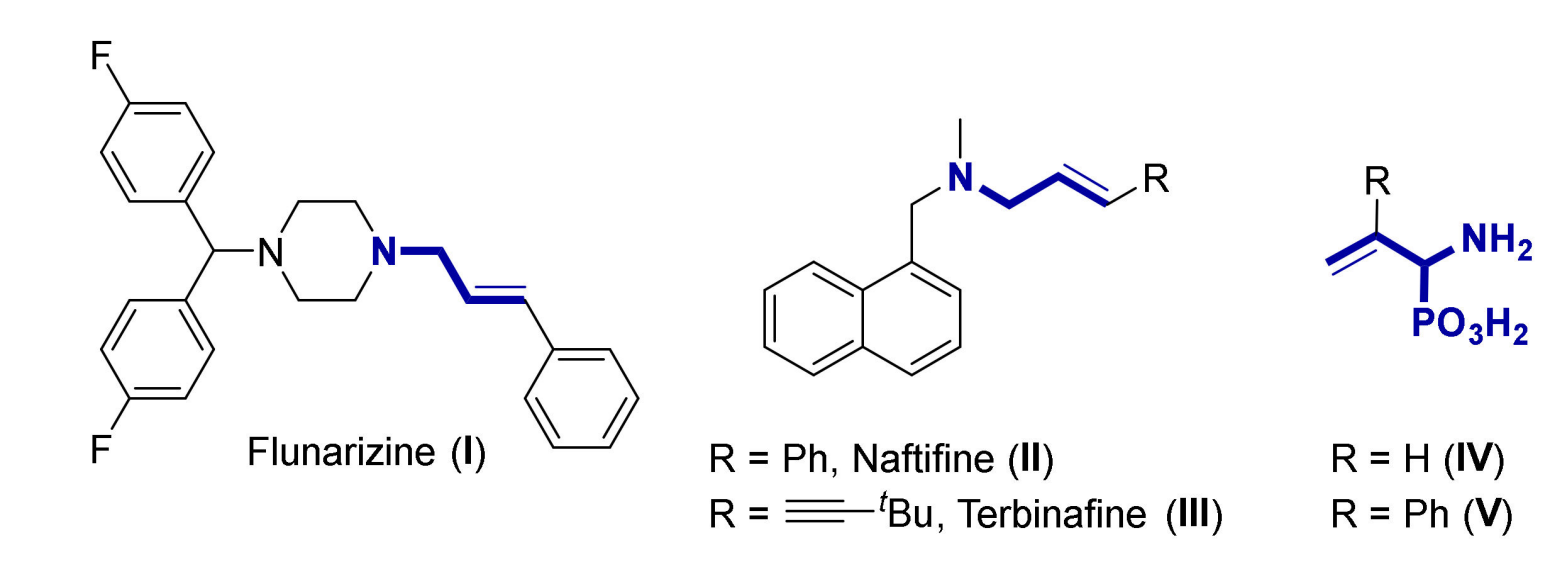
Representative examples of drugs containing allylic amine or allylic α-aminophosphonic acid functionalities.

Moreover, it is well-known that allylic amines [20,21,22] are key structural features in variety of natural products and pharmaceuticals, such as the calcium channel blocker flunarizine (**I**) [23], effective in the prophylaxis of migraine, and the antifungal drugs naftifine (**II**) [24,25] and terbinafine (**III**) [24,26], and have been recognized as an important class of organic compounds owing to their use as valuable intermediates vital for molecular complexity buildup [27] (Figure 1).

To overcome the drastic side effects related to a single drug, hybrid molecules modulate several targets of multifactorial diseases, and have been established as a popular approach for multidrug therapy [28,29]. Within this class of drugs, hybrids molecules introducing two potentially pharmacophores, including allylic amine moieties and α-aminophosphonic acid functional groups, such as allylic α-aminophosphonic acid derivatives (**IV** and **V**), have attracted scarce attention since only a few examples have been reported in the literature. For instance, (1-amino-2-propenyl)phosphonic acid (**IV**) inhibit alanine racemase and D-alanine:D-alanine ligase [30,31], while α-aminophosphonic acid analogue (**V**) of the natural phenylalanine bearing a methylidene at the β-position acts as an inhibitor of phenylalanine ammonia-lyases (PAL) [32] (Figure 1).

There is a new renewal of the interest in covalent binding therapeutics due to the FDA support of efficient and innocuous covalent drugs and a better understanding of the benefits of the covalent binding mechanism [33,34]. Numerous new drugs contain electrophilic moieties acting as “warheads”, and many molecules with a variety of electrophilic warheads, including epoxide, ketone, nitrile, ester, α,β-unsaturated carbonyl, or aziridine functionalities have been recognized as covalent inhibitors [35]. Aziridines as powerful alkylating agents, may act as covalent drugs by means of their ability to act as DNA cross-linking agents througH-Nucleophilic ring opening of the three-membered nitrogen heterocycle [36].

In this sense, we have been previously involved in the synthesis of phosphorus-substituted aziridines via nucleophilic addition to 2*H*-azirines [37,38,39,40] Moreover, these aziridines are valuable building blocks for the preparation of more complex products, such as 1*H*-benzo[*d*]azepines [37], pyrroles [37,41], oxazoles [42], and α- or β-aminophosphorus acid derivatives [43,44], among others. More recently, we have disclosed a diastereoselective approach to cyanoaziridines [45] and hybrid molecules, such as azirino [2,1-*b*]benzo[*e*][1,3]oxazines [46], througH-Nucleophilic addition of cyanide anion or functionalized phenols, respectively, to the C–N double bond of phosphorus substituted 2*H*-azirines. We were intrigued about the possibility of accessing other saturated aziridines containing phosphorus substituents by means of the addition of oxygen and sulfur nucleophilic reagents to 2*H*-azirines. For this aim, here we wish to account the results of the incorporation of aliphatic alcohols, phenols, thiols and benzenethiols into the three-membered nitrogen heterocycle, since these nucleophilic additions to 2*H*-azirines may be a new approach for the construction of substituted aziridines containing phosphorus substituents or even, more complex ring opening compounds. Furthermore, all these new functionalized acyclic and heterocyclic compounds were proved for antiproliferative activity against human cancer cell lines. This strategy entails a stimulating challenge due to the inherent interest of these new molecules, both in synthetic and medicinal chemistry.

## 2. Results

### 2.1. Chemistry

**Scheme 1:**
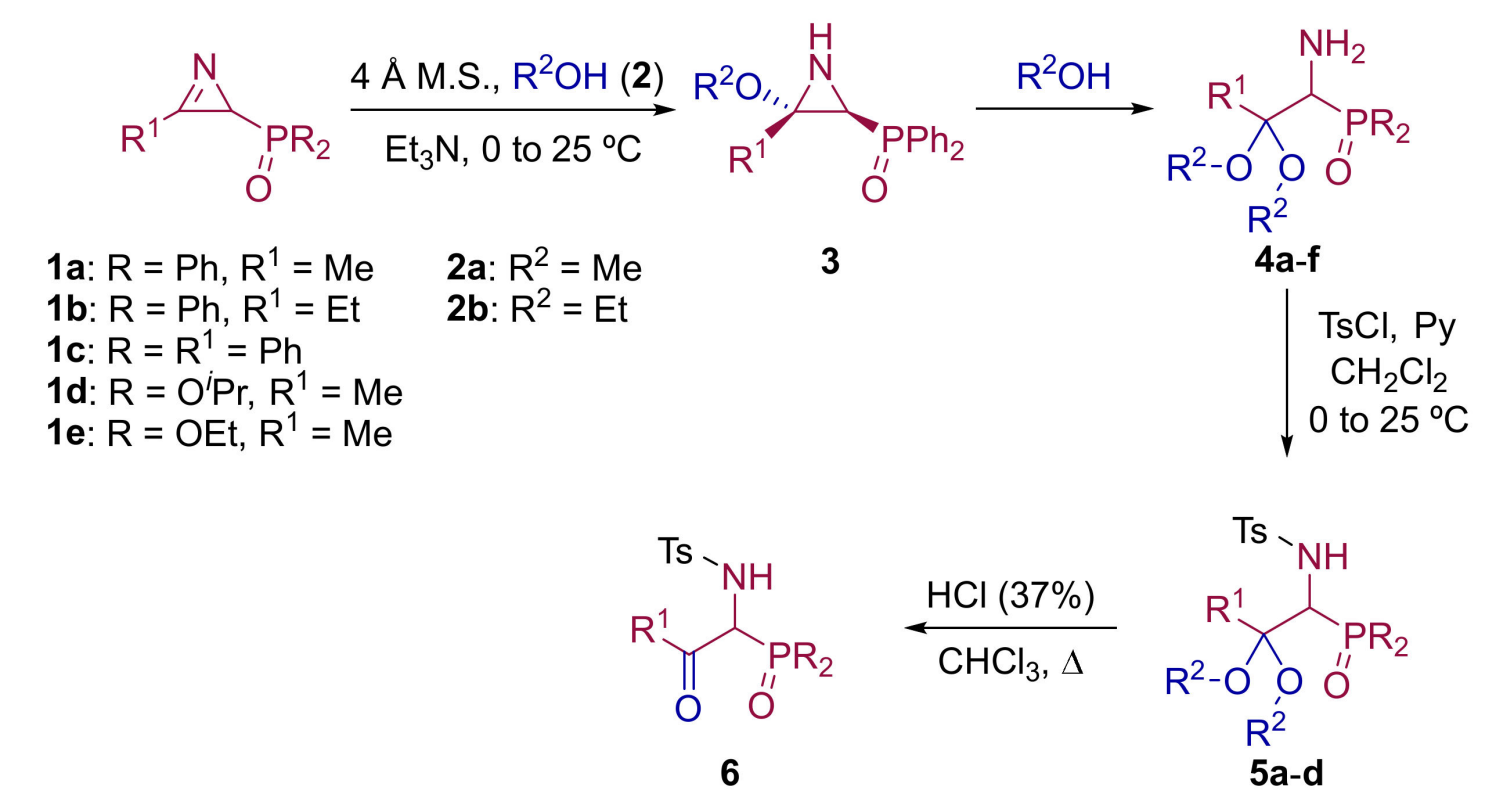
Synthesis of α-aminophosphine oxides and phosphonates **4**–**6**. All synthesized compounds are racemic.

First, we studied the addition of aliphatic alcohols **2a**–**b** to 2*H*-azirine phosphine oxides and phosphonates **1**. Initially, we tested the reaction of 2*H*-azirine phosphine oxide **1a** with two equivalents of methanol (**2a**) in the presence of triethylamine as the base and using methylene chloride as the solvent. Since no reaction was observed using these conditions, the reaction of 2*H*-azirines **1** with aliphatic alcohol **2** as nucleophilic reagent and as the solvent, all at once, was assessed. Therefore, as outlined in Scheme 1, in an initial experiment the nucleophilic addition of methanol (**2a**) to 2*H*-azirine-phosphine oxide **1a** (R = Ph, R^1^ = Me) was readily achieved using Et_3_N at 25 °C and MeOH as the nucleophile and solvent. Under these reaction conditions, we anticipated to obtain the desired aziridine **3a**, as previously observed in the reaction of fluoroalkylated 2*H*-azirines with methanol [38]. Conversely, rather than aziridine **3a**, functionalized α-aminophosphine oxide dimethyl acetal (**4a**, R = Ph, R^1^ = R^2^ = Me, Table 1, entry 1) in 74% yield was attained, as evidenced by the two sets of signals for the methoxy group which appeared as singlets in ^1^H-NMR (see the Supplementary Data). Starting from 2*H*-azirine-phosphine oxide **1b**, α-aminophosphine oxide dimethyl acetal (**4b**, R = Ph, R^1^ = Et, R^2^ = Me) was isolated in 92% yield (Scheme 1, Table 1, entry 2), while the addition of methanol (**2a**) to functionalized 2*H*-azirine-phosphine oxide **1c** furnished 81% of α-aminophosphine oxide dimethyl acetal (**4c**, R = R^1^ = Ph, R^2^ = Me) (Scheme 1, Table 1, entry 3).

A rational mechanism for the formation of α-aminophosphine oxide acetals **4** can be explained via initial nucleophilic addition of methanol (**2a**) at the carbon-nitrogen double bond of 2*H*-azirine **1** to give aziridine intermediate **3**. As reported previously [37,38,39,40,41,42,43,44], this nucleophilic addition is likely to arise in a diastereoselective way through the less hindered face. Subsequent ring opening to form α-aminophosphine oxide acetals **4** occurs with complete site selectivity at N–C3 bond, after nucleophilic attack of a second molecule of methanol. This behavior has been previously observed in the addition of methanol to methylene-2*H*-azirines [47], or more recently to an aryl substituted 2*H*-azirine [48].

This synthetic procedure could be broadened to the nucleophilic addition of methanol (**2a**) to 2*H*-azirine-phosphonates **1d** (R = O*^i^*Pr) and **1e** (R = OEt) under the same reaction conditions (Scheme 1). α-Aminophosphonate dimethyl acetals **4e** (R = O*^i^*Pr, R^1^ = R^2^ = Me, Table 1, entry 5) and **4f** (R = OEt, R^1^ = R^2^ = Me, Table 1, entry 6) were attained in moderate yields. Next, we tested other aliphatic alcohols in the nucleophilic addition to 2*H*-azirines **1**, under the optimal reaction conditions. For instance, 2*H*-azirine **1a** (R = Ph) reacted with ethanol (**2b**) in the presence of Et_3_N, producing the corresponding α-aminophosphine oxide diethyl acetal **4d** (see Table 1, entry 4).

We also explored the *N*-functionalization of α-aminophosphine oxide and phosphonate acetals **4** using the tosyl group as protecting group. Hence, sulfonylation of compounds **4** were achieved by treatment with *p*-toluenesulfonyl chloride (TsCl) in the presence of pyridine, in methylene chloride (CH_2_Cl_2_) at 25 °C. The corresponding *N*-tosylates **5** were obtained in moderate to good yields (Table 1, entries 7–9). This process might be performed in a one-pot operation from 2*H*-azirines **1** that would be appealing from an atom-economic alternative for carbon-heteroatom bond construction. Therefore, addition of ethanol (**2b**) to 2*H*-azirine **1e** in the presence of triethylamine afforded compound **4**, which, without isolation, was subjected to sulfonylation conditions to yield α-aminophosphonate diethyl acetal **5d** (Table 1, entry 10).

In addition, we studied deacetalization reaction of compounds **5** under acidic conditions in order to get β-keto-α-aminophosphonates **6** (Scheme 1). Treatment of α-aminophosphonate dimethyl acetal **5b** with a solution of 37% HCl in chloroform gave ketone **6** in 68% yield (Table 1, entry 11).

**Scheme 2:**
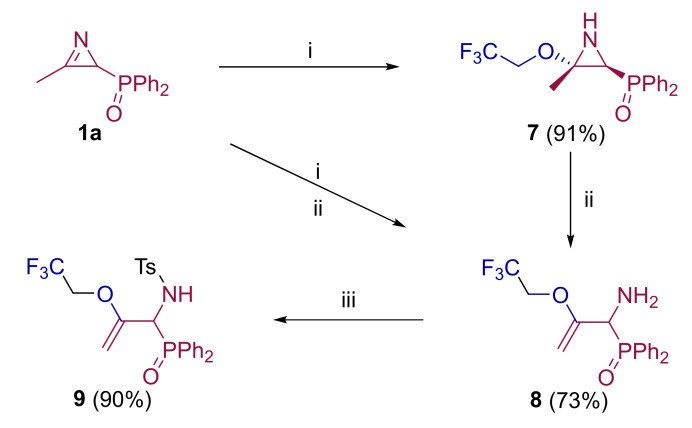
Addition of 2,2,2-trifluoroethanol to 2*H*-azirine **1a**. All synthesized compounds are racemic. Reagents and Conditions: (i) CF_3_CH_2_OH (**2c**, excess), Et_3_N, 4 Å M.S. or CF_3_CH_2_OH (**2c**, 2eq), Et_3_N, 4 Å M.S., CH_2_Cl_2_, 0 to 25 °C; (ii) CHCl_3_, Δ; (iii) TsCl, Py, CH_2_Cl_2_, 0 to 25 °C.

Reaction of other aliphatic alcohols with 2*H*-azirines **1** was also studied to check if these nucleophiles could provide a new entry to functionalized α-aminophosphorus derivatives. For this purpose we explored the reaction of 2*H*-azirine phosphine oxide **1a** with 2,2,2-trifluoroethanol (**2c**). However, unlike the α-aminophosphine oxide acetals **4** observed in the reaction of azirines **1** with methanol or ethanol, when 2*H*-azirine **1a** was treated, even under the standard conditions (see Scheme 1) or with two equivalents of trifluoroethanol (**2c**) in the presence of a base such as Et_3_N and CH_2_Cl_2_ as the solvent, aziridine **7** was obtained in very good yield (Scheme 2).

However, if the addition of trifluoroethanol (**2c**) to **1a** was performed in refluxing chloroform, [1-amino-2-(2,2,2-trifloroethoxy)allyl]diphenyl phosphine oxide **8** was exclusively obtained instead of aziridine **7** (Scheme 2). The spectroscopic data were in agreement with the assigned structure for compound **8** (see characterization data for compound **8**). The outcome of this conversion may be due to the initial formation of the corresponding aziridine **7**, resulting from the addition of trifluoroethanol (**2c**) to the imine bond of 2*H*-azirine **1a**. Subsequent C–C double bond formation and ring opening througH-N–C3 bond of aziridine afforded allylic α-aminophosphine oxide **8**. The former compound turned out to be unstable and therefore it was converted into the sulfonamide derivative **9** in 90% chemical yield by treatment with *p*-toluenesulfonyl chloride in the presence of pyridine (Scheme 2).

**Scheme 3:**
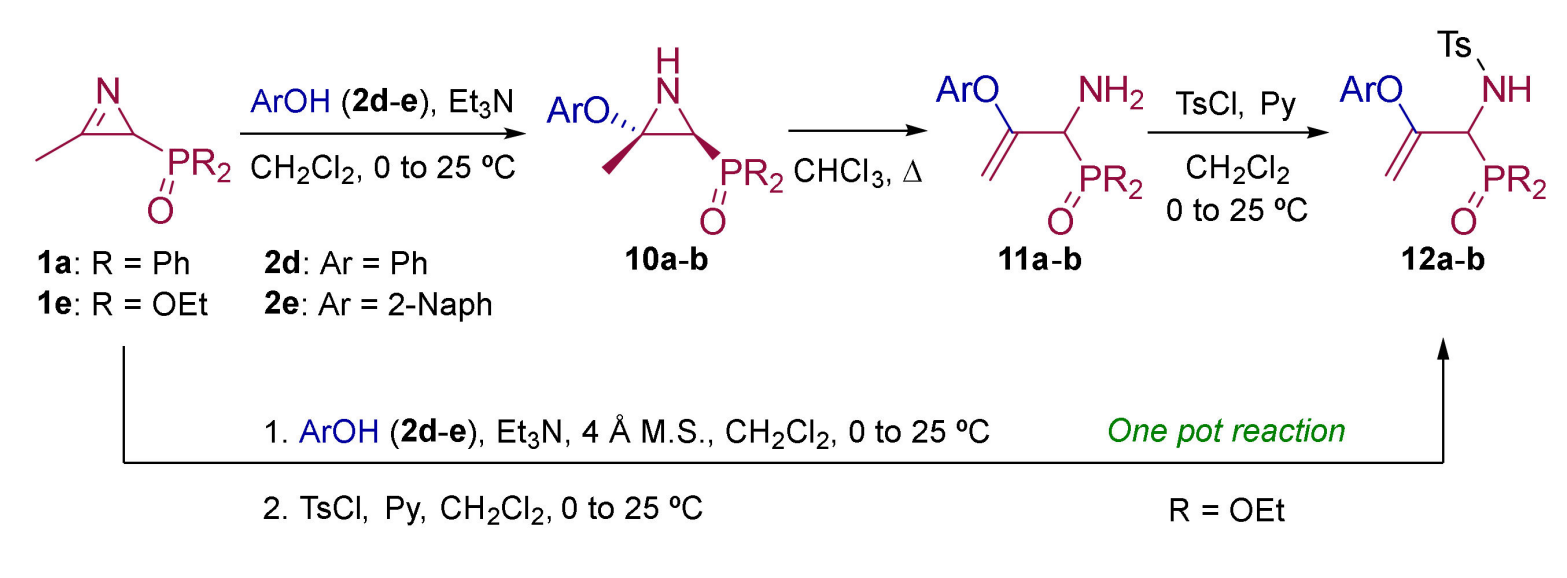
Synthesis of aziridines **10**, allyl α-aminophosphine oxides **11** and phosphonates **12.**

In order to limit the scope of the addition of *O*-nucleophilic reagents to 2*H*-azirines **1** and increase the diversity of substituents in our substrates, this methodology was extended to include the reactivity of phenols **2d**–**e** toward phosphorus substituted 2*H*-azirines **1**. For this purpose, the nucleophilic addition of phenol (**2d**) to 2*H*-azirine phosphine oxide **1a** was performed using Et_3_N as the base in CH_2_Cl_2_ to yield aziridine **10a** in moderate yield (Scheme 3, Table 2, entry 1).

Conversely, the addition of 2-naphtol (**2d**) to 2*H*-azirine **1a**, in the same reaction conditions, yielded a mixture of aziridine **10b** and allylic α-aminophosphine oxide **11b** (Scheme 3, Table 2, entry 2). Aziridine **10b** seemed to be very unstable and cleavage of the C3–N bond in the three-membered ring of **10b** promptly occurs to give allylic α-aminophosphine oxide **11b**. This observation was further confirmed when aziridine **10a**, or a mixture of aziridine **10b** and derivative **11b** was heated at refluxing chloroform. Under these reaction conditions, allylic α-aminophosphine oxide **11a** or **11b**, respectively, was obtained in good yields (Scheme 3, Table 2, entry 3 and 4). We then extended the scope of the nucleophilic addition of phenols (**2d**–**e**) to 2*H*-azirine phosphonate **1e**. In this case, only allylic α-aminophosphonates **11** were directly observed in the crude NMR, but owing to their instability, they could not be isolated. Hence, intermediates **11** derived from phosphonates were submitted to sulfonylation reaction in a one-pot procedure giving to the formation of allylic *N*-tosyl α-aminophosphonates **12a**–**b** (Scheme 3, Table 2, entries 5 and 6).

As far as we know, this regioselective process represents the first example of the synthesis of an allylic α-aminophosphorus derivative through the addition of oxygen nucleophiles to the carbon-nitrogen double bond of a phosphorus substituted 2*H*-azirines.

**Scheme 4:**
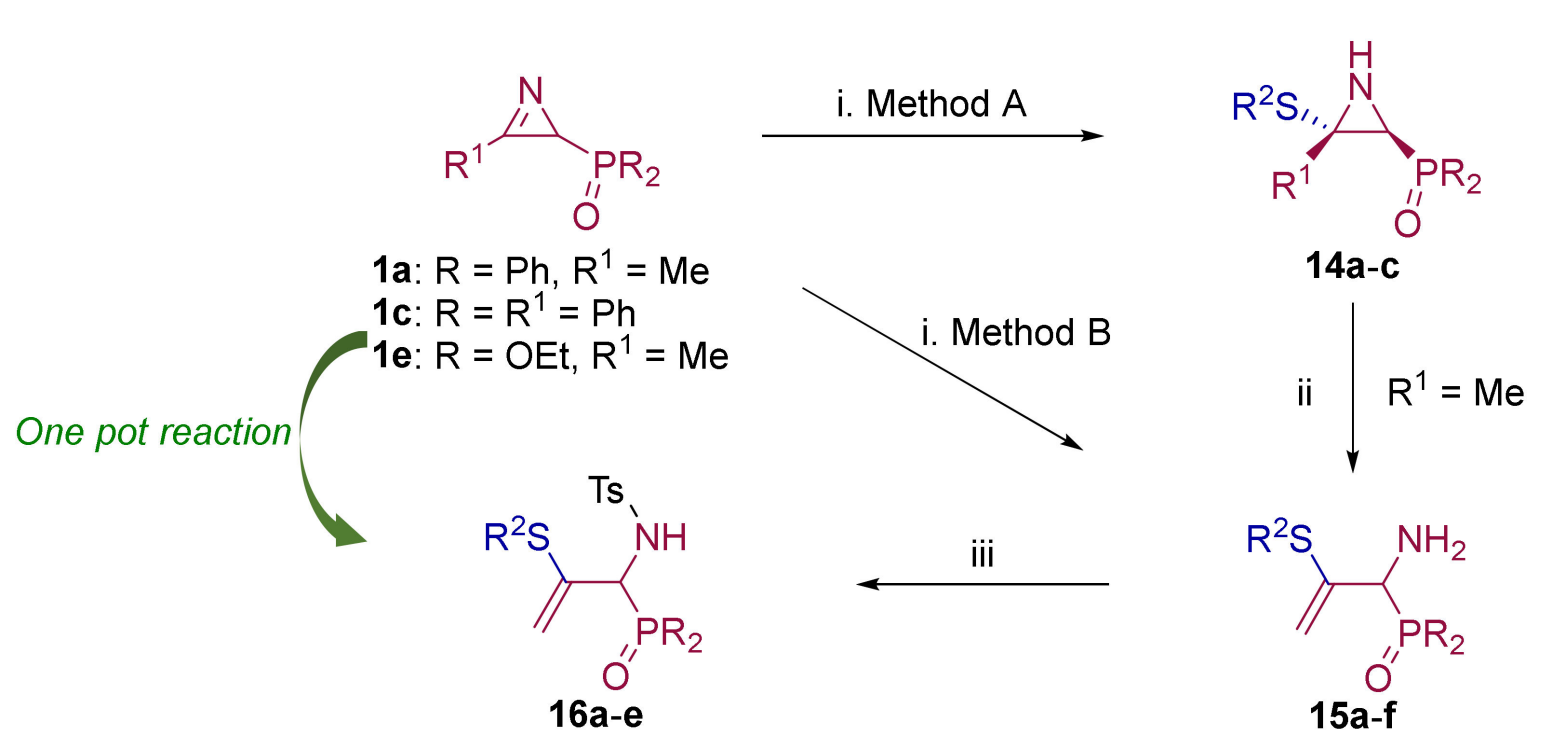
Synthesis of azidines **14** and allylic α-aminophosphine oxides and phosphonates **15** and **16**. All synthesized compounds are racemic. Reagents and Conditions: (i) Method A: R^2^SH (13), Et_3_N, 4 Å M.S., CH_2_Cl_2_, 0 °C to rt, Method B: R^2^SH (13), CH_2_Cl_2_, 0 °C, 48h; (ii) CHCl_3_, Δ; (iii) TsCl, Py, CH_2_Cl_2_, 0 °C. 13a: R^2^ = Ph; 13b: R^2^ = p-MeC_6_H_4_; 13c: R^2^ = p-FC_6_H_4_; 13d: R^2^ = p-MeOC_6_H_4_; 13e: R^2^ = Et.

Finally, in order to verify the potential of our synthetic methodology, we investigated the nucleophilic addition of sulfur nucleophiles to our phosphorus substituted 2*H*-azirines **1**. We anticipated that nucleophilic addition of thiophenols and thiols to 2*H*-azirines **1**, would supply a useful approach to the synthesis of aziridine derivatives **14** or even allylic α-aminophosphorus compounds **15**. Thus, as outlined in Scheme 4, in an initial experiment the nucleophilic addition of benzenethiol (**13a**) (R^2^ = Ph) to 2*H*-azirine phosphine oxide **1a** (R = Ph, R^1^ = Me) was readily attained using Et_3_N in dichloromethane at 25 °C (method A). Under these reaction conditions, aziridine derivative **14a** was achieved in 92% yield (Table 3, entry 1). This aziridine **14a** was very unstable since after crystallization the ^1^H-NMR spectrum showed different signals corresponding to aziridine **14a** and minor ones matching to the allylic α-aminophosphine oxide **15a**, formed through C3–N bond cleavage of aziridine ring. After a brief heating of the **14a** and **15a** compounds mixture in refluxing chloroform, only allylic α-aminophosphine oxide **15a** was observed by NMR (Table 3, entry 4). In our previous results [43], both trapping of aziridine intermediate nor detection in crude NMR could be accomplished, and only the allylic α-aminophosphine oxide **15a** was observed instead. Similarly, starting from 2*H*-azirine **1a** and 4-methylbenzenethiol (**13b**) (R^2^ = *p*-MeC_6_H_4_), a mixture of aziridine **14b** (Scheme 4, method A, Table 3, entry 2) and allylic α-aminophosphine oxide **15b** was isolated, which afforded **15b** after heating in refluxing chloroform.

Next, we carried out the addition of benzenethiol (**13a**) to 2*H*-azirine phosphine oxide **1c** (R = R^1^ = Ph) avoiding the C–C double bond formation and confirming the reaction mechanism. Thus, reaction of 2*H*-azirine **1c** with benzenethiol (**13a**) in the standard reaction conditions (method A) allowed us to get *E*-aziridine derivative **14c** stereoselectively (Scheme 4, Table 3, entry 2).

Optimization of the reaction conditions allowed us to achieve the successful regioselective formation of allylic α-aminophosphorus derivatives **15**. Therefore, when 2*H*-azirine **1a** reacted with 4-methylbenzenethiol (**13b**) (R^2^ = *p*-MeC_6_H_4_) without base at 0 °C for 48h (method B), only the formation of derivative **15b** was observed in 89% chemical yield (Scheme 4, Table 3, entry 5). Further scrutiny of the synthetic approach revealed that this process is also suitable to other substituted benzenethiols **13**. For instance, as outlined in Scheme 3, 2*H*-azirine phosphine oxide **1a** (R = Ph, R^1^ = Me) reacted with 4-fluorobenzenethiol (**13c**) (R^2^ = *p*-FC_6_H_4_) or 4-methoxybenzenethiol (**13d**) (R^2^ = *p*-MeOC_6_H_4_) for 48h at 0 °C, giving the corresponding allylic α-aminophosphine oxides **15c**–**d** (see Table 3, entries 6–7). This method also accommodates other 2*H*-azirines with phosphonate substitution, given that addition reaction of benzenethiol (**13a**) to 2*H*-azirine **1e** (R = OEt, R^1^ = Me) afforded allylic α-aminophosphonate **15e** in moderate yield (Table 3, entry 8). Likewise, aliphatic thiols such as ethanothiol (**13e**) satisfactorily reacted with 2*H*-azirine **1a** giving to the formation of derivative **15f** in a regioselective fashion (Table 3, entry 9).

Finally, we also examined the *N*-protection of allylic α-aminophosphine oxides and phosphonates **15**. As before, for this aim we used the tosyl group as *N*-protecting group. Then, compounds **15** were subjected to sulfonylation reaction using the standard conditions already used formerly (TsCl in the presence of pyridine, CH_2_Cl_2_ as the solvent, and at 25 °C), and allylic *N*-tosyl α-aminophosphine oxides and phosphonates **16a**–**c** were attained in good yields (Scheme 3, Table 3, entries 10–12). The process might be performed in a one-pot procedure from 2*H*-azirine **1e** when it reacts with *p*-substituted benzenethiols **13** at 0 °C for 48 h and subsequent treatment with *p*-toluenesulfonyl chloride in the presence of pyridine, yielding allylic *N*-tosyl α-aminophosphonates **16d–e** (Table 3, entries 13–14).

This approach represents a practical short regioselective route to allylic α-aminophosphine oxides and phophonates **15** via addition reaction of sulfur nucleophiles to phosphorus substituted 2*H*-azirines **1**. Moreover, *N*-functionalization by adding electron-withdrawing groups can be performed by *N*-tosylation of the corresponding derivatives **15**.

### 2.2. Biological Results

The cytotoxicity of the new α-aminophosphine oxide and phosphonate acetals **4** and **5**, β-keto-α-aminophosphonate **6**, aziridines **7**, **10** and **14**, and allylic α-aminophosphine oxides and phosphonates **8**, **9**, **11**, **12**, **15** and **16** was investigated in vitro by checking their antiproliferative activities against the human cancer cell line A549 (carcinomic human alveolar basal epithelial cells). Human colon carcinoma cell line (RKO) was also used to test the antiproliferative activity of some of our compounds. In order to assess growth inhibition, cell counting kit (CCK-8) assay was employed. Cell proliferation inhibitory activities as IC_50_ values for all synthesized compounds and chemotherapeutic doxorubicin (DOX) are displayed in Table 4 and Table 5. Likewise, healthy lung cells, such as MRC-5 non-malignant lung fibroblasts were tested to study the selectivity of the cytotoxicity [49].

Primary **4** and secondary α-aminophosphine oxides and phosphonate acetals **5** demonstrated cytotoxic effect when evaluated against A549 cell line in vitro (Table 4, entries 2–11). For instance, compounds **4** showed IC_50_ values between 1.3 ± 0.10 and 21.3 ± 0.22 µM, with the most effective compound being α-aminophosphonate dimethyl acetal **4f** (Table 4, entry 7) with an IC_50_ value of 1.3 ± 0.10 µM. Similar activities was observed for secondary α-aminophosphine oxides and phosphonate acetals **5** with IC_50_ values between 1.7 ± 0.30 and 8.2 ± 0.23 µM, with the most cytotoxic compound being *N*-tosyl α-aminophosphonate dimethyl acetal **5b** (Table 4, entry 9). The hydrolysis of acetal group seemed not to have any effect since β-keto-α-aminophosphonate **6** do not exhibited any toxicity toward A549 (Table 4, entry 12).

Concerning allylic α-aminophosphorus derivatives obtained from the addition of trifluoroethanol (**2c**) or phenols (**2d**–**e**), besides allylic α-aminophosphine oxide **8** (Table 4, entry 15) which do not exhibited any toxicity effect toward A549, derivatives **11a**–**b** even allylic *N*-tosyl α-aminophosphine oxide **9** and phosphonates **12a**–**b** displayed very good cytotoxicity (Table 4, entries 16–17, 18, 19–20, respectively).

Regarding the new oxygen and sulfur-containing aziridine derivatives **7**, **10a**, (Table 4) and **14c** (Table 5) against A549 cell line in vitro, diphenyl [3-phenyl-3-(phenylthio)aziridin-2-yl]phosphine oxide (**14c**) was the most cytotoxic compound with an IC_50_ value of 1.1 ± 0.32 µM (Table 5, entry 2).

We next studied allylic α-aminophosphorus derivatives with sulfur substituents **15** and **16** into their cytotoxicity against A549 cell line (Table 5). All of them showed good cytotoxicity. For instance, IC_50_ values between 0.1 ± 0.08 and 7.2 ± 0.49 µM was observed, being allylic α-aminophosphine oxide **15c** (Table 5, entry 5) the most effective compound for primary allylic α-aminophosphorus derivatives **15**. However, for allylic *N*-tosyl α-aminophosphorus derivatives **16**, the most cytotoxic compound with an IC_50_ value of 0.2 ± 0.07 µM was derivative **16c** (Table 5, entry 10).

Some of our synthesized compounds were tested as antiproliferative agents toward the RKO cell line. For instance, α-aminophosphine oxide acetal **4a**, allylic α-aminophosphine oxide **11a** (Table 4, entries 2, 16), and allylic α-aminophosphine oxides **15a**, **15c**, and **16c** (Table 5, entries 3, 5, and 10) do not exhibited any toxicity toward RKO. However, good cytotoxicity effect was observed for aziridine phosphine oxide **14c**, with an IC_50_ value of 9.7 ± 1.4 µM (Table 5, entry 2). Additionally, MRC-5 non-malignant lung fibroblasts were tested to explore selective toxicity [26]. Except for some allylic α-aminophosphorus derivatives, which displayed moderate cytotoxicity, nearly all the synthesized α-aminophosphorus derivatives, aziridines, and doxorubicin did not exhibit toxicity toward MRC-5 cell line (see Table 4 and Table 5). Additionally, aziridine **14c**, (Table 5, entry 2) which showed good cytotoxicity against A549 and RKO cell lines, also exhibited good cytotoxicity toward MRC-5 cell line.

## 3. Materials and Methods

### 3.1. Chemistry

#### 3.1.1. General Information

Solvents for extraction and chromatography were of technical grade. All solvents used in reactions were freshly distilled and dried over 4 Å molecular sieves before use. All other solvents and reagents were obtained from commercial sources and recrystallized or distilled as necessary or used without further purification. All reactions were performed under an atmosphere of dry nitrogen. Melting points were determined with an IA9100 Digital Melting Point Apparatus (Electrothermal; Cole-Parmer, Staffordshire, UK) and are uncorrected. IR spectra were measured as neat solids on a Nicolet iS10 spectrometer (Thermo Scientific, Waltham, MA, USA). Absorbance frequencies are given at maximum of intensity in cm^−1^. High-resolution mass spectra (HRMS) were obtained by positive-ion electrospray ionization (ESI) method with a time of flight Q-TOF system (Agilent 6530, Agilent Technologies, Santa Clara, CA, USA). Data are reported in the form *m/z* (intensity relative to base = 100). ^1^H- (300, 400 MHz), ^13^C- (75, 100 MHz), ^19^F- (282 MHz), and ^31^P-NMR (120, 160 MHz) spectra were recorded on a VXR 300 MHz (Varian, Agilent Technologies, Santa Clara, CA, USA) or Avance 400 MHz (Bruker Corporation Billerina, MA, USA) spectrometers, respectively, in CDCl_3_ or DMSO-*d_6_*, as specified below at 25 °C. Chemical shifts (*δ*_H_) are reported in parts per million (ppm) with the internal chloroform signal at 7.24 ppm as standard for ^1^H-NMR. Chemical shifts (*δ*_C_ and *δ*_P_) are reported in parts per million (ppm) with the internal chloroform signal at 77.0 ppm as standard for ^13^C-NMR; the external fluorotrichloromethane (CFCl_3_) signal at 0.0 ppm as standard for ^19^F-NMR; or the external H_3_PO_4_ (50%) signal at 0.0 ppm as standard for ^31^P-NMR. All coupling constants (*J*) values are given in Hz. ^19^F and ^13^C NMR spectra were recorded in a broadband decoupled mode from hydrogen nuclei. Distortionless Enhanced Polarization Transfer (DEPT) supported peak assignments for ^13^C NMR. The data is being reported as (s = singlet, d = doublet, t = triplet, q = quartet, m = multiplet, dd = double doublet, bs = broad singlet). Chromatographic purification was performed as flash chromatography using commercial grades of silica gel finer than 230 mesh with pressure. Analytical thin layer chromatography (TLC) was performed on precoated silica gel 60 F_254_ TLC aluminium plates (Merck, Darmstad, Germany) and spots visualized with UV light or permanganate stain. 2*H*-Azirines **1** were prepared according to literature procedures [25,29,30,52].

#### 3.1.2. Experimental Procedure and Characterization Data for Compounds **4**, **5** and **6**

##### General Procedure and Spectral Data for the Addition of Aliphatic Alcohols to Functionalized 2H-Azirines **1**

To a 0 °C solution of 2*H*-azirine **1** (5 mmol, 1 eq) in aliphatic alcohol **2a**–**b** (25 mL) and 4 Å M.S., Et_3_N (1.4 mL, 10 mmol, 2 eq) was added dropwise. The reaction mixture was allowed to reach 25 °C and stirred until TLC showed the disappearance of starting 2*H*-azirine **1** (24 h). 4 Å M.S. was filtered through a sintered glass vacuum filtration funnel with Celite and washed with alcohol. The filtrate was concentrated to dryness in vacuum and the resulting residue was diluted with CH_2_Cl_2_ (15 mL). The organic phase was washed with water (3 × 15 mL) and extracted with CH_2_Cl_2_. The organic layer was dried over anhydrous MgSO_4_, filtered and concentrated to dryness in vacuum. The crude products **4** were purified by crystallization or by flash-column chromatography.

*(1-Amino-2,2-dimethoxypropyl) diphenylphosphine oxide* (**4a**), (1.18 g, 74%) obtained as a yellow solid from 2*H*-azirine **1a** (1.28 g, 5 mmol) using MeOH as described in the general procedure. The crude product was purified by crystallization from Et_2_O to afford the title compound **4a**. mp 125–127 °C; IR (neat) *v*_max_ 3386, 3056, 2990, 2942, 2832, 1442, 1385, 1179, 1116, 1040, 723, 701 cm^−1^; ^1^H-NMR (300 MHz, CDCl_3_) *δ* 7.80–7.65 (m, 4H, Ar*H*), 7.39–7.21 (m, 6H, Ar*H*), 3.67 (d, ^2^*J*_PH_ = 5.5 Hz, 1H, C*H*–P), 3.03 (s, 3H, OC*H*_3_), 2.64 (s, 3H, OC*H*_3_), 1.47 (bs, 2H, N*H_2_*), 1.32 (s, 3H, C*H*_3_) ppm; ^13^C {1H}-NMR (75 MHz, CDCl_3_) *δ* 133.0 (d, ^1^*J*_PC_ = 97.6 Hz, C_quat_), 133.0 (d, ^1^*J*_PC_ = 97.9 Hz, C_quat_), 131.4, 131.3, 131.1, 131.0, 130.9, 130.9, 128.4 (d, *J*_PC_ = 11.3 Hz), 127.6 (d, *J*_PC_ = 11.8 Hz) (C_Ar_), 102.5 (d, ^2^*J*_PC_ = 4.9 Hz, C_quat_), 54.0 (d, ^1^*J*_PC_ = 75.6 Hz, CH-P), 48.1 (OCH_3_), 47.6 (OCH_3_), 18.4 (CH_3_) ppm; ^31^P-NMR (120 MHz, CDCl_3_) *δ* 30.9 ppm; ESI-HRMS (CI) *m/z* calcd. for C_17_H_22_NNaO_3_P ([M + Na]^+^) 342.1235, found 342.1230.


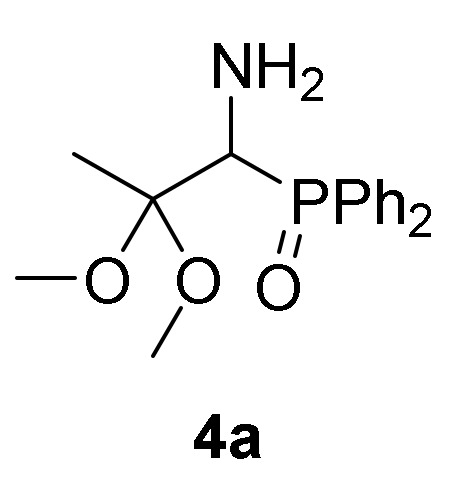


*(1-Amino-2,2-dimethoxybutyl)diphenylphosphine oxide* (**4b**), (1.53 g, 92%) obtained as a yellow solid from 2*H*-azirine **1b** (1.35 g, 5 mmol) using MeOH as described in the general procedure. The crude product was purified by crystallization from Et_2_O to afford the title compound **4b**. mp 96–97 °C; IR (neat) *v*_max_ 3322, 3060, 2939, 2822, 1442, 1182, 1159, 1097, 1046 cm^−1^; ^1^H-NMR (400 MHz, CDCl_3_) *δ* 7.91–7.81 (m, 4H, Ar*H*), 7.45–7.37 (m, 6H, Ar*H*), 3.84 (d, ^2^*J*_PH_ = 6.6 Hz, 1H, C*H*-P), 3.10 (s, 3H, OC*H*_3_), 2.97 (s, 3H, OC*H*_3_), 2.01–1.81 (m, 2H, C*H_2_*), 1.74 (bs, 2H, N*H_2_*), 0.98 (t, ^3^*J*_HH_ = 7.5 Hz, 3H, C*H*_3_) ppm; ^13^C {1H}-NMR (100 MHz, CDCl_3_) *δ* 133.5 (d, ^1^*J*_PC_ = 97.0 Hz, C_quat_), 133.2 (d, ^1^*J*_PC_ = 97.2 Hz, C_quat_), 131.4, 131.4, 131.3, 131.0, 131.0, 128.5 (d, *J*_PC_ = 11.2 Hz), 127.7 (d, *J*_PC_ = 11.9 Hz) (C_Ar_), 103.2 (d, ^2^*J*_PC_ = 3.3 Hz, C_quat_), 55.5 (d, ^1^*J*_PC_ = 75.4 Hz, CH-P), 49.3 (OCH_3_), 48.5 (OCH_3_), 26.1 (CH_2_), 8.8 (CH_3_) ppm; ^31^P-NMR (160 MHz, CDCl_3_) *δ* 30.3 ppm; ESI-HRMS (CI) *m/z* calcd. for C_18_H_24_NNaO_3_P ([M + Na]^+^) 356.1391, found 356.1386.


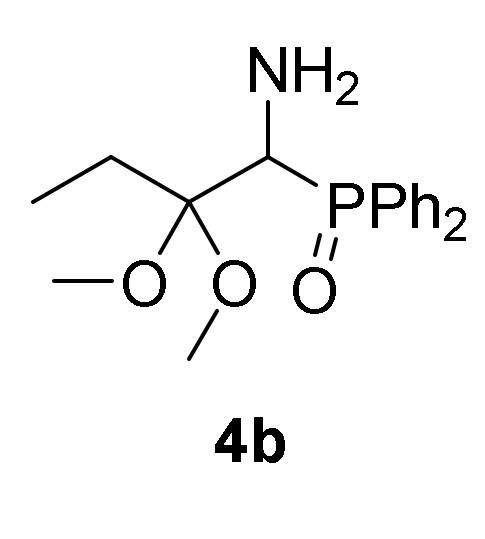


*(1-Amino-2,2-dimethoxy-2-phenylethyl)diphenylphosphine oxide* (**4c**), (1.54 g, 81%) obtained as a yellow solid from 2*H*-azirine **1c** (1.59 g, 5 mmol) using MeOH as described in the general procedure. The crude product was purified by flash-column chromatography (SiO_2_, EtOAc/hexane 50:50) to afford the title compound **4c**. mp 113–115 °C; IR (neat) *v*_max_ 3462, 3060, 2984, 2939, 1448, 1438, 1372, 1242, 1116, 1097, 1046 cm^−1^; ^1^H-NMR (400 MHz, CDCl_3_) *δ* 7.91–7.20 (m, 15H, Ar*H*), 4.12 (d, ^2^*J*_PH_ = 8.6 Hz, 1H, C*H*-P), 3.23 (s, 3H, OC*H*_3_), 3.15 (s, 3H, OC*H*_3_), 1.68 (bs, 2H, N*H_2_*) ppm; ^13^C {1H}-NMR (75 MHz, CDCl_3_) *δ* 137.2, 134.6, 133.3, 132.9, 131.6, 131.3, 131.2, 131.0, 130.9, 130.9, 130.8, 128.0, 127.9, 127.9, 127.8, 127.8, 127.6, 127.5 (C_Ar_), 103.7 (d, ^2^*J*_PC_ = 3.8 Hz, C_quat_), 56.7 (d, ^1^*J*_PC_ = 78.2 Hz, CH-P), 49.8 (OCH_3_), 48.8 (OCH_3_) ppm; ^31^P-NMR (120 MHz, CDCl_3_) *δ* 27.8 ppm; ESI-HRMS (CI) *m/z* calcd. for C_22_H_24_NNaO_3_P ([M + Na]^+^) 404.1391, found 404.1386.


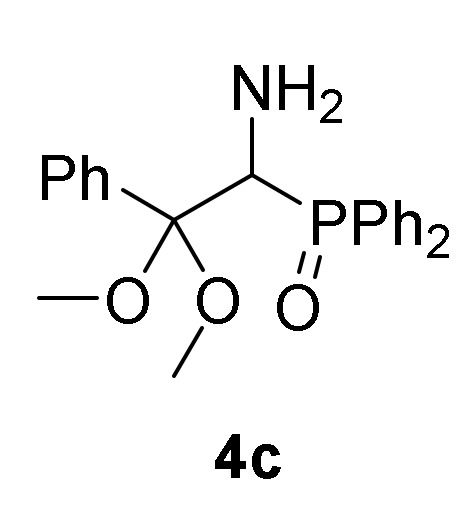


*(1-Amino-2,2-diethoxypropyl)diphenylphosphine oxide* (**4d**), (0.97 g, 56%) obtained as a yellow solid from 2*H*-azirine **1a** (1.28 g, 5 mmol) using EtOH as described in the general procedure. The crude product was purified by flash-column chromatography (SiO_2_, EtOAc) to afford the title compound **4d**. mp 126–128 °C; IR (neat) *v*_max_ 3386, 3326, 2974, 2927, 2889, 1438, 1385, 1182, 1120, 1068, 1049, 951, 723, 695 cm^−1^; ^1^H-NMR (300 MHz, CDCl_3_) *δ* 7.90–7.76 (m, 4H, Ar*H*), 7.44–7.34 (m, 6H, Ar*H*), 3.80 (d, ^2^*J*_PH_ = 5.0 Hz, 1H, C*H*-P), 3.48–3.38 (m, 2H, C*H_2_*), 3.24–3.00 (m, 2H, C*H_2_*), 1.59 (bs, 2H, N*H_2_*), 1.49 (s, 3H, C*H*_3_), 1.12 (t, ^3^*J*_HH_ = 7.0 Hz, 3H, C*H*_3_), 0.51 (t, ^3^*J*_HH_ = 7.0 Hz, 3H, C*H*_3_) ppm; ^13^C {1H}-NMR (75 MHz, CDCl_3_) *δ* 133.5 (d, ^1^*J*_PC_ = 97.8 Hz, C_quat_), 133.5 (d, ^1^*J*_PC_ = 97.8 Hz, C_quat_), 131.4, 131.3, 131.0, 130.9, 130.8, 128.5 (d, *J*_PC_ = 11.1 Hz), 127.6 (d, *J*_PC_ = 11.9 Hz) (C_Ar_), 102.4 (d, ^2^*J*_PC_ = 4.3 Hz, C_quat_), 56.2 (OCH_2_), 55.1 (OCH_2_), 54.63 (d, ^1^*J*_PC_ = 75.5 Hz, CH-P), 19.5 (CH_3_), 15.2 (CH_3_), 14.2 (CH_3_) ppm; ^31^P-NMR (120 MHz, CDCl_3_) *δ* 31.1 ppm; ESI-HRMS (CI) *m/z* calcd. for C_19_H_26_NNaO_3_P ([M + Na]^+^) 370.1548, found 370.1543.


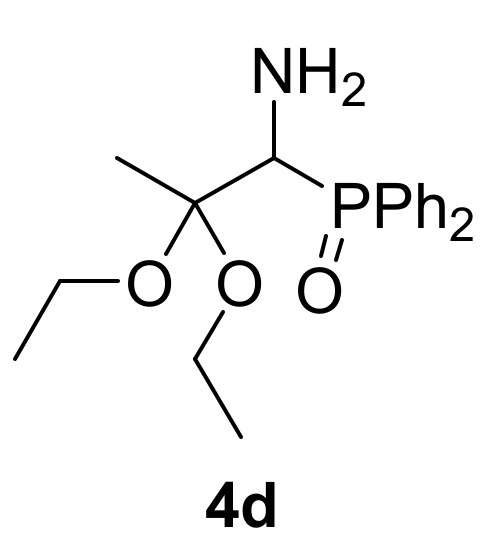


*Diisopropyl (1-amino-2,2-dimethoxypropyl)phosphonate* (**4e**), (0.98 g, 69%) obtained as a yellow oil from 2*H*-azirine **1d** (1.10 g, 5 mmol) using MeOH as described in the general procedure. The crude product was purified by flash-column chromatography (SiO_2_, AcOEt) to afford the title compound **4e**. IR (neat) *v*_max_ 3300, 2971, 2933, 1467, 1381, 1239, 983 cm^−1^; ^1^H-NMR (400 MHz, CDCl_3_) *δ* 4.68–4.58 (m, 2H, OC*H*), 3.19–3.16 (m, 1H, C*H*-P), 3.15 (s, 3H, OC*H*_3_), 3.08 (s, 3H, OC*H*_3_), 1.57 (bs, 2H, N*H_2_*), 1.36 (s, 3H, C*H*_3_), 1.25–1.21 (m, 12H, CH(C*H*_3_)_2_) ppm; ^13^C {1H}-NMR (100 MHz, CDCl_3_) *δ* 101.8 (d, ^2^*J*_PC_ = 7.8 Hz, C_quat_), 70.8 (d, ^2^*J*_PC_ = 6.8 Hz, OCH), 70.2 (d, ^2^*J*_PC_ = 7.2 Hz, OCH), 53.0 (d, ^1^*J*_PC_ = 153.4 Hz), 48.4 (OCH_3_), 47.5 (OCH_3_), 24.2 (d, ^3^*J*_PC_ = 2.7 Hz, CH_3_), 24.0 (d, ^3^*J*_PC_ = 3.4 Hz, CH_3_), 23.7 (d, ^3^*J*_PC_ = 5.2 Hz, CH_3_), 23.5 (d, ^3^*J*_PC_ = 5.8 Hz, CH_3_), 17.7 (CH_3_) ppm; ^31^P-NMR (160 MHz, CDCl_3_) *δ* 23.8 ppm; ESI-HRMS (CI) *m/z* calcd. for C_10_H_23_NO_4_P ([M–OMe]^+^) 252.1370, found 252.1361.


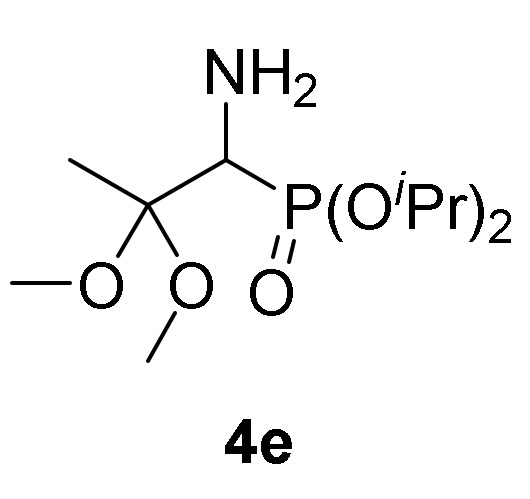


*Diethyl (1-amino-2,2-dimethoxypropyl)phosphonate* (**4f**), (0.78 g, 61%) obtained as a brown oil from 2*H*-azirine **1e** (0.96 g, 5 mmol) using MeOH as described in the general procedure. The crude product was purified by flash-column chromatography (SiO_2_, EtOAc/MeOH 99:1) to afford the title compound **4f**. IR (neat) *v*_max_ 3466, 3319, 2928, 2945, 2905, 1650, 1454, 1236, 1022 cm^−1^; ^1^H-NMR (400 MHz, CDCl_3_) *δ* 4.18–4.05 (m, 4H, OC*H_2_*), 3.32 (d, ^2^*J*_PH_ = 17.6 Hz, 1H, C*H*-P), 3.24 (s, 3H, OC*H*_3_), 3.16 (s, 3H, OC*H*_3_), 1.61 (bs, 2H, N*H_2_*), 1.44 (s, 3H, C*H*_3_), 1.31 (dt, ^3^*J*_HH_ = 7.0 Hz, ^3^*J*_PH_ = 0.5 Hz, 3H, C*H*_3_), 1.30 (dt, ^3^*J*_HH_ = 7.1 Hz, ^3^*J*_PH_ = 0.5 Hz, 3H, C*H*_3_) ppm; ^13^C {1H}-NMR (100 MHz, CDCl_3_) *δ* 101.8 (d, ^2^*J*_PC_ = 8.1 Hz, C_quat_), 62.5 (d, ^2^*J*_PC_ = 6.6 Hz, OCH_2_), 61.8 (d, ^2^*J*_PC_ = 6.8 Hz, OCH_2_), 52.7 (d, ^1^*J*_PC_ = 152.0 Hz, CH-P), 48.8 (OCH_3_), 47.8 (OCH_3_), 17.9 (CH_3_), 16.5 (d, ^3^*J*_PC_ = 4.9 Hz, CH_3_), 16.4 (d, ^3^*J*_PC_ = 4.9 Hz, CH_3_) ppm; ^31^P-NMR (160 MHz, CDCl_3_) *δ* 25.9 ppm; ESI-HRMS (CI) *m/z* calcd. for C_8_H_19_NO_4_P ([M–OMe]^+^) 224.1057, found 224.1052.


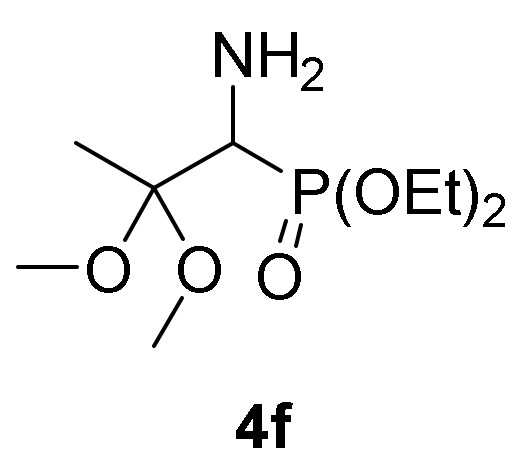


##### General Procedure and Spectral Data for the N-Tosyl Functionalization of α-Aminophosphine Oxide and Phosphonate Acetals **4**

*p*-Toluenesulfonyl chloride (1 g, 5.5 mmol, 1.1 eq) and pyridine (2.42 mL, 30 mmol, 6 eq) were added to a 0 °C solution of α-aminophosphine oxide or phosphonate acetal 4 (5 mmol, 1 eq) in CH_2_Cl_2_ (25 mL). The reaction mixture was allowed to reach 25 °C and stirred until TLC showed the disappearance of starting compound 4. The crude product was washed twice with a 2M HCl solution (15 mL) and water (15 mL) and extracted with CH_2_Cl_2_ (15 mL). The organic layers were dried over anhydrous MgSO_4_, filtered and concentrated to dryness in vacuum. The crude products 5 were purified by crystallization or by flash-column chromatography.

*N-[1-(Diphenylphosphoryl)-2,2-dimethoxypropyl]-4-methylbenzenesulfonamide* (**5a**), (1.89 g, 80%) obtained as a yellow solid from α-aminophosphine oxide **4a** (1.60 g, 5 mmol) after 24 h at 25 °C as described in the general procedure. The crude product was purified by crystallization from Et_2_O/CH_2_Cl_2_ 50:50 to afford the title compound **5a**. mp 205–207 °C; IR (neat) *v*_max_ 3440, 2990, 2939, 2885, 1445, 1331, 1182, 1157, 1119, 1097, 1046 cm^−1^; ^1^H-NMR (400 MHz, CDCl_3_) *δ* 7.92–7.06 (m, 14H, Ar*H*), 4.80 (t, ^3^*J*_PH_ = 18.1 Hz, ^3^*J*_HH_ = 9.1 Hz, 1H, C*H*-P), 2.92 (s, 3H, OC*H*_3_), 2.60 (s, 3H, OC*H*_3_), 2.31 (s, 3H, C*H*_3_), 1.40 (s, 3H, C*H*_3_), ppm; ^13^C {1H}-NMR (100 MHz, CDCl_3_) *δ* 142.2, 139.7, 133.8, 132.8, 132.0, 131.6, 131.3, 131.2, 131.2, 131.1, 131.1, 131.0, 130.9, 128.8, 128.5, 128.4, 127.8, 127.7, 126.5 (C_Ar_), 102.4 (d, ^2^*J*_PC_ = 7.1 Hz, C_quat_), 55.6 (d, ^2^*J*_PC_ = 74.1 Hz, CH-P), 48.3 (OCH_3_), 48.0 (OCH_3_), 21.4 (CH_3_), 19.0 (CH_3_) ppm; ^31^P-NMR (120 MHz, CDCl_3_) *δ* 31.5 ppm; ESI-HRMS (CI) *m/z* calcd. for C_24_H_28_NNaO_5_PS ([M+Na]^+^) 496.1323, found 496.1318.


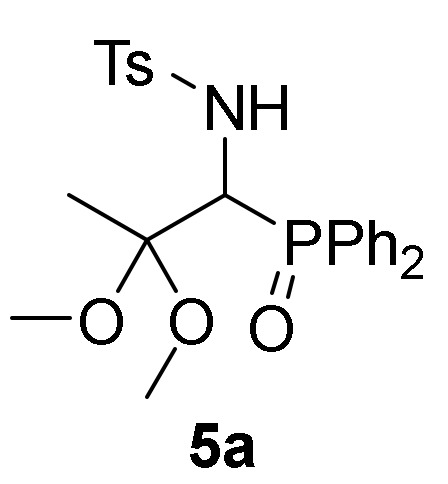


*Diisopropyl [2,2-dimethoxy-1-((4-methylphenyl)sulfonamido)propyl]phosphonate* (**5b**), (1.36 g, 62%) obtained as a white solid from α-aminophosphonate **4e** (1.42 g, 5 mmol) after 24 h at 25 °C as described in the general procedure. The crude product was purified by flash-column chromatography (SiO_2_, EtOAc/hexane 16:84) and crystallization from Et_2_O to afford the title compound **5b**. mp 137–139 °C; IR (neat) *v*_max_ 3161, 2923, 1590, 1378, 1328, 1176, 989 cm^−1^; ^1^H-NMR (400 MHz, CDCl_3_) *δ* 7.73 (d, ^3^*J*_HH_ = 8.3 Hz, 2H, Ar*H*), 7.24 (d, ^3^*J*_HH_ = 8.0 Hz, 2H, Ar*H*), 5.04 (dd, ^3^*J*_HH_ = 9.1 Hz, ^3^*J*_PH_ = 6.7 Hz, 1H, N*H*), 4.76–4.64 (m, 2H, OC*H*), 4.10 (dd, ^3^*J*_HH_ = 9.1 Hz, ^2^*J*_PH_ = 21.4 Hz, 1H, C*H*-P), 3.14 (s, 3H, OC*H*_3_), 2.88 (s, 3H, OC*H*_3_), 2.38 (s, 3H, C*H*_3_), 1.36 (s, 3H, C*H*_3_), 1.32–1.29 (m, 12H, CH(C*H*_3_)_2_) ppm; ^13^C {1H}-NMR (75 MHz, CDCl_3_) *δ* 142.6, 139.5, 129.0, 126.9 (C_Ar_), 101.3 (d, ^2^*J*_PC_ = 12.0 Hz, C_quat_), 71.9 (d, ^2^*J*_PC_ = 7.0 Hz, OCH), 71.8 (d, ^2^*J*_PC_ = 7.2 Hz, OCH), 55.0 (d, ^1^*J*_PC_ = 154.1 Hz, CH-P), 49.1 (OCH_3_), 48.1 (OCH_3_), 23.8 (CH_3_), 23.7 (CH_3_), 23.6 (CH_3_), 23.5 (CH_3_), 21.5 (CH_3_), 18.4 (CH_3_) ppm; ^31^P-NMR (120 MHz, CDCl_3_) *δ* 18.3 ppm; ESI-HRMS (CI) *m/z* calcd. for C_17_H_29_NO_6_PS ([M–OMe]^+^) 406.1459, found 406.1450.


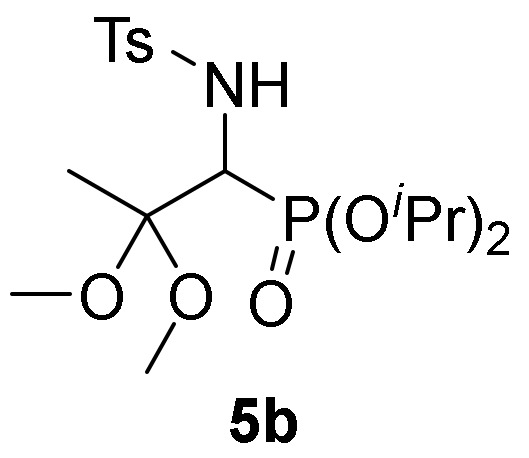


*Diethyl [2,2-dimethoxy-1-((4-methylphenyl)sulfonamido)propyl]phosphonate* (**5c**), (1.31 g, 64%) obtained as a pale yellow oil from α-aminophosphonate **4f** (1.28 g, 5 mmol) after 3 h at 25 °C as described in the general procedure. The crude product was purified by flash-column chromatography (SiO_2_, EtOAct/hexane 20:80) to afford the title compound **5c**. IR (neat) *v*_max_ 3174, 2987, 1331, 1239, 1220, 1157, 995 cm^−1^; ^1^H-NMR (400 MHz, CDCl_3_) *δ* 7.74 (d, ^3^*J*_HH_ = 8.4 Hz, 2H, Ar*H*), 7.21 (d, ^3^*J*_HH_ = 8.0 Hz, 2H, Ar*H*), 5.98 (dd, ^3^*J*_HH_ = 9.4 Hz, ^3^*J*_PH_ = 5.2 Hz, 1H, N*H*), 4.15–4.00 (m, 5H, C*H*-P and OC*H_2_*), 3.14 (s, 3H, OC*H*_3_), 2.86 (s, 3H, OC*H*_3_), 2.36 (s, 3H, C*H*_3_), 1.36 (s, 3H, C*H*_3_), 1.26 (t, ^3^*J*_HH_ = 7.1 Hz, 3H, C*H*_3_), 1.24 (t, ^3^*J*_HH_ = 7.1 Hz, 3H, C*H*_3_) ppm; ^13^C {1H}-NMR (75 MHz, CDCl_3_) *δ* 142.3, 139.7, 128.8, 126.7 (C_Ar_), 101.3 (d, ^2^*J*_PC_ = 12.7 Hz, C_quat_), 62.9 (d, ^2^*J*_PC_ = 6.6 Hz, OCH_2_), 62.9 (d, ^2^*J*_PC_ = 7.1 Hz, OCH_2_), 54.2 (d, ^1^*J*_PC_ = 153.6 Hz, CH-P), 49.0 (OCH_3_), 48.0 (OCH_3_), 21.4 (CH_3_), 18.4 (CH_3_), 16.2 (CH_3_), 16.2 (CH_3_) ppm; ^31^P-NMR (120 MHz, CDCl_3_) *δ* 20.3 ppm; ESI-HRMS (CI) *m/z* calcd. for C_15_H_25_NO_6_PS ([M–OMe]^+^) 378.1146, found 378.1133.


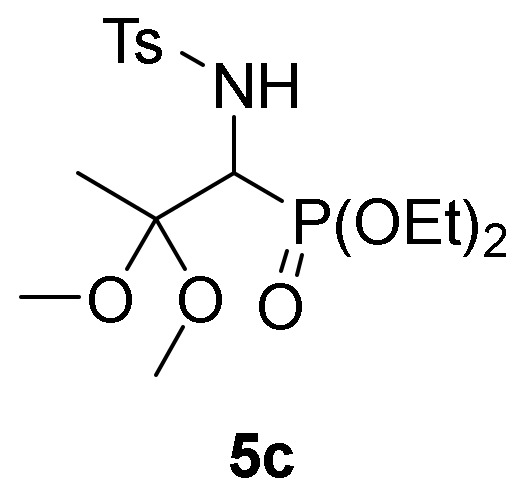


##### One Pot Procedure for the Synthesis of N-tosyl-α-Aminophosphonate Acetal **5d**

To a 0 °C solution of 2*H*-azirine **1e** (0.96 g, 5 mmol) in EtOH (25 mL) and 4 Å M.S., Et_3_N (1.4 mL, 10 mmol, 2 eq) was added dropwise. The reaction mixture was allowed to reach 25 °C and stirred for 24 h until TLC showed the disappearance of starting 2*H*-azirine **1e**. 4 Å M.S. was filtered through a sintered glass vacuum filtration funnel with celite and washed with EtOH. The filtrate was concentrated to dryness in vacuum and the resulting residue was diluted with CH_2_Cl_2_ (15 mL). The organic phase was washed with water (3 × 15 mL) and extracted with CH_2_Cl_2_. The organic layer was dried over anhydrous MgSO_4_, filtered and concentrated to dryness under vacuum. To a 0 °C solution of the crude product **4** in CH_2_Cl_2_ (25 mL) was directly added *p*-toluenesulfonyl chloride (1 g, 5.5 mmol, 1.1 eq) and pyridine (2.42 mL, 30 mmol, 6 eq). The reaction mixture was allowed to reach 25 °C and stirred for 24 h. The crude product was washed twice with a 2M HCl solution (15 mL) and water (15 mL) and extracted with CH_2_Cl_2_ (15 mL). The organic layer was dried over anhydrous MgSO_4_, filtered and concentrated to dryness in vacuum. The crude product **5d** was purified by crystallization from Et_2_O.

*Diethyl [2,2-diethoxy-1-((4-methylphenyl)sulfonamido)propyl]phosphonate* (**5d**), (1.53 g, 70%) obtained as a white solid. mp 126–128 °C; IR (neat) *v*_max_ 3434, 3189, 2979, 2931, 2885, 1560, 1474, 1463, 1391, 1330, 1241, 1158, 1136, 1088, 1052, 1013, 975, 950, 890 cm^−1^; ^1^H-NMR (300 MHz, CDCl_3_) *δ* 7.76 (d, ^3^*J*_HH_ = 7.7 Hz, 2H, Ar*H*), 7.21 (d, ^3^*J*_HH_ = 7.9 Hz, 2H, Ar*H*), 5.62 (dd, ^3^*J*_HH_ = 9.5 Hz, ^3^*J*_PH_ = 5.3 Hz, 1H, N*H*), 4.18–3.94 (m, 5H, C*H*-P and OC*H*_2_), 3.50–3.20 (m, 4H, OC*H*_2_), 2.35 (s, 3H, C*H*_3_), 1.43 (s, 3H, C*H*_3_), 1.25–1.19 (m, 6H, C*H*_3_), 1.10 (t, ^3^*J*_HH_ = 7.1 Hz, 3H, C*H*_3_), 0.88 (t, ^3^*J*_HH_ = 7.1 Hz, 3H, C*H*_3_) ppm; ^13^C {1H}-NMR (75 MHz, CDCl_3_) *δ* 142.6, 139.5, 129.0, 126.8, 120.3 (C_Ar_), 100.9 (d, ^2^*J*_PC_ = 11.7 Hz, C_quat_), 62.7 (d, ^2^*J*_PC_ = 7.1 Hz, OCH_2_), 62.7 (d, ^2^*J*_PC_ = 6.6 Hz, OCH_2_), 56.9 (OCH_2_), 55.1 (d, ^1^*J*_PC_ = 153.6 Hz, CH-P), 56.0 (OCH_2_), 21.4 (CH_3_), 19.7 (CH_3_), 16.3 (d, ^3^*J*_PC_ = 3.1 Hz, CH_3_), 16.2 (d, ^3^*J*_PC_ = 3.0 Hz, CH_3_), 15.1 (CH_3_), 14.7 (CH_3_) ppm; ^31^P-NMR (120 MHz, CDCl_3_) *δ* 20.8 ppm; ESI-HRMS (CI) *m/z* calcd. for C_18_H_32_NNaO_7_PS ([M + Na]^+^) 460.1535, found 460.1549.


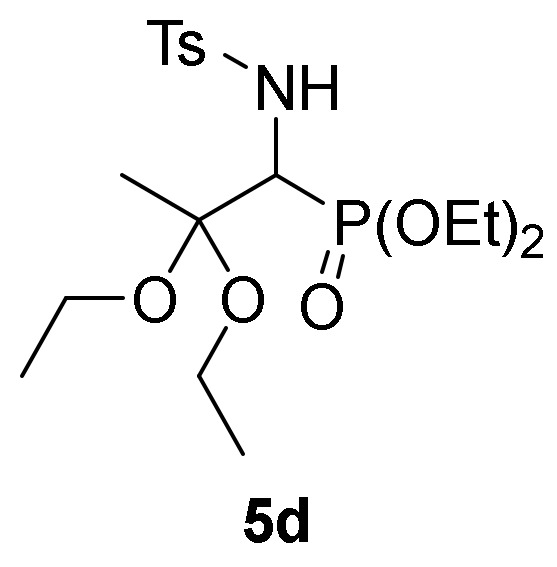


##### General Procedure and Spectral Data of β-Keto-α-Aminophosphine Oxide **6**

To a stirred solution of α-aminophosphonate acetal **5b** (0.87 g, 2 mmol) in CHCl_3_ (10 mL), a 37% solution of HCl (5 drops) was added dropwise. The mixture was refluxed for 5 h and was allowed to reach 25 °C. The crude product was washed twice with water (5 mL). The organic layer was dried over anhydrous MgSO_4_, filtered and concentrated to dryness in vacuum, and the resulting residue was purified by crystallization from Et_2_O/hexane 50:50 to afford the title compound **6**.

*Diisopropyl [1-((4-methylphenyl)sulfonamido)-2-oxopropyl]phosphonate* (**6**), (0.53 g, 68%) as a white solid. mp 121–122 °C; IR (neat) *v*_max_ 3136, 2977, 1717, 1328, 1230, 995 cm^−1^; ^1^H-NMR (400 MHz, CDCl_3_) *δ* 7.67 (d, ^3^*J*_HH_ = 8.3 Hz, 2H, Ar*H*), 7.26 (d, ^3^*J*_HH_ = 8.0 Hz, 2H, Ar*H*), 5.55 (dd, ^3^*J*_HH_ = 9.3 Hz, ^3^*J*_PH_ = 2.0 Hz, 1H, N*H*), 4.79–4.71 (m, 2H, OC*H*), 4.70–4.62 (m, 2H, OC*H*), 4.41 (dd, ^3^*J*_HH_ = 9.3 Hz, ^2^*J*_PH_ = 25.2 Hz, 1H, C*H*-P), 2.39 (s, 3H, C*H*_3_), 2.14 (s, 3H, C*H*_3_), 1.34–1.25 (m, 12H, CH(C*H*_3_)_2_) ppm; ^13^C {1H}-NMR (100 MHz, CDCl_3_) *δ* 199.8 (C = O), 144.2, 136.0, 129.8, 127.4 (C_Ar_), 73.4 (d, ^2^*J*_PC_ = 7.2 Hz, OCH), 73.3 (d, ^2^*J*_PC_ = 7.2 Hz, OCH), 61.5 (d, ^1^*J*_PC_ = 143.2 Hz, CH-P), 28.8 (CH_3_), 24.0 (d, ^3^*J*_PC_ = 3.6 Hz, CH_3_), 23.9 (d, ^3^*J*_PC_ = 3.8 Hz, CH_3_), 23.7 (d, ^3^*J*_PC_ = 5.2 Hz, CH_3_), 23.6 (d, ^3^*J*_PC_ = 5.4 Hz, CH_3_), 21.6 (CH_3_) ppm; ^31^P-NMR (120 MHz, CDCl_3_) *δ* 11.8 ppm; ESI-HRMS (CI) *m/z* calcd. for C_16_H_27_NO_6_PS ([M + H]^+^) 392.1297, found 392.1293.


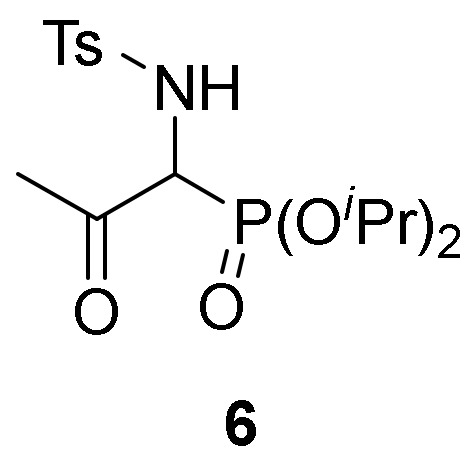


#### 3.1.3. Experimental Procedure and Characterization Data for Compounds **7**, **8** and **9**

##### General procedure and spectral data for the addition of 2,2,2-trifluoroethanol (2c) to functionalized 2H-azirines **1**

To a 0 °C solution of 2*H*-azirine **1a** (1.28 g, 5 mmol, 1 eq) in CH_2_Cl_2_ (25 mL) was added dropwise 2,2,2-trifluoroethanol (**2c**) (0.73 mL, 10 mmol, 2 eq), Et_3_N (3.15 mL, 22.5 mmol, 4.5 eq), and 4 Å M.S. The reaction mixture was allowed to reach 25 °C and stirred at the same temperature for 24 h. 4 Å M.S. was then filtered through a sintered glass vacuum filtration funnel with celite and washed with CH_2_Cl_2_. The reaction mixture was washed with water (3 × 15 mL) and extracted with CH_2_Cl_2_ (15 mL). The organic layers were dried over anhydrous MgSO_4_, filtered and concentrated to dryness in vacuum. The crude product was purified by crystallization in Et_2_O.

[(2S*,3S*)-3-Methyl-3-(2,2,2-trifluoroethoxy)aziridin-2-yl]diphenylphosphine oxide (**7**), (1.62 g, 91%) as a yellow solid. mp 98–100 °C; IR (neat) *v*_max_ 3439, 3248, 2972, 2941, 1635, 1590, 1438, 1394, 1359, 1280, 1252, 1169, 1122, 1080, 745, 726, 694 cm^−1^; ^1^H-NMR (300 MHz, CDCl_3_) *δ* 7.78–7.38 (m, 10H, Ar*H*), 3.88 (dq, ^2^*J*_HH_ = 1.5 Hz, ^3^*J*_HF_ = 8.7 Hz, 2H, C*H*_2_), 2.53 (dd, ^3^*J*_HH_ = 9.4 Hz, ^2^*J*_PH_ = 21.5 Hz, 1H, C*H*-P), 1.84 (dd, ^3^*J*_HH_ = 9.7 Hz, ^3^*J*_PH_ = 18.0 Hz, 1H, N*H*), 1.69 (s, 3H, C*H*_3_) ppm; ^13^C {1H}-NMR (75 MHz, CDCl_3_) *δ* 132.5, 132.4, 132.3, 132.2, 131.2, 130.9, 130.9, 130.8, 130.7, 129.0, 128.8, 128.7 (C_Ar_), 125.4 (d, ^1^*J*_CF_ = 277.8 Hz, CF_3_), 71.6 (C_quat_), 61.8 (q, ^2^*J*_CF_ = 33.8 Hz, CH_2_), 38.0 (d, ^1^*J*_PC_ = 89.9 Hz), 17.0 (CH_3_) ppm; ^31^P-NMR (120 MHz, CDCl_3_) *δ* 25.2 ppm; ^19^F NMR (282 MHz, CDCl_3_) *δ* –74.6, –74.7, –74.7 ppm; ESI-HRMS (CI) *m/z* calcd. for C_17_H_18_F_3_NO_2_P ([M + H]^+^) 356.1027, found 356.1014.


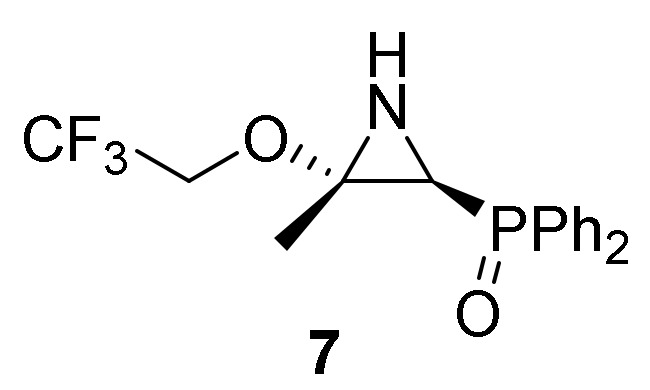


##### General Procedure and Spectral Data of Allylic α-Aminophosphine Oxide **8**

A solution of aziridine **7** (1.78 g, 5 mmol, 1 eq) was stirred in refluxing CHCl_3_ (11mL) for 15 h until TLC showed the disappearance of aziridine **7**. The crude product was purified by flash-column chromatography (SiO_2_, AcOEt/hexane 50:50) to afford the title compound **8**.

*[1-Amino-2-(2,2,2-trifluoroethoxy)allyl]diphenylphosphine oxide* (**8**), (1.30 g, 73%) as a yellow oil. IR (neat) *v*_max_ 3387, 3314, 3059, 2940, 1638, 1591, 1438, 1288, 1169, 1119, 1102, 975, 910, 827, 730, 694 cm^−1^; ^1^H-NMR (300 MHz, CDCl_3_) *δ* 7.90–7.37 (m, 10H, Ar*H*), 4.45 (t, ^2^*J*_HH_ = 3.5 Hz, 1H, = C*H_2_*), 4.20 (d, ^2^*J*_PH_ = 8.7 Hz, 1H, C*H*-P), 4.10 (t, ^2^*J*_HH_ = 3.2 Hz, 1H, = C*H_2_*), 3.92–3.80 (m, 1H, C*H_2_*), 3.64–3.52 (m, 1H, C*H_2_*), 1.95 (bs, 2H, N*H*_2_) ppm; ^13^C {1H}-NMR (75 MHz, CDCl_3_) *δ* 158.6 (d, ^2^*J*_PC_ = 1.9 Hz, = C-O), 132.0, 132.0, 131.9, 131.9, 131.6, 131.5, 131.5, 131.4 (C_Ar_), 122.7 (q, ^1^*J*_CF_ = 277.6 Hz, CF_3_), 86.8 (d, ^3^*J*_PC_ = 6.2 Hz, = CH_2_), 64.5 (q, ^2^*J*_CF_ = 36.2 Hz, CH_2_), 55.4 (d, ^1^*J*_PC_ = 71.9 Hz) ppm; ^31^P-NMR (120 MHz, CDCl_3_) *δ* 30.2 ppm; ^19^F NMR (282 MHz, CDCl_3_) *δ* –73.9, –74.0, –74.0 ppm; ESI-HRMS (CI) *m/z* calcd. for C_17_H_17_F_3_NNaO_2_P ([M + Na]^+^) 378.0847, found 378.0851.


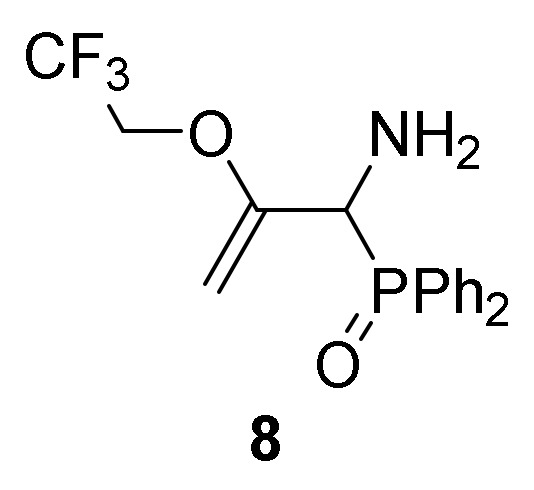


##### General Procedure and Spectral Data of Allylic N-Tosyl α-Aminophosphine Oxide **9**

*p*-Toluenesulfonyl chloride (1 g, 5.5 mmol, 1.1 eq) and pyridine (2.4 mL, 30 mmol, 6 eq) were added to a 0 °C solution of **8** (1.78 g, 5 mmol, 1 eq) in CH_2_Cl_2_ (25 mL). The reaction mixture was allowed to reach 25 °C and stirred for 24 h. The crude product was washed twice with a 2M HCl solution (15 mL) and water (15 mL) and extracted with CH_2_Cl_2_ (15 mL). The organic layer was dried over anhydrous MgSO_4_, filtered and concentrated to dryness in vacuum. The crude product was purified by crystallization from Et_2_O to afford the title compound **9**.

*N-[1-(Diphenylphosphoryl)-2-(2,2,2-trifluoroethoxy)allyl]-4-methylbenzenesulfonamide* (**9**), (2.29 g, 90%) obtained as a pale yellow solid. mp 201–203 °C; IR (neat) *v*_max_ 3412, 3062, 2942, 2879, 1652, 1596, 1444, 1338, 1285, 1160, 910, 733 cm^−1^; ^1^H-NMR (400 MHz, CDCl_3_) *δ* 7.85–7.72 (m, 4H, Ar*H*), 7.67 (d, ^3^*J*_HH_ = 8.3 Hz, 2H, Ar*H*), 7.56–7.38 (m, 6H, Ar*H*), 7.11 (d, ^3^*J*_HH_ = 8.4 Hz, 2H, Ar*H*), 7.07 (d, ^3^*J*_HH_ = 9.8 Hz, 1H, N*H*), 4.80 (t, ^2^*J*_PH_ = 11.1 Hz, 1H, C*H*-P), 4.48 (t, ^2^*J*_HH_ = 3.8 Hz, 1H, = C*H_2_*), 3.67 (t, ^2^*J*_HH_ = 3.6 Hz, 1H, = C*H_2_*), 3.42–3.33 (m, 1H, C*H_2_*), 3.00–2.91 (m, 1H, C*H_2_*), 2.34 (s, 3H, C*H*_3_) ppm; ^13^C {1H}-NMR (100 MHz, CDCl_3_) *δ* 153.4, 143.2, 137.5, 132.4, 132.3, 131.6, 131.5, 130.0, 129.8, 129.0, 128.8, 128.7, 128.4, 128.2, 127.6 (C_Ar_), 122.5 (q, ^1^*J*_CF_ = 277.5 Hz, CF_3_), 89.0 (d, ^3^*J*_PC_ = 6.1 Hz, = CH_2_), 64.2 (q, ^2^*J*_CF_ = 35.6 Hz, CH_2_), 55.5 (d, ^1^*J*_PC_ = 73.5 Hz, CH-P), 21.5 (CH_3_) ppm; ^31^P-NMR (120 MHz, CDCl_3_) *δ* 29.6 ppm; ^19^F NMR (282 MHz, CDCl_3_) *δ* –73.9, –73.9, –73.9 ppm; ESI-HRMS (CI) *m/z* calcd. for C_24_H_24_F_3_NO_4_PS ([M + H]^+^) 510.1116, found 510.1117.


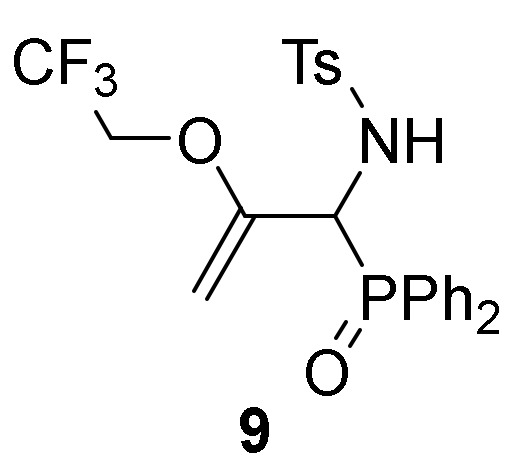


#### 3.1.4. Experimental Procedure and Characterization Data for Compounds **10**, **11** and **12**

##### General Procedure and Spectral Data for the Addition of Phenols (**2d**–**e**) to Functionalized 2H-Azirines **1**

To a 0 °C solution of 2*H*-azirine **1** (5 mmol, 1 eq) in CH_2_Cl_2_ (25 mL), the corresponding phenols (**2d–e**) (10 mmol, 2 eq) and Et_3_N (1.4 mL, 10 mmol, 2 eq) was added dropwise. The reaction mixture was allowed to reach 25 °C and stirred at the same temperature for 24 h. The reaction mixture was washed with water (3 × 15 mL) and extracted with CH_2_Cl_2_ (15 mL). The organic layers was dried over anhydrous MgSO_4_, filtered and concentrated to dryness in vacuum. The crude products were purified by crystallization or by flash-column chromatography.

[(2S*,3S*)-3-Methyl-3-phenoxyaziridin-2-yl]diphenylphosphine oxide (**10a**), (1.22 g, 70%) obtained as a pale yellow solid from 2*H*-azirine **1a** (1.28 g, 5 mmol) and phenol (**2d**) (0.88 g, 10 mmol) as described in the general procedure. The crude product was purified by crystallization from Et_2_O to afford the title compound **10a**. mp 124–126 °C; IR (neat) *v*_max_ 3203, 3059, 2990, 1593, 1488, 1438, 1391, 1349, 1224, 1191, 1122, 733, 691 cm^−1^; ^1^H-NMR (300 MHz, CDCl_3_) *δ* 7.85–7.42 (m, 10H, Ar*H*), 7.07–6.63 (m, 5H, Ar*H*), 2.62 (dd, ^3^*J*_HH_ = 10.0 Hz, ^2^*J*_PH_ = 22.0 Hz, 1H, C*H*-P), 2.06 (dd, ^3^*J*_HH_ = 10.2 Hz, ^3^*J*_PH_ = 18.2 Hz, 1H, N*H*), 1.87 (s, 3H, C*H*_3_) ppm; ^13^C {1H}-NMR (75 MHz, CDCl_3_) *δ* 154.9 (OC_Ar_), 132.5, 132.4, 132.2, 131.2, 131.1, 131.0, 130.9, 129.2, 129.0, 128.8, 128.7, 121.7, 116.7 (C_Ar_), 70.3 (C_quat_), 37.9 (d, ^1^*J*_PC_ = 88.7 Hz, CH-P), 16.9 (CH_3_) ppm; ^31^P-NMR (120 MHz, CDCl_3_) *δ* 25.1 ppm; ESI-HRMS (CI) *m/z* calcd. for C_21_H_21_NO_2_P ([M + H]^+^) 350.1310, found 350.1302.


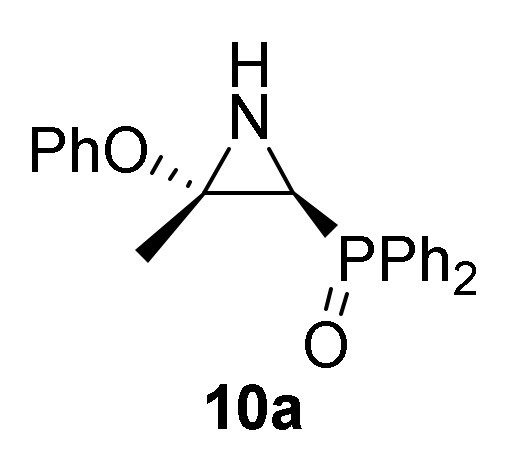


[(2S*,3S*)-3-Methyl-3-(naphthalen-2-yloxy)aziridin-2-yl]diphenyl-phosphine oxide (**10b**), Obtained as a pale yellow solid from 2*H*-azirine **1a** (1.28 g, 5 mmol) and 2-naphthol (**2e**) (1.44 g, 10 mmol) as described in the general procedure. The crude product was purified by crystallization from Et_2_O to afford title compound **10b**. This product was identified only by ^1^H-NMR, since cleavage of C3–N bond in the three-membered ring of **10b** promptly occurs to give a mixture of aziridine **10b** and allyl α-aminophosphine oxide **11b**. ^1^H-NMR (300 MHz, CDCl_3_) *δ* 7.92–6.83 (m, 17H, Ar*H*), 2.77 (d, ^2^*J*_PH_ = 20.0 Hz, 1H, C*H*-P), 2.23 (bs, 1H, N*H*), 2.02 (s, 3H, C*H*_3_) ppm.


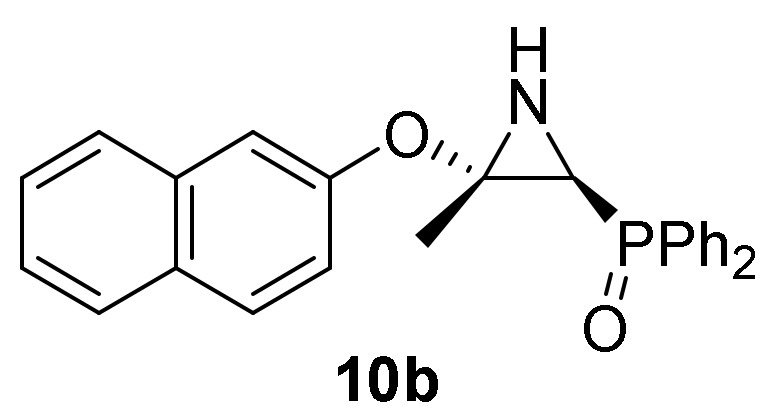


##### General Procedure for the Preparation of Allylic α-Aminophosphine Oxides **11**

A solution of aziridine **10** (5 mmol, 1 eq) was stirred in refluxing CHCl_3_ (11mL) for 8 h until TLC showed the disappearance of aziridine **10**. The crude product was concentrated to dryness in vacuum to afford the title compound **11**.

*(1-Amino-2-phenoxyallyl)diphenylphosphine oxide* (**11a**), (1.62 g, 93%) obtained as an orange oil from aziridine **10a** (1.75 g, 5 mmol) as described in the general procedure. IR (neat) *v*_max_ 3389, 3060, 2933, 2860, 1638, 1587, 1489, 1442, 1264, 1220, 1182, 1122, 910, 742 cm^−1^; ^1^H-NMR (300 MHz, CDCl_3_) *δ* 7.91–6.55 (m, 15H, Ar*H*), 4.36 (t, *J* = 2.8 Hz, 1H, = C*H_2_*), 4.30 (d, ^2^*J*_PH_ = 8.6 Hz, 1H, C*H*-P), 3.94 (t, *J* = 2.3 Hz, 1H, = C*H_2_*), 2.21 (bs, 2H, N*H*_2_) ppm; ^13^C {1H}-NMR (75 MHz, CDCl_3_) *δ* 159.8 (d, ^2^*J*_PC_ = 2.6 Hz, = C_quat_), 153.8 (OC_Ar_), 131.5, 131.5, 131.4, 131.4, 131.3, 131.2, 130.3, 130.0, 129.1, 128.2, 128.0, 128.0, 127.8, 124.1, 120.5, 116.2, 115.4 (C_Ar_), 90.5 (d, ^3^*J*_PC_ = 6.6 Hz, = CH_2_), 55.1 (d, ^1^*J*_PC_ = 73.1 Hz, CH-P) ppm; ^31^P-NMR (120 MHz, CDCl_3_) *δ* 30.2 ppm; ESI-HRMS (CI) *m/z* calcd. for C_21_H_20_NNaO_2_P ([M + Na]^+^) 372.1129, found 372.1134.


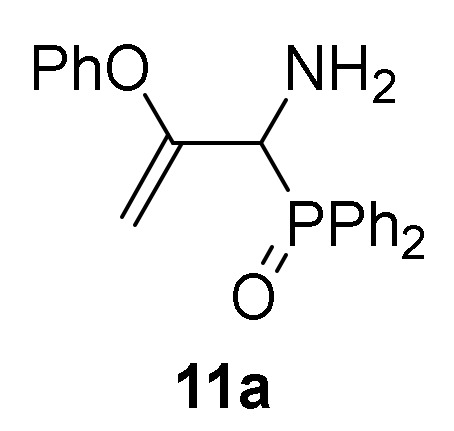


*[1-Amino-2-(naphyhalen-2-yloxy)allyl]diphenylphosphine oxide* (**11b**), (1.48 g, 74%) obtained as an orange oil from aziridine **10b** (2.00 gr, 5mmol) as described in the general procedure. IR (neat) *v*_max_ 3382, 3069, 2923, 1635, 1597, 1508, 1438, 1249, 1211, 1179, 1125, 907, 745, 698 cm^−1^; ^1^H-NMR (300 MHz, CDCl_3_) *δ* 8.04–6.53 (m, 17H, Ar*H*), 4.55 (t, *J* = 2.9 Hz, 1H, = C*H_2_*), 4.46 (d, ^2^*J*_PH_ = 8.4 Hz, 1H, C*H*-P), 4.14 (t, *J* = 2.5 Hz, 1H, = C*H_2_*), 2.11 (bs, 2H, N*H*_2_) ppm; ^13^C {1H}-NMR (75 MHz, CDCl_3_) *δ* 160.0 (d, ^2^*J*_PC_ = 2.6 Hz, = C_quat_), 151.7 (OC_Ar_), 133.9, 131.9, 131.8, 131.7, 131.6, 130.6, 129.4, 128.5, 128.4, 128.3, 128.2, 127.5, 127.1, 126.2, 125.1, 120.9, 117.1, 111.6, 109.4 (C_Ar_), 91.5 (d, ^3^*J*_PC_ = 6.6 Hz, = CH_2_), 55.5 (d, ^1^*J*_PC_ = 72.3 Hz, CH-P) ppm; ^31^P-NMR (120 MHz, CDCl_3_) *δ* 30.5 ppm; ESI-HRMS (CI) *m/z* calcd. for C_25_H_22_NNaO_2_P ([M + Na]^+^) 422.1286, found 422.1291.


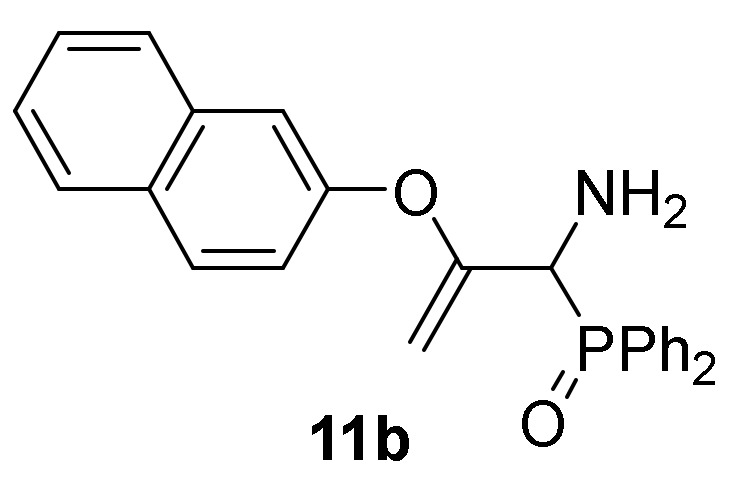


##### One Pot Procedure for the Synthesis of N-Tosyl Allyl Amines 12 Derived From Phosphonate

To a 0 °C solution of 2*H*-azirine **1e** (0.96 g, 5 mmol) in CH_2_Cl_2_ (25 mL), the corresponding alcohol (**2d**–**e**) (10 mmol, 2 eq) and Et_3_N (1.4 mL, 10 mmol, 2 eq) was added dropwise. The reaction mixture was allowed to reach 25 °C and stirred for 24 h until TLC showed the disappearance of starting 2*H*-azirine **1e**. The reaction mixture was washed with water (3 × 15 mL) and extracted with CH_2_Cl_2_. The organic layer was dried over anhydrous MgSO_4_, filtered and concentrated to dryness in vacuum. Without any further purification step, to a 0 °C solution of crude products **11** in CH_2_Cl_2_ (25 mL) was directly added *p*-toluenesulfonyl chloride (1 g, 5.5 mmol, 1.1 eq) and pyridine (2.42 mL, 30 mmol, 6 eq). The reaction mixture was allowed to reach 25 °C and stirred for 24 h. The crude product was washed twice with a 2M HCl solution (15 mL) and water (15 mL) and extracted with CH_2_Cl_2_ (15 mL). The organic layer was dried over anhydrous MgSO_4_, filtered and concentrated to dryness in vacuum. The crude products **12** were purified by crystallization from Et_2_O.

*Diethyl [1-((4-methylphenyl)sulfonamido)-2-phenoxyallyl]phosphonate* (**12a**), (1.93 g, 88%) obtained as a white solid from phenol (**2d**) (0.88 g, 10 mmol) following the general procedure described above. mp 117–119 °C; IR (neat) *v*_max_ 3270, 3123, 2984, 2934, 2915, 2881, 1643, 1593, 1494, 1452, 1391, 1344, 1241, 1091, 1044, 1013, 972 cm^−1^; ^1^H-NMR (400 MHz, CDCl_3_) *δ* 7.79 (d, ^3^*J*_HH_ = 8.3 Hz, 2H, Ar*H*), 7.25–7.04 (m, 5H, Ar*H*), 6.61 (d, ^3^*J*_HH_ = 8.5 Hz, 2H, Ar*H*), 6.09 (dd, ^3^*J*_HH_ = 10.1 Hz, ^3^*J*_PH_ = 3.1 Hz, 1H, N*H*), 4.44 (dd, ^3^*J*_HH_ = 10.1 Hz, ^2^*J*_PH_ = 23.9 Hz, 1H, C*H*-P), 4.31 (t, *J* = 3.2 Hz, 1H, = C*H*_2_), 4.24–4.09 (m, 4H, OC*H*_2_), 3.83 (t, *J* = 2.5 Hz, 1H, = C*H*_2_), 2.40 (s, 3H, C*H*_3_), 1.29 (q, ^3^*J*_HH_ = 7.0 Hz, 3H, C*H*_3_), 1.28 (q, ^3^*J*_HH_ = 7.0 Hz, 3H, C*H*_3_) ppm; ^13^C {1H}-NMR (100 MHz, CDCl_3_) *δ* 155.1 (d, ^2^*J*_PC_ = 2.7 Hz, = C_quat_), 154.0 (OC_Ar_), 143.4 (C_quat_Ar), 137.9 (d, ^4^*J*_PC_ = 1.6 Hz, C_quat_Ar), 129.4, 129.4, 127.5, 124.7, 121.0 (C_Ar_), 91.6 (d, ^3^*J*_PC_ = 8.8 Hz, = CH_2_), 63.8 (d, ^2^*J*_PC_ = 6.7 Hz, OCH_2_), 63.7 (d, ^2^*J*_PC_ = 6.9 Hz, OCH_2_), 54.1 (d, ^1^*J*_PC_ = 157.4 Hz, CH-P), 21.4 (CH_3_), 16.3 (d, ^3^*J*_PC_ = 6.1 Hz, CH_3_), 16.3 (d, ^3^*J*_PC_ = 6.2 Hz, CH_3_) ppm; ^31^P-NMR (120 MHz, CDCl_3_) *δ* 18.0 ppm; ESI-HRMS (CI) *m/z* calcd. for C_20_H_26_NO_6_PS ([M + H]^+^) 440.1297, found 440.1304.


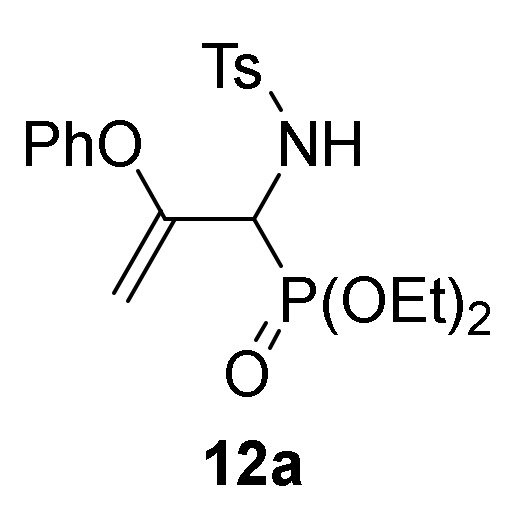


*Diethyl [1-((4-methylphenyl)sulfonamido)-2-(naphthalen-2-yloxy)allyl]-phosphonate* (**12b**), (1.64 g, 67%) obtained as a grey solid from 2-naphthol (**2e**) (1.44 g, 10 mmol) following the general procedure described above. mp 147–149 °C; IR (neat) *v*_max_ 3425, 3065, 2981, 2926, 1599, 1380, 1191, 1177, 816, 714, 664 cm^−1^; ^1^H-NMR (300 MHz, CDCl_3_) *δ* 7.87 (d, ^3^*J*_HH_ = 8.1 Hz, 2H, Ar*H*), 7.81–7.39 (m, 5H, Ar*H*), 7.31 (d, ^3^*J*_HH_ = 8.2 Hz, 2H, Ar*H*), 7.07 (d, *J* = 1.8 Hz, 1H, Ar*H*), 6.85 (dd, *J* = 2.2 Hz, *J* = 8.9 Hz, 1H, Ar*H*), 6.17 (dd, ^3^*J*_HH_ = 10.0 Hz, ^3^*J*_PH_ = 3.3 Hz, 1H, N*H*), 4.54 (dd, ^3^*J*_HH_ = 10.1 Hz, ^2^*J*_PH_ = 23.8 Hz, 1H, C*H*-P), 4.41 (t, *J* = 3.1 Hz, 1H, = C*H*_2_), 4.33–4.16 (m, 4H, OC*H*_2_), 3.93 (t, *J* = 2.6 Hz, 1H, = C*H*_2_), 2.46 (s, 3H, C*H*_3_), 1.35 (q, ^3^*J*_HH_ = 7.2 Hz, 6H, C*H*_3_) ppm; ^13^C {1H}-NMR (75 MHz, CDCl_3_) *δ* 155.1 (d, ^2^*J*_PC_ = 2.9 Hz, = C_quat_), 151.5 (OC_Ar_), 143.5 (C_quat_Ar), 138.0 (C_quat_Ar), 133.9 (C_quat_Ar), 130.9 (C_quat_Ar), 129.5, 129.4, 127.7, 127.6, 127.2, 126.4, 125.3, 120.9, 117.6 (C_Ar_), 92.1 (d, ^3^*J*_PC_ = 9.1 Hz, = CH_2_), 63.9 (d, ^2^*J*_PC_ = 6.7 Hz, OCH_2_), 63.8 (d, ^2^*J*_PC_ = 7.1 Hz, OCH_2_), 54.2 (d, ^1^*J*_PC_ = 157.5 Hz, CH-P), 21.5 (CH_3_), 16.4 (d, ^3^*J*_PC_ = 5.6 Hz, CH_3_) ppm; ^31^P-NMR (120 MHz, CDCl_3_) *δ* 18.1 ppm; ESI-HRMS (CI) *m/z* calcd. for C_24_H_29_NO_6_PS ([M + H]^+^) 490.1453, found 490.1469.


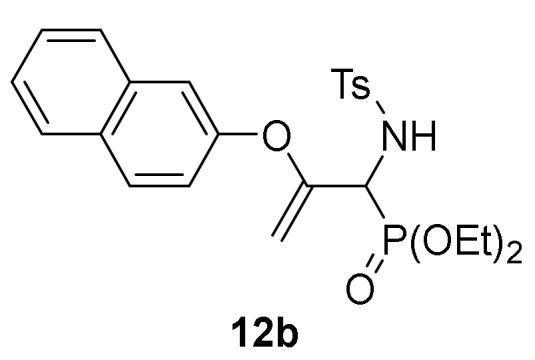


#### 3.1.5. Experimental Procedure and Characterization Data for Compounds **14**, **15** and **16**

##### General Procedure and Spectral Data for the Addition of Thiophenols and Thiols to 2H-Azirines **1**

*Method A*: To a 0 °C solution of 2*H*-azirine **1** (5 mmol, 1 eq) in CH_2_Cl_2_ (25 mL) was added dropwise thiophenol or thiol (5.5 mmol, 1.1 eq), Et_3_N (1.40 mL, 10 mmol, 2 eq), and 4 Å M.S. The reaction mixture was allowed to reach 25 °C and stirred at the same temperature for 24 h. 4 Å M.S. was then filtered through a sintered glass vacuum filtration funnel with celite and washed with CH_2_Cl_2_. The reaction mixture was washed with water (3 × 15 mL) and extracted with CH_2_Cl_2_ (15 mL). The organic layers were dried over anhydrous MgSO_4_, filtered and concentrated to dryness in vacuum to give aziridine **14**. In the case of R^1^ = Me, aziridines **14**, allylic α-aminophosphine oxides or phosphonates **15**, or a mixture of both compounds can be obtained. When aziridines **14** or the mixture is obtained in the reaction crude, stirring of this crude in refluxing CHCl_3_ afford allylic α-aminophosphine oxides or phosphonates **15**. The crude products **14** or **15** were purified by crystallization or by flash-column chromatography.

*Method B*: To a 0 °C solution of 2*H*-azirine **1** (5 mmol, 1 eq) in CH_2_Cl_2_ (25 mL) was added dropwise the corresponding *p*-substituted benzenethiol (5.5 mmol, 1.1 eq). The reaction mixture was stirred at 0 °C for 48 h until TLC showed the disappearance of starting compound **1**. The reaction mixture was concentrated to dryness in vacuum to afford allylic α-aminophosphine oxides or phosphonates **15**.

[(2S*,3S*)-3-Methyl-3-(phenylthio)aziridin-2-yl]diphenylphosphine oxide (**14a**), (1.68 g, 92%) as a white solid from 2*H*-azirine **1a** (1.28 g, 5 mmol) and benzenethiol (**13a**) (0.56 mL, 5.5 mmol) as described in the general procedure (method A). The crude product was purified by crystallization from Et_2_O to afford the title compound **14a**. This product was identified only by ^1^H- and ^31^P-NMR, since cleavage of C3–N bond in three-membered ring of **14a** promptly occurs to give allylic α-aminophosphine oxide **15a**. ^1^H-NMR (400 MHz, CDCl_3_) *δ* 7.68–7.21 (m, 15H, Ar*H*), 2.60 (dd, ^3^*J*_HH_ = 8.4 Hz, ^2^*J*_PH_ = 23.2 Hz, 1H, C*H*-P), 1.95 (dd, ^3^*J*_HH_ = 8.8 Hz, ^3^*J*_PH_ = 15.0 Hz, 1H, N*H*), 1.76 (s, 3H, C*H*_3_) ppm; ^31^P-NMR (120 MHz, CDCl_3_) *δ* 26.8 ppm.


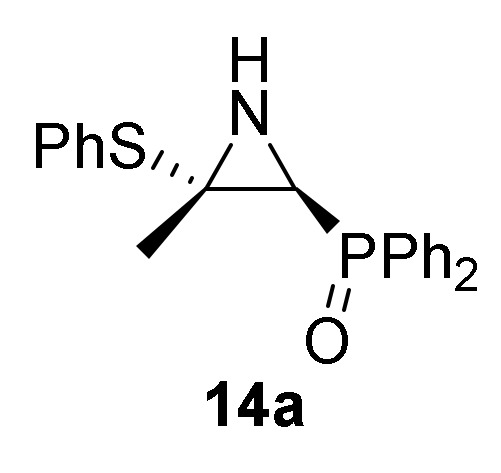


Diphenyl [(2S*,3S*)-3-methyl-3-(p-tolylthio)aziridin-2-yl]phosphine oxide (**14b**), From 2*H*-azirine **1a** (1.28 g, 5 mmol) and 4-methylbenzenethiol (**13b**) (0.68 g, 5.5 mmol) as described in the general procedure (method B). This product was identified only by ^1^H and ^31^P-NMR in a mixture of aziridine **14b** and allylic α-aminophosphine oxide **15b**, since cleavage of C3–N bond in three-membered ring of **14b** promptly occurs to give allylic α-aminophosphine oxide **15b**. ^1^H-NMR (300 MHz, CDCl_3_) *δ* 8.08–7.43 (m, 28H, Ar*H*)_mixture_, 2.78 (d, ^2^*J*_PH_ = 23.4 Hz, 1H, C*H*-P), 2.48 (s, 3H, C*H*_3_), 1.90 (s, 3H, C*H*_3_) ppm; ^31^P-NMR (120 MHz, CDCl_3_) *δ* 26.8 ppm.


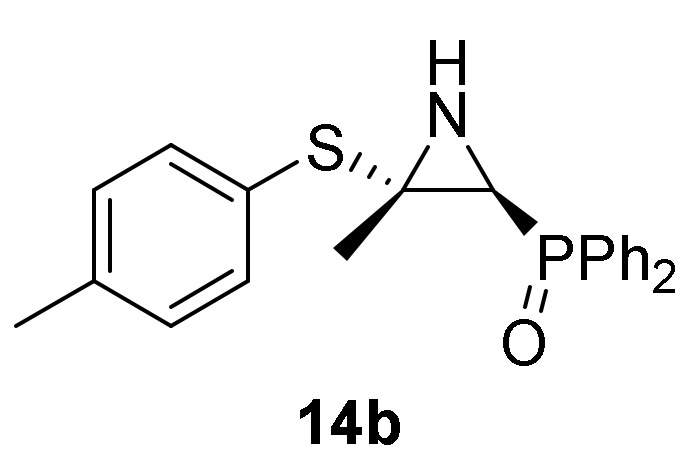


Diphenyl [(2S*,3S*)-3-phenyl-3-(phenylthio)aziridin-2-yl]phosphine oxide (**14c**), (1.29 g, 60%) as a white solid from 2*H*-azirine **1c** (1.59 g, 5 mmol) and benzenethiol (**13a**) (0.56 mL, 5.5 mmol) as described in the general procedure (method A). The crude product was purified by crystallization from Et_2_O/CH_2_Cl_2_ 50:50 to afford the title compound **14c**, whose data are in agreement with those reported previously [43].


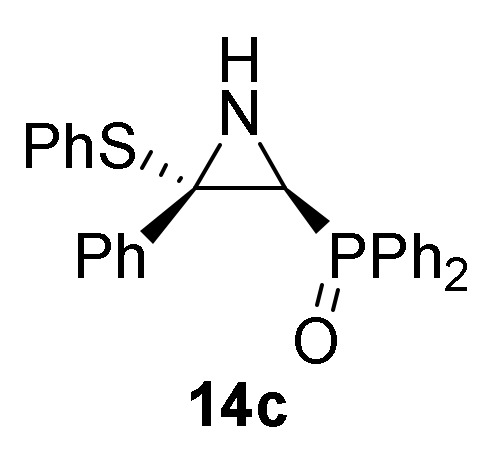


*[1-Amino-2-(phenylthio)allyl]diphenylphosphine oxide* (**15a**), Following the general procedure described above (method A), aziridine intermediate **14a** was stirred in refluxing CHCl_3_ (11mL) for 8 h. The crude product was concentrated to dryness in vacuum to afford the title compound **15a**, whose data are in agreement with those reported previously [43].


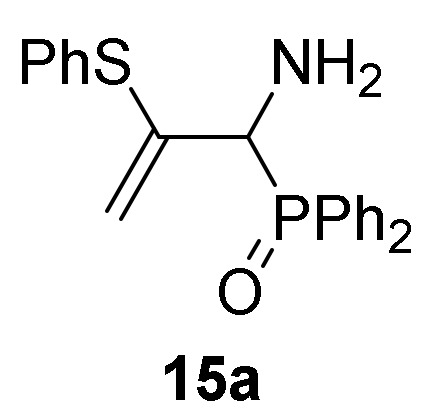


*[1-Amino-2-(p-tolylthio)allyl]diphenylphosphine oxide* (**15b**), (1.69 g, 89%) as a yellow oil from 2*H*-azirine **1a** (1.28 g, 5 mmol) and 4-methylbenzenethiol (**13b**) (0.68 g, 5.5 mmol) as described in the general procedure (method B). IR (neat) *v*_max_ 3389, 3060, 3022, 2987, 2930, 1676, 1635, 1590, 1489, 1442, 1242, 1185, 1119, 1106, 910, 729, 694 cm^−1^; ^1^H-NMR (400 MHz, CDCl_3_) *δ* 7.96–7.06 (m, 14H, Ar*H*), 5.56 (d, ^2^*J*_HH_ = 3.3 Hz, 1H, = C*H_2_*), 4.88 (d, ^2^*J*_HH_ = 3.2 Hz, 1H, = C*H_2_*), 4.13 (d, ^2^*J*_PH_ = 6.0 Hz, 1H, C*H*-P), 2.29 (s, 3H, C*H*_3_), 2.21 (bs, 2H, N*H_2_*) ppm; ^13^C {1H}-NMR (100 MHz, CDCl_3_) *δ* 144.2 ( = C_quat_), 138.4, 133.6, 131.8, 131.6, 131.5, 131.5, 131.4, 129.9, 129.6, 128.5, 128.4, 128.1, 127.9 (C_Ar_), 115.1 (d, ^3^*J*_PC_ = 6.6 Hz, = CH_2_), 55.8 (d, ^1^*J*_PC_ = 72.8 Hz, CH-P), 21.0 (CH_3_) ppm; ^31^P-NMR (120 MHz, CDCl_3_) *δ* 30.9 ppm; ESI-HRMS (CI) *m/z* calcd. for C_22_H_23_NOPS ([M + H]^+^) 380.1238, found 380.1225.


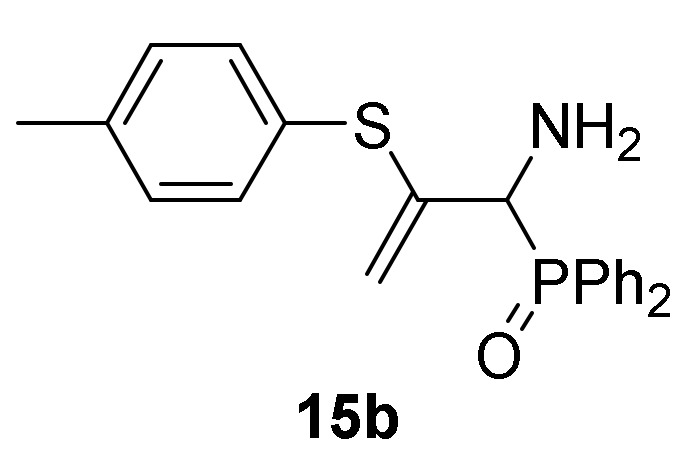


*[1-Amino-2-((4-fluorophenyl)thio)allyl]diphenylphosphine oxide* (**15c**), (1.46 g, 76%) as an orange oil from 2*H*-azirine **1a** (1.28 g, 5 mmol) and 4-fluorobenzenethiol (**13c**) (0.59 mL, 5.5 mmol) as described in the general procedure (method B). Rf = 0.15 (AcOEt); IR (neat) *v*_max_ 3381, 3301, 3065, 2914, 1593, 1495, 1437, 1226, 1185, 1157, 1122, 834, 726, 694 cm^−1^; ^1^H-NMR (300 MHz, CDCl_3_) *δ* 7.86–6.86 (m, 14H, Ar*H*), 5.50 (d, ^2^*J*_HH_ = 3.3 Hz, 1H, = C*H_2_*), 4.76 (d, ^2^*J*_HH_ = 3.1 Hz, 1H, = C*H_2_*), 4.11 (d, ^2^*J*_PH_ = 6.3 Hz, 1H, C*H*-P), 2.13 (bs, 2H, N*H*_2_) ppm; ^13^C {1H}-NMR (75 MHz, CDCl_3_) *δ* 164.2 (C_Ar_-F), 160.9 (C_quat_), 144.1 ( = C_quat_), 135.7, 135.6, 131.8, 131.7, 131.7, 131.6, 131.6, 131.5, 131.4, 131.4, 131.3, 130.2, 128.4, 128.3, 128.0, 127.9, 126.8, 126.7 (C_Ar_), 116.4, 116.1, 115.0 (d, ^3^*J*_PC_ = 6.8 Hz, = CH_2_), 55.9 (d, ^1^*J*_PC_ = 72.5 Hz, CH-P) ppm; ^31^P-NMR (120 MHz, CDCl_3_) *δ* 30.6 ppm; ^19^F NMR (282 MHz, CDCl_3_) *δ* –112.6 ppm; ESI-HRMS (CI) *m/z* calcd. for C_21_H_20_FNOPS ([M + H]^+^) 384.0987, found 384.0988.


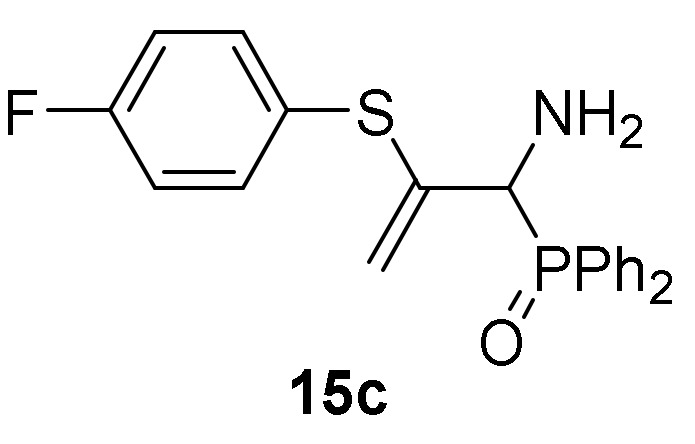


*[1-Amino-2-((4-methoxyphenyl)thio)allyl]diphenylphosphine oxide* (**15d**), (1.37 g, 70%) as a yellow oil from 2*H*-azirine **1a** (1.28 g, 5 mmol) and 4-methoxy-benzenethiol (**13d**) (0.77 g, 5.5 mmol) as described in the general procedure (method B). IR (neat) *v*_max_ 3381, 3053, 2965, 2940, 2837, 1596, 1574, 1491, 1463, 1438, 1288, 1247, 1180, 1113, 1102, 1030, 830, 725, 694 cm^−1^; ^1^H-NMR (400 MHz, CDCl_3_) *δ* 7.90–7.34 (m, 10H, Ar*H*), 7.09 (d, ^3^*J*_HH_ = 8.9 Hz, 2H, Ar*H*), 6.79 (d, ^3^*J*_HH_ = 8.8 Hz, 2H, Ar*H*), 5.48 (d, ^2^*J*_HH_ = 3.3 Hz, 1H, = C*H_2_*), 4.71 (d, ^2^*J*_HH_ = 2.9 Hz, 1H, = C*H_2_*), 4.11 (d, ^2^*J*_PH_ = 5.9 Hz, 1H, C*H*-P), 3.75 (s, 3H, OC*H*_3_), 1.95 (bs, 2H, N*H_2_*) ppm; ^13^C {1H}-NMR (100 MHz, CDCl_3_) *δ* 160.1 (C_Ar_-O), 145.3 ( = C_quat_), 136.0, 132.6, 131.9, 131.9, 131.7, 131.7, 131.6, 131.6, 131.5, 128.6, 128.5, 128.1, 128.0, 121.7, 114.8 (C_Ar_), 113.3 (d, ^3^*J*_PC_ = 6.7 Hz, = CH_2_), 55.8 (d, ^1^*J*_PC_ = 72.8 Hz, CH-P), 55.3 (OCH_3_)ppm; ^31^P-NMR (120 MHz, CDCl_3_) *δ* 30.9 ppm; ESI-HRMS (CI) *m/z* calcd. for C_22_H_23_NO_2_PS ([M + H]^+^) 396.1187, found 396.1183.


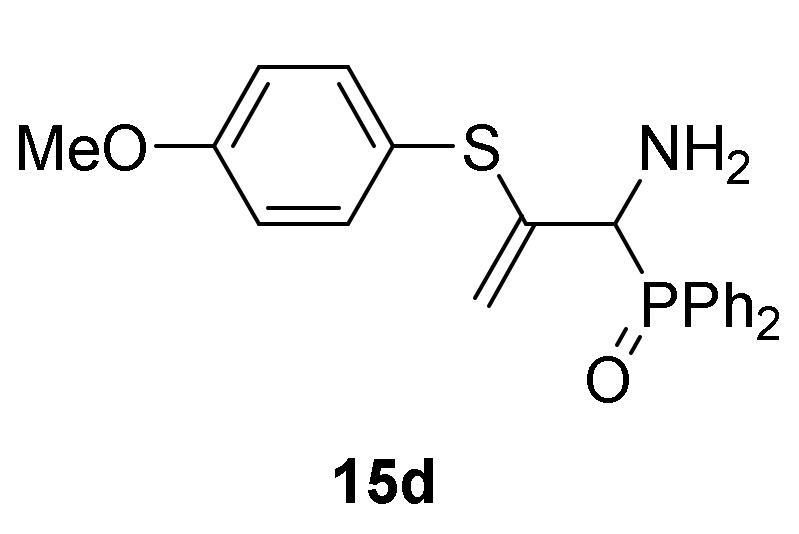


*Diethyl [1-amino-2-(phenylthio)allyl]phosphonate* (**15e**), (0.62 g, 41%) as a yellow oil from 2*H*-azirine **1e** (0.96 g, 5 mmol) and benzenethiol (**13a**) (0.56 mL, 5.5 mmol) as described in the general procedure (method B). The crude product was purified by flash-column chromatography (SiO_2_, AcOEt) to afford the title compound **15e**, whose data are in agreement with those reported previously [43].


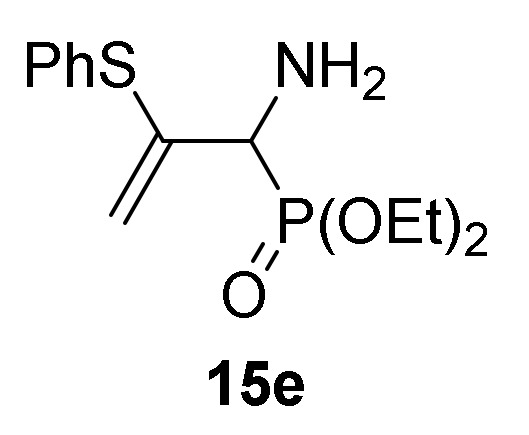


*[1-Amino-2-(ethylthio)allyl]diphenylphosphine oxide* (**15f**), (1.02 g, 64%) as a pale yellow oil from 2*H*-azirine **1a** (1.28 g, 5 mmol) and ethanethiol (**13e**) (0.40 mL, 5.5 mmol) as described in the general procedure (method A). The crude product was purified by flash-column chromatography (SiO_2_, AcOEt/methanol 95:5) to afford the title compound **15f**. IR (neat) *v*_max_ 3414, 3060, 2977, 1682, 1631, 1590, 1435, 1246, 1188, 1122, 739, 698 cm^−1^; ^1^H-NMR (300 MHz, CDCl_3_) *δ* 7.91–7.383 (m, 10H, Ar*H*), 5.46 (d, ^2^*J*_HH_ = 3.3 Hz, 1H, = C*H_2_*), 4.84 (d, ^2^*J*_HH_ = 2.4 Hz, 1H, = C*H_2_*), 4.07 (d, ^2^*J*_PH_ = 6.1 Hz, 1H, C*H*-P), 2.57 (q, ^3^*J*_HH_ = 7.4 Hz, 2H, C*H_2_*), 2.26 (bs, 2H, N*H_2_*), 1.09 (q, ^3^*J*_HH_ = 7.4 Hz, 3H, C*H*_3_) ppm; ^13^C {1H}-NMR (75 MHz, CDCl_3_) *δ* 143.5 ( = C_quat_), 131.8, 131.7, 131.6, 131.6, 131.5, 131.5, 128.6, 128.5, 128.4, 128.3, 128.2, 128.1, 127.9, 127.8 (C_Ar_), 109.7 (d, ^3^*J*_PC_ = 7.2 Hz, = CH_2_), 57.3 (d, ^1^*J*_PC_ = 73.2 Hz, CH-P), 25.8 (CH_2_), 12.7 (CH_3_) ppm; ^31^P-NMR (120 MHz, CDCl_3_) *δ* 30.9 ppm; ESI-HRMS (CI) *m/z* calcd. for C_17_H_21_NOPS ([M + H]^+^) 318.1081, found 318.1071.


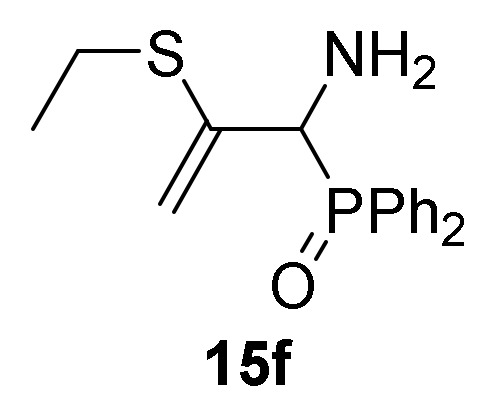


##### General Procedure and Spectral Data for the N-Tosyl Functionalization of Allylic α-Amino-phosphine Oxides and Phosphonates **15**

*p*-Toluenesulfonyl chloride (1 g, 5.5 mmol, 1.1 eq) and pyridine (2.4 mL, 30 mmol, 6 eq) were added to a 0 °C solution of derivative 15 (5 mmol, 1 eq) in CH_2_Cl_2_ (25 mL). The reaction mixture was allowed to reach 25 °C and stirred for 24 h. The crude product was washed twice with a 2M HCl solution (15 mL) and water (15 mL) and extracted with CH_2_Cl_2_ (15 mL). The organic layer was dried over anhydrous MgSO_4_, filtered and concentrated to dryness in vacuum. The crude product was purified by crystallization or by flash-column chromatography to afford *N*-tosyl allylic α-aminophosphine oxides and phosphonates 16.

*N-[1-(Diphenylphosphoryl)-2-(phenylthio)allyl]-4-methylbenzenesulfonamide* (**16a**), (2.26 g, 87%) obtained as a white solid from allylic α-aminophosphine oxide **15a** (1.83 g, 5 mmol) as described in the general procedure. The crude product was purified by flash-column chromatography (SiO_2_, EtOAc/hexane 10:20) to afford the title compound **16a**. mp 199–201 °C; IR (neat) *v*_max_ 3431, 3356, 3062, 2920, 2876, 2743, 1602, 1460, 1438, 1335, 1191, 1163, 1122, 1094, 1066, 911, 730 cm^−1^; ^1^H-NMR (400 MHz, CDCl_3_) *δ* 7.93–7.10 (m, 17H, Ar*H*), 6.72 (d, ^3^*J*_HH_ = 6.9 Hz, 2H, Ar*H*), 5.54 (d, ^2^*J*_HH_ = 2.5 Hz, 1H, = C*H*_2_), 4.83 (t, ^2^*J*_PH_ = 10.1 Hz, 1H, C*H*-P), 4.33 (d, ^2^*J*_HH_ = 2.7 Hz, 1H, = C*H*_2_), 2.37 (s, 3H, C*H*_3_) ppm; ^13^C {1H}-NMR (100 MHz, CDCl_3_) *δ* 142.7 ( = C_quat_), 139.4, 138.5, 134.4, 132.2, 132.1, 131.5, 131.4, 130.5, 129.1, 129.0, 128.7, 128.7, 128.6, 128.2, 128.1, 127.7 (C_Ar_), 115.2 (d, ^3^*J*_PC_ = 6.6 Hz, = CH_2_), 56.4 (d, ^1^*J*_PC_ = 74.1 Hz, CH-P), 21.5 (CH_3_) ppm; ^31^P-NMR (120 MHz, CDCl_3_) *δ* 30.8 ppm; ESI-HRMS (CI) *m/z* calcd. for C_28_H_27_NO_3_PS_2_ ([M + H]^+^) 520.1170, found 520.1174.


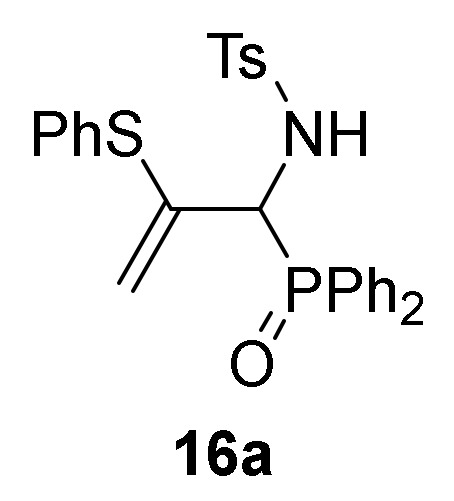


*N-[1-(Diphenylphosphoryl)-2-(p-tolylthio)allyl]-4-methylbenzene-sulfonamide* (**16b**), (2.24 g, 84%) obtained as an orange solid from allylic α-aminophosphine oxide **15b** (1.90 g, 5 mmol) as described in the general procedure. The crude product was purified by crystallization from Et_2_O/CH_2_Cl_2_ 50:50 to afford the title compound **16b**. mp 180–182 °C; IR (neat) *v*_max_ 3428, 3059, 2926, 2870, 1599, 1438, 1333, 1191, 1160, 911, 739 cm^−1^; ^1^H-NMR (400 MHz, CDCl_3_) *δ* 7.86–7.41 (m, 12H, Ar*H*), 7.13 (d, ^3^*J*_HH_ = 8.5 Hz, 2H, Ar*H*), 6.99 (d, ^3^*J*_HH_ = 7.8 Hz, 2H, Ar*H*), 6.60 (d, ^3^*J*_PH_ = 8.0 Hz, 2H, Ar*H*), 5.37 (d, ^2^*J*_HH_ = 2.7 Hz, 1H, = C*H*_2_), 4.82 (t, ^2^*J*_PH_ = 10.0 Hz, 1H, C*H*-P), 4.26 (d, ^2^*J*_HH_ = 2.5 Hz, 1H, = C*H*_2_), 2.38 (s, 3H, C*H*_3_), 2.28 (s, 3H, C*H*_3_) ppm; ^13^C {1H}-NMR (75 MHz, CDCl_3_) *δ* 142.9 ( = C_quat_), 140.3 (C_quat_Ar), 139.0 (C_quat_Ar), 138.1 (C_quat_Ar), 134.6, 132.3, 132.2, 132.2, 132.1, 131.5, 131.4, 129.9, 129.1, 128.8, 128.6, 128.2, 128.0, 127.7, 126.6 (C_Ar_), 114.0 (d, ^3^*J*_PC_ = 7.0 Hz, = CH_2_), 56.4 (d, ^1^*J*_PC_ = 73.8 Hz, CH-P), 21.5 (CH_3_), 21.2 (CH_3_) ppm; ^31^P-NMR (120 MHz, CDCl_3_) *δ* 31.2 ppm; ESI-HRMS (CI) *m/z* calcd. for C_29_H_29_NO_3_PS_2_ ([M + H]^+^) 534.1326, found 534.1329.


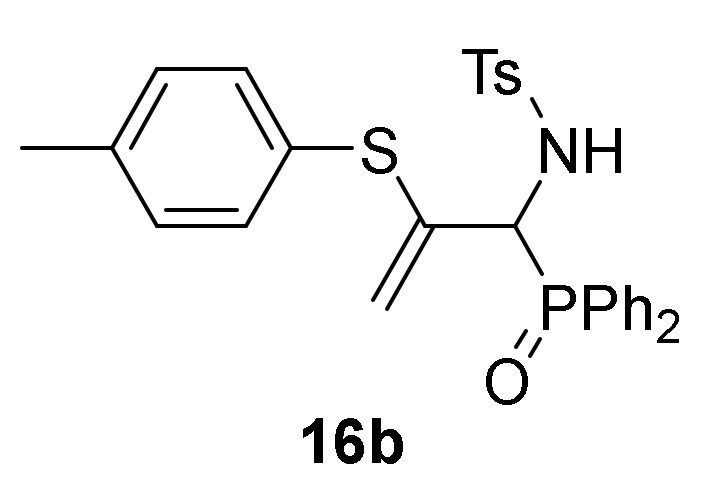


*Diethyl [1-((4-methylphenyl)sulfonamido)-2-(phenylthio)allyl]phosphonate* (**16c**), (1.93 g, 85%) obtained as a pale yellow solid from allylic α-aminophosphonate **15e** (1.51 g, 5 mmol) as described in the general procedure. The crude product was purified by flash-column chromatography (SiO_2_, AcOEt/hexane 17:83) to afford the title compound **16c**. mp 99–101 °C; IR (neat) *v*_max_ 3126, 2990, 2927, 1600, 1480, 1438, 1337, 1242, 1166, 1055, 1027, 910, 726 cm^−1^; ^1^H-NMR (300 MHz, CDCl_3_) *δ* 7.75 (d, ^3^*J*_HH_ = 8.2 Hz, 2H, Ar*H*), 7.29–7.11 (m, 7H, Ar*H*), 6.82 (dd, ^3^*J*_PH_ = 4.4 Hz, ^3^*J*_HH_ = 9.8 Hz, 1H, N*H*), 5.45 (d, ^2^*J*_HH_ = 3.9 Hz, 1H, = C*H*_2_), 4.65 (d, ^2^*J*_HH_ = 3.7 Hz, 1H, = C*H*_2_), 4.36 (dd, ^3^*J*_HH_ = 9.9 Hz, ^2^*J*_PH_ = 24.4 Hz, 1H, C*H*-P), 4.25–4.08 (m, 4H, OC*H*_2_), 2.41 (s, 3H, C*H*_3_), 1.31 (q, ^3^*J*_HH_ = 7.0 Hz, 6H, C*H*_3_) ppm; ^13^C {1H}-NMR (75 MHz, CDCl_3_) *δ* 143.1 ( = C_quat_), 139.8 (C_quat_Ar), 138.1 (C_quat_Ar), 134.1, 131.0, 129.2, 129.1, 128.6, 127.5 (C_Ar_), 115.4 (d, ^3^*J*_PC_ = 9.1 Hz, = CH_2_), 64.1 (d, ^2^*J*_PC_ = 7.0 Hz, OCH_2_), 63.8 (d, ^2^*J*_PC_ = 7.0 Hz, OCH_2_), 55.0 (d, ^1^*J*_PC_ = 157.6 Hz, CH-P), 21.4 (CH_3_), 16.6 (d, ^3^*J*_PC_ = 5.8 Hz, CH_3_) ppm; ^31^P-NMR (120 MHz, CDCl_3_) *δ* 18.4 ppm; ESI-HRMS (CI) *m/z* calcd. for C_20_H_27_NO_5_PS_2_ ([M + H]^+^) 456.1068, found 456.1071.


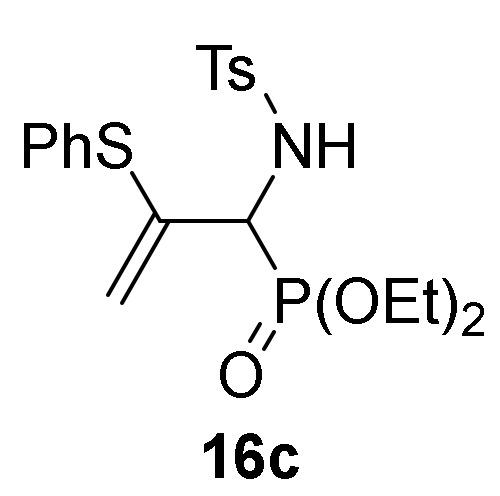


##### One pot procedure for the synthesis of N-tosyl allylic α-aminophosphonates **16d–e**

To a 0 °C solution of 2*H*-azirine **1e** (0.96 g, 5 mmol) in CH_2_Cl_2_ (25 mL) was added dropwise the corresponding *p*-substituted benzenethiol (5.5 mmol, 1.1 eq). The reaction mixture was stirred at 0 °C for 48 h until TLC showed the disappearance of starting compound **1e**. The reaction mixture was concentrated to dryness in vacuum to afford derivatives **15**. Without any further purification step, to a 0 °C solution of crude products **15** in CH_2_Cl_2_ (25 mL) was directly added *p*-toluenesulfonyl chloride (1 g, 5.5 mmol, 1.1 eq) and pyridine (2.42 mL, 30 mmol, 6 eq). The reaction mixture was allowed to reach 25 °C and stirred for 24 h. The crude product was washed twice with a 2M HCl solution (15 mL) and water (15 mL) and extracted with CH_2_Cl_2_ (15 mL). The organic layer was dried over anhydrous MgSO_4_, filtered and concentrated to dryness in vacuum. The crude products **16d**–**e** were purified by flash-column chromatography.

*Diethyl [2-((4-fluorophenyl)thio)-1-((4-methylphenyl)sulfonamido)-allyl]-phosphonate* (**16d**)**,** (2.01 g, 85%) obtained as a pale yellow solid from 2*H*-azirine **1e** (0.96 g, 5 mmol) and 4-fluorobenzenethiol (**13c**) (0.59 mL, 5.5 mmol) in a one pot reaction as described in the general procedure. The crude product was purified by flash-column chromatography (SiO_2_, EtOAc/hexane 10:30) to afford the title compound **16d**. mp 117–119 °C; IR (neat) *v*_max_ 3370, 3161, 2984, 2927, 1594, 1492, 1239, 1166, 1052, 1030, 907, 739 cm^−1^; ^1^H-NMR (300 MHz, CDCl_3_) *δ* 8.02 (d, ^3^*J*_HH_ = 8.0 Hz, 2H, Ar*H*), 7.54–7.21 (m, 4H, Ar*H*), 7.52 (d, ^3^*J*_HH_ = 8.1 Hz, 2H, Ar*H*), 6.99 (dd, ^3^*J*_HH_ = 9.8 Hz, ^3^*J*_PH_ = 4.5 Hz, 1H, N*H*), 5.70 (d, ^2^*J*_HH_ = 4.3 Hz, 1H, = C*H*_2_), 4.87 (d, ^2^*J*_HH_ = 3.6 Hz, 1H, = C*H*_2_), 4.62 (dd, ^3^*J*_HH_ = 9.8 Hz, ^2^*J*_PH_ = 24.4 Hz, 1H, C*H*-P), 4.53–4.31 (m, 4H, OC*H*_2_), 2.70 (s, 3H, C*H*_3_), 1.61–1.55 (m, 6H, C*H*_3_) ppm; ^13^C {1H}-NMR (75 MHz, CDCl_3_) *δ* 164.7 (C_Ar_-F), 161.4 (C_quat_), 143.2 ( = C_quat_), 140.3, 138.1, 136.6, 136.5, 129.2, 127.5, 116.5, 116.3 (C_Ar_), 115.0 (d, ^3^*J*_PC_ = 9.1 Hz, = CH_2_), 64.2 (d, ^2^*J*_PC_ = 7.0 Hz, OCH_2_), 63.8 (d, ^2^*J*_PC_ = 7.0 Hz, OCH_2_), 54.9 (d, ^1^*J*_PC_ = 157.5 Hz, CH-P), 21.4 (CH_3_), 16.4 (CH_3_), 16.3 (CH_3_) ppm; ^31^P-NMR (120 MHz, CDCl_3_) *δ* 18.3 ppm; ^19^F NMR (282 MHz, CDCl_3_) *δ* –112.1 ppm; ESI-HRMS (CI) *m/z* calcd. for C_20_H_26_FNO_5_PS_2_ ([M + H]^+^) 474.0974, found 474.0976.


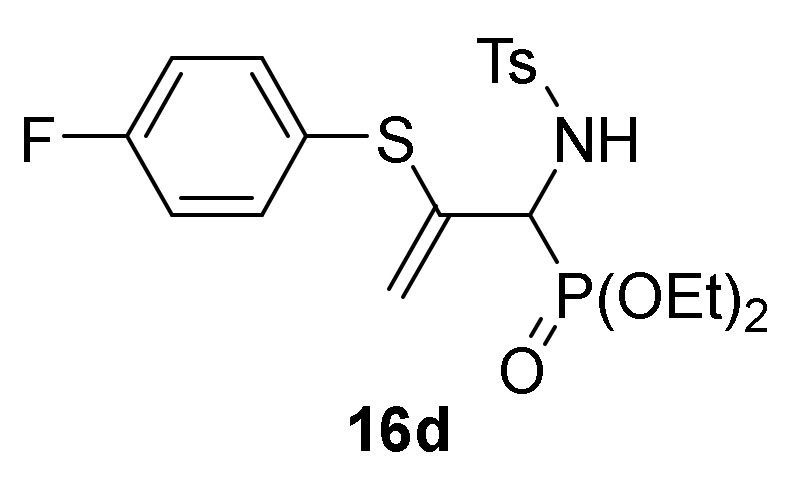


*Diethyl [1-((4-methylphenyl)sulfonamido)-2-(p-tolylthio)allyl]phos-phonate* (**16e**), (1.71 g, 73%) obtained as a pale yellow solid from 2*H*-azirine **1e** (0.96 g, 5 mmol) and 4-methylbenzenethiol (**13b**) (0.68 g, 5.5 mmol) in a one pot reaction as described in the general procedure. The crude product was purified by flash-column chromatography (SiO_2_, EtOAc/hexane 10:20) to afford the title compound **16e**. mp 168–170 °C; IR (neat) *v*_max_ 3128, 2979, 2934, 2867, 1596, 1491, 1446, 1341, 1247, 1149, 1011, 961, 905, 819 cm^−1^; ^1^H-NMR (300 MHz, CDCl_3_) *δ* 7.73 (d, ^3^*J*_HH_ = 8.3 Hz, 2H, Ar*H*), 7.31–6.98 (m, 6H, Ar*H*), 6.34 (dd, ^3^*J*_HH_ = 9.8 Hz, ^3^*J*_PH_ = 4.5 Hz, 1H, N*H*), 5.32 (d, ^2^*J*_HH_ = 4.1 Hz, 1H, = C*H*_2_), 4.55 (d, ^2^*J*_HH_ = 2.7 Hz, 1H, = C*H*_2_), 4.35 (dd, ^3^*J*_HH_ = 9.9 Hz, ^2^*J*_PH_ = 24.2 Hz, 1H, C*H*-P), 4.24–4.03 (m, 4H, OC*H*_2_), 2.41 (s, 3H, C*H*_3_), 2.31 (s, 3H, C*H*_3_), 1.30 (t, ^3^*J*_HH_ = 7.0 Hz, 6H, C*H*_3_) ppm; ^13^C {1H}-NMR (75 MHz, CDCl_3_) *δ* 143.2 ( = C_quat_), 140.6 (C_quat_), 139.0 (C_quat_), 138.0 (C_quat_), 134.6, 130.0, 129.2, 127.6, 127.1 (C_quat_) (C_Ar_), 114.2 (d, ^3^*J*_PC_ = 9.2 Hz, = CH_2_), 64.1 (d, ^2^*J*_PC_ = 7.3 Hz, OCH_2_), 63.9 (d, ^2^*J*_PC_ = 6.9 Hz, OCH_2_), 55.1 (d, ^1^*J*_PC_ = 157.4 Hz, CH-P), 21.5 (CH_3_), 21.2 (CH_3_), 16.4 (CH_3_), 16.3 (CH_3_) ppm; ^31^P-NMR (120 MHz, CDCl_3_) *δ* 18.5 ppm; ESI-HRMS (CI) *m/z* calcd. for C_21_H_29_NO_5_PS_2_ ([M + H]^+^) 470.1225, found 470.1229.


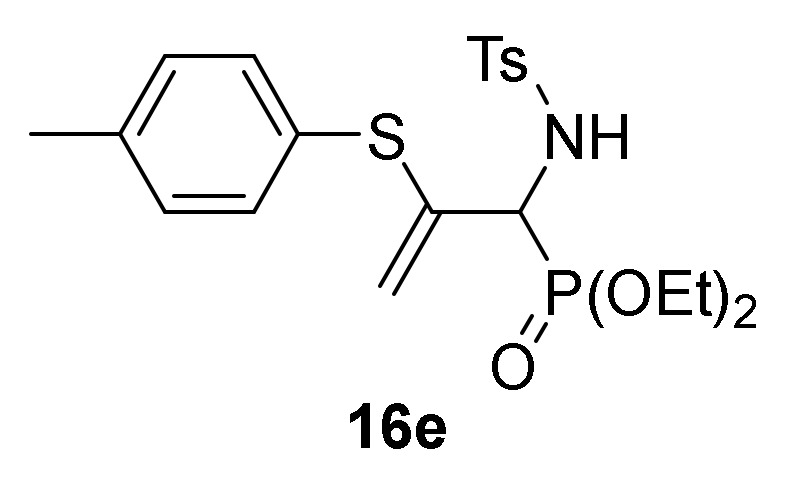


### 3.2. Biology

#### 3.2.1. Materials

Reagents and solvents were used as purchased without further purification. All stock solutions of the investigated compounds were prepared by dissolving the powered materials in appropriate amounts of DMSO. The final concentration of DMSO never exceeded 5% (v/v) in reactions. The stock solution was stored at 5 °C until it was used.

#### 3.2.2. Cytotoxicity Assays

Cells were cultured according to the supplier’s instructions. Cells were seeded in 96-well plates at a density of 2–4 × 103 cells per well and incubated overnight in 0.1 mL of media supplied with 10% Fetal Bovine Serum (Lonza) in 5% CO2 incubator at 37 °C. On day 2, compounds were added and samples were incubated for 48 h. After treatment, 10 μL of cell counting kit-8 was added into each well for additional 2 h incubation at 37 °C. The absorbance of each well was determined by an Automatic ELISA Reader System (Multiskan FC, Thermo Fisher Scientific, Waltham, MA, USA) at 450 nm wavelength.

## 4. Conclusions

To sum up, we have develop a very efficient new approach to α-aminophosphine oxide and phosphonate acetals **4**, through the nucleophilic addition of methanol or ethanol to the carbon-nitrogen double bond of 2*H*-azirine and subsequent ring opening through the N–C3 bond. Conversely, addition of *O*-nucleophiles such as 2,2,2-trifluoroethanol or even phenols to phosphorylated 2*H*-azirines, gave to the regioselective formation of allylic α-aminophosphorus derivatives **8** and **11**. Initially aziridine intermediate formation, following carbon-carbon double bond construction and ring opening by means of the N–C3 aziridine bond occurred to afford compounds **8** and **11**. Under these reaction conditions, in some cases, aziridine intermediates **7** and **10** can be isolated and characterized. To the best of our acknowledge, this process exemplifies the first example of a regioselective nucleophilic addition of oxygen nucleophiles to the carbon-nitrogen double bond of a phosphorus substituted 2*H*-azirine with the formation of allylic α-aminophosphorus derivatives. Furthermore, *N*-functionalization of α-aminophosphine oxide and phosphonate acetals **4** or allylic α-aminophosphorus derivatives **8** and **11** were assessed by sulfonylation reaction.

As an extension of our previous results, we have broadened this process through the addition of sulfur nucleophiles to phosphorylated 2*H*-azirines, with the synthesis of novel sulfur-containing allylic α-aminophosphine oxides and phosphonates **15**.

Oxygen and sulfur containing α-allylic phosphine oxides and phosphonates, here synthesized, might be regarded as new hybrid molecules introducing two potential pharmacophores, allylic amine and α-aminophosphonic acid moieties. These new hybrid molecules may retain the functional properties of the parent molecules. Moreover, the therapeutic efficiency of all the synthesized α-aminophosphorus derivatives and aziridines was evaluated against the human cancer cell line A549. The best cytotoxic effect was observed for α-aminophosphonate acetal **4f** with an IC_50_ value of 1.3 ± 0.10 µM, allylic α-aminophosphine oxide **11a** with a IC_50_ value of 1.9 ± 0.13 µM, as well as for **15c** with an IC_50_ value of 0.1 ± 0.08 µM. Whereas, colon carcinoma cell line (RKO) is not so sensitive to some of the tested synthesized compounds. In addition, cytotoxic effect of almost all of our compounds in non-malignant lung fibroblasts (MRC-5) seems not to exhibit any effect.

## Figures and Tables

**Table 1:** α-Aminophosphine oxides and phosphonates **4**, **5** and **6** obtained.

Entry	Compound	R	R^1^	R^2^	Yield (%) ^1^
1	**4a**	Ph	Me	Me	74
2	**4b**	Ph	Et	Me	92
3	**4c**	Ph	Ph	Me	81
4	**4d**	Ph	Me	Et	56
5	**4e**	O*^i^*Pr	Me	Me	69
6	**4f**	OEt	Me	Me	61
7	**5a**	Ph	Me	Me	80
8	**5b**	O*^i^*Pr	Me	Me	62
9	**5c**	OEt	Me	Me	64
10	**5d**	OEt	Me	Et	70 ^2^
11	**6**	O*^i^*Pr	Me		68

^1^ Yield of isolated purified compounds **4**, **5** and **6**. ^2^ One pot reaction from 2*H*-azirine **1e**.

**Table 2:** Aziridines **10** and allyl α-aminophosphine oxides and phosphonates **11** and **12** obtained.

Entry	Compound ^1^	R	Ar	Yield (%) ^2^
1	**10a**	Ph	Ph	70
2	**10b**	Ph	2-Naph	^3^
3	**11a**	Ph	Ph	93
4	**11b**	Ph	2-Naph	74
5	**12a**	OEt	Ph	88 ^4^
6	**12b**	OEt	2-Naph	67 ^4^

^1^ All synthesized compounds are racemic. ^2^ Yield of isolated purified compounds **10**, **11**, and **12**. ^3^ Identified compound in the crude reaction mixture. ^4^ One pot reaction from 2*H*-azirine **1e**.

**Table 3:** Aziridines **14** and allylic α-aminophosphine oxides and phosphonates **15** and **16** obtained.

Entry	Compound	R	R^1^	R^2^	Yield (%) ^1^
1	**14a**	Ph	Me	Ph	92 ^2^
2	**14b**	Ph	Me	*p-*MeC_6_H_4_	^3,4^
3	**14c**	Ph	Ph	Ph	60 ^2^
4	**15a**	Ph		Ph	91 ^2^
5	**15b**	Ph		*p-*MeC_6_H_4_	89 ^4^
6	**15c**	Ph		*p-*FC_6_H_4_	76 ^4^
7	**15d**	Ph		*p-*MeOC_6_H_4_	70 ^4^
8	**15e**	OEt		Ph	41 ^4^
9	**15f**	Ph		Et	64 ^2^
10	**16a**	Ph		Ph	87
11	**16b**	Ph		*p-*MeC_6_H_4_	84
12	**16c**	OEt		Ph	85
13	**16d**	OEt		*p-*FC_6_H_4_	85 ^5^
14	**16e**	OEt		*p-*MeC_6_H_4_	73 ^5^

^1^ Yield of isolated purified compounds **14**, **15**, and **16**. ^2^ Using method A. ^3^ Identified compound in the crude reaction mixture. ^4^ Using method B. ^5^ One pot reaction from 2H-azirine **1e**.

**Table 4:** Antiproliferative activity of synthesized compounds obtained from the addition of alcohols and phenols to 2*H*-azirines **1**.

Entry	Comp.	R	R^1^	R^2^	Cytotoxicity IC_50_ (µM) ^1^	
					Lung A549	MRC-5
1	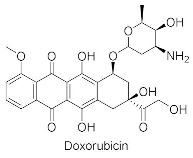				0.48 ± 0.017 [50]	>50 [51]
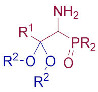						
2 ^2^	**4a**	Ph	Me	Me	4.4 ± 0.72	>50
3	**4b**	Ph	Et	Me	21.3 ± 0.22	>50
4	**4c**	Ph	Ph	Me	16.1 ± 2.03	>50
5	**4d**	Ph	Me	Et	9.6 ± 1.13	>50
6	**4e**	O*^i^*Pr	Me	Me	4.6 ± 0.31	>50
7	**4f**	OEt	Me	Me	1.3 ± 0.10	>50
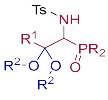						
8	**5a**	Ph	Me	Me	8.2 ± 0.23	>50
9	**5b**	O*^i^*Pr	Me	Me	1.7 ± 0.30	>50
10	**5c**	OEt	Me	Me	4.5 ± 0.45	>50
11	**5d**	OEt	Me	Et	3.7 ± 0.49	>50
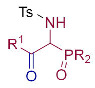						
12	**6**	O*^i^*Pr	Me		>50	^3^
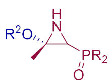						
13	**7**	Ph		CH_2_CF_3_	3.6 ± 0.70	>50
14	**10a**	Ph		Ph	13.3 ± 1.69	>50
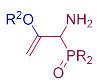						
15	**8**	Ph		CH_2_CF_3_	>50	>50
16 ^2^	**11a**	Ph		Ph	1.9 ± 0.13	>50
17	**11b**	Ph		2-Naph	2.7 ± 0.44	33.6 ± 3.73
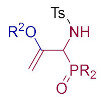						
18	**9**	Ph		CH_2_CF_3_	3.5 ± 0.77	>50
19	**12a**	OEt		Ph	4.8 ± 0.90	>50
20	**12b**	OEt		2-Naph	2.1 ± 0.22	17.5 ± 1.47

^1^ The cytotoxicity IC_50_ values listed are the concentrations corresponding to 50% growth inhibition. ^2^ The cytotoxicity value against human colon carcinoma cell line (RKO) is >50 µM. ^3^ Not determined.

**Table 5:** Antiproliferative activity of synthesized compounds obtained from the addition of thiophenols to 2*H*-azirines **1**.

Entry	Comp.	R	R^1^	R^2^	Cytotoxicity IC_50_ (µM) ^1^	
					Lung A549	MRC-5
1	DOX				0.48 ± 0.017 [27]	>50 [28]
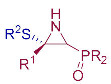						
2 ^2^	**14c**	Ph	Ph	Ph	1.1 ± 0.32	4.9 ± 0.49
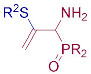						
3 ^3^	**15a**	Ph		Ph	2.6 ± 0.68	15.9 ± 2.79
4	**15b**	Ph		*p*-MeC_6_H_4_	5.1 ± 0.77	14.9 ± 1.61
5 ^3^	**15c**	Ph		*p*-FC_6_H_4_	0.1 ± 0.08	>50
6	**15d**	Ph		*p*-MeOC_6_H_4_	2.6 ± 0.42	>50
7	**15e**	OEt		Ph	7.2 ± 0.49	>50
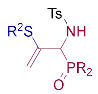						
8	**16a**	Ph		Ph	1.2 ± 0.09	>50
9	**16b**	Ph		*p*-MeC_6_H_4_	2.1 ± 0.15	>50
10 ^3^	**16c**	OEt		Ph	0.2 ± 0.07	24.1 ± 3.55
11	**16d**	OEt		*p*-FC_6_H_4_	3.0 ± 0.98	>50
12	**16e**	OEt		*p*-MeC_6_H_4_	3.9 ± 0.63	>50

^1^ The cytotoxicity IC_50_ values listed are the concentrations corresponding to 50% growth inhibition. ^2^ The cytotoxicity value against human colon carcinoma cell line (RKO) is 9.7 ± 1.4 µM. ^3^ The cytotoxicity value against human colon carcinoma cell line (RKO) is >50 µM.

## Supplementary Materials

The following are available online, ^1^H- and ^13^C-NMR spectra of compounds **4–12**, **14**–**16**.
