# Supplementary material for: Synthesis of α-Aminophosphonic Acid Derivatives Through the Addition of O- and S-Nucleophiles to 2H-Azirines and Their Antiproliferative Effect on A549 Human Lung Adenocarcinoma Cells
Source: Molecules. 2020 Jul 22;25(15):3332. doi: 10.3390/molecules25153332 (PMC7435673; doi:10.3390/molecules25153332)

Electronic Supporting Information

Synthesis of α-aminophosphonic acid derivatives through the addition of *O*- and *S*-nucleophiles to 2*H*-azirines and their antiproliferative effect on A549 human lung adenocarcinoma cells.

Victor Carramiñana, Ana M. Ochoa de Retana, Francisco Palacios*, Jesús M. de los Santos*

Department of Organic Chemistry I, Faculty of Pharmacy and Lascaray Research Center, University of the Basque Country (UPV/EHU), Paseo de la Universidad 7, 01006 Vitoria, Spain.

E-mail addresses: francisco.palacios@ehu.eus / jesus.delossantos@ehu.eus

Table of contents

^{1}H and ^{13}C NMR spectra of α-aminophosphine oxide and phosphonate acetals **4a–f** Page S–2

^{1}H and ^{13}C NMR spectra of *N*-tosyl α-aminophosphine oxide and phosphonate acetals **5a–d** Page S–8

^{1}H and ^{13}C NMR spectra of β-keto-α-aminophosphonate **6** Page S–12

^{1}H and ^{13}C NMR spectra of aziridine phosphine oxide **7** Page S–13

^{1}H and ^{13}C NMR spectra of allylic α-aminophosphine oxide **8** Page S–14

^{1}H and ^{13}C NMR spectra of allylic *N*-tosyl-α-aminophosphine oxide **9** Page S–15

^{1}H and ^{13}C NMR spectra of aziridine phosphine oxides **10a–b** Page S–16

^{1}H and ^{13}C NMR spectra of allylic α-aminophosphine oxides **11a–b** Page S–18

^{1}H and ^{13}C NMR spectra of allylic *N*-tosyl-α-aminophosphonate **12a–b** Page S–20

^{1}H and ^{13}C NMR spectra of aziridine phosphine oxides **14a–b** Page S–22

^{1}H and ^{13}C NMR spectra of allylic α-aminophosphine oxides and phosphonates **15b–d**,**f** Page S–24

^{1}H and ^{13}C NMR spectra of allylic *N*-tosyl-α-aminophosphine oxides and phosphonates **16a–e** Page S–28

^{1}H NMR (300 MHz, $CDCl_3$) of compound **4a**

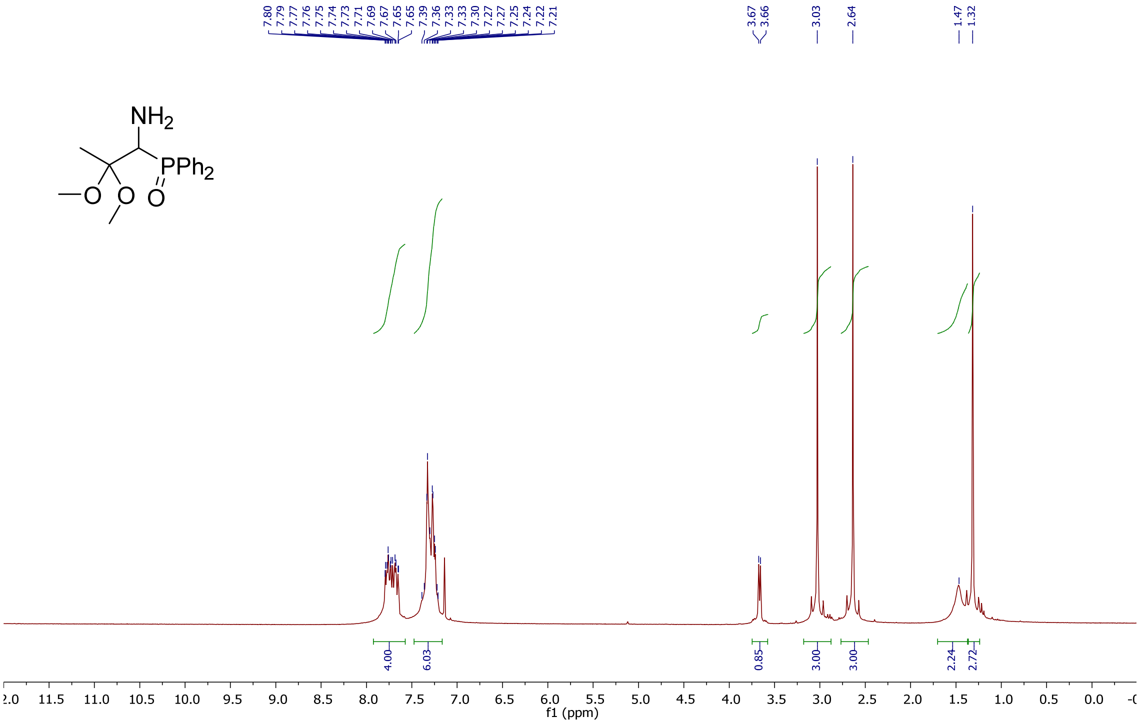

^{13}C {^{1}H} NMR (75 MHz, $CDCl_3$) of compound **4a**

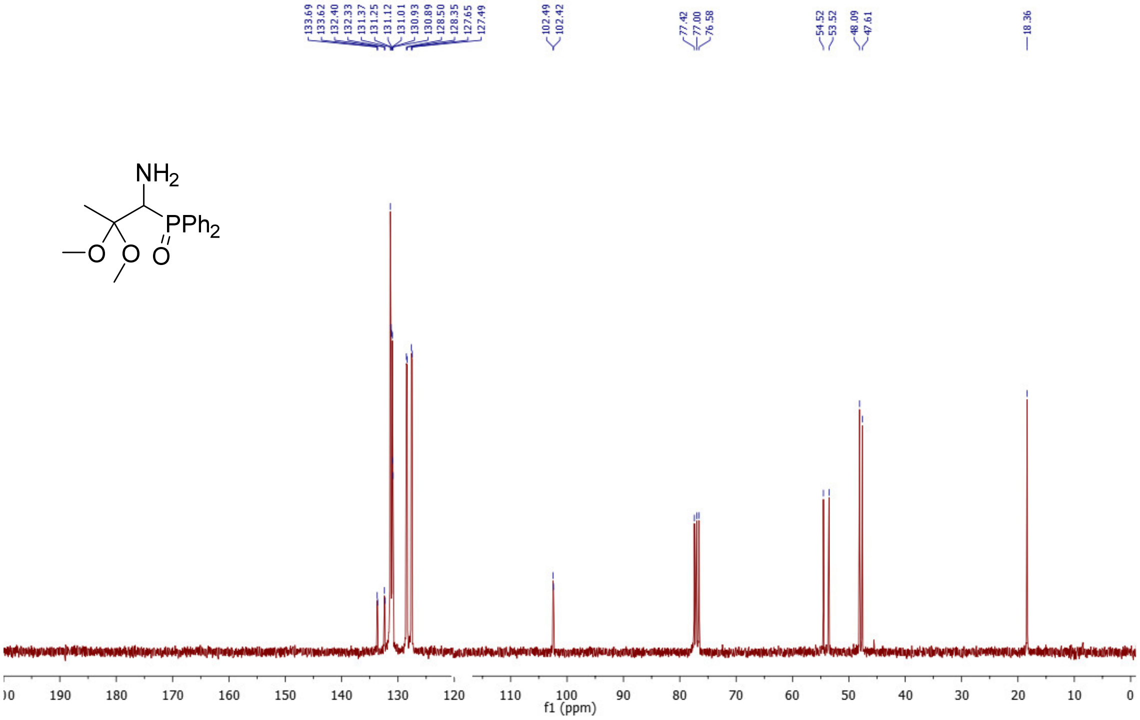

^{1}H NMR (400 MHz, $CDCl_3$) of compound **4b**

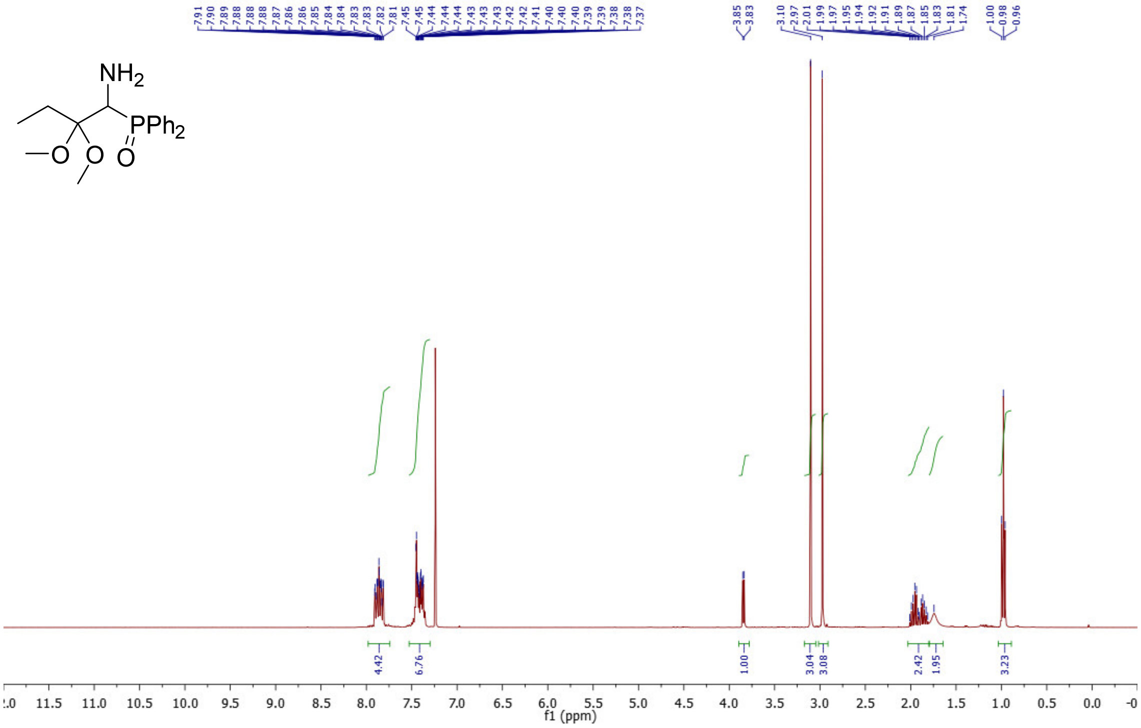

^{13}C {^{1}H} NMR (100 MHz, $CDCl_3$) of compound **4b**

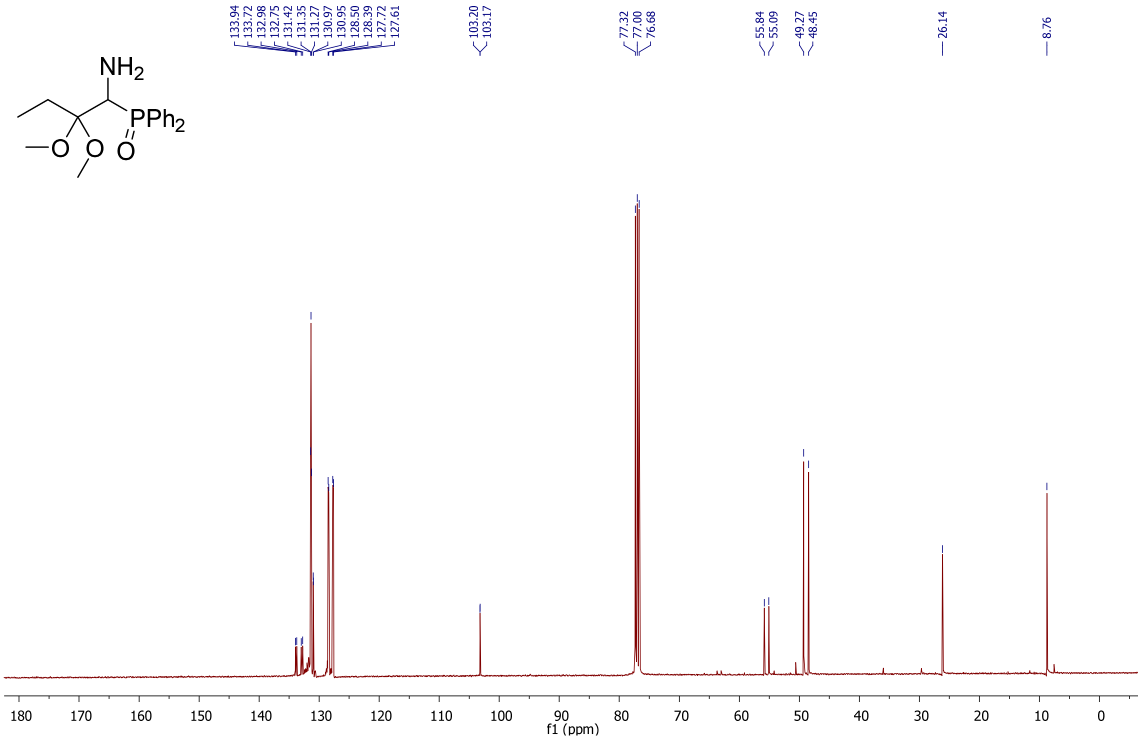

^{1}H NMR (400 MHz, $CDCl_3$) of compound **4c**

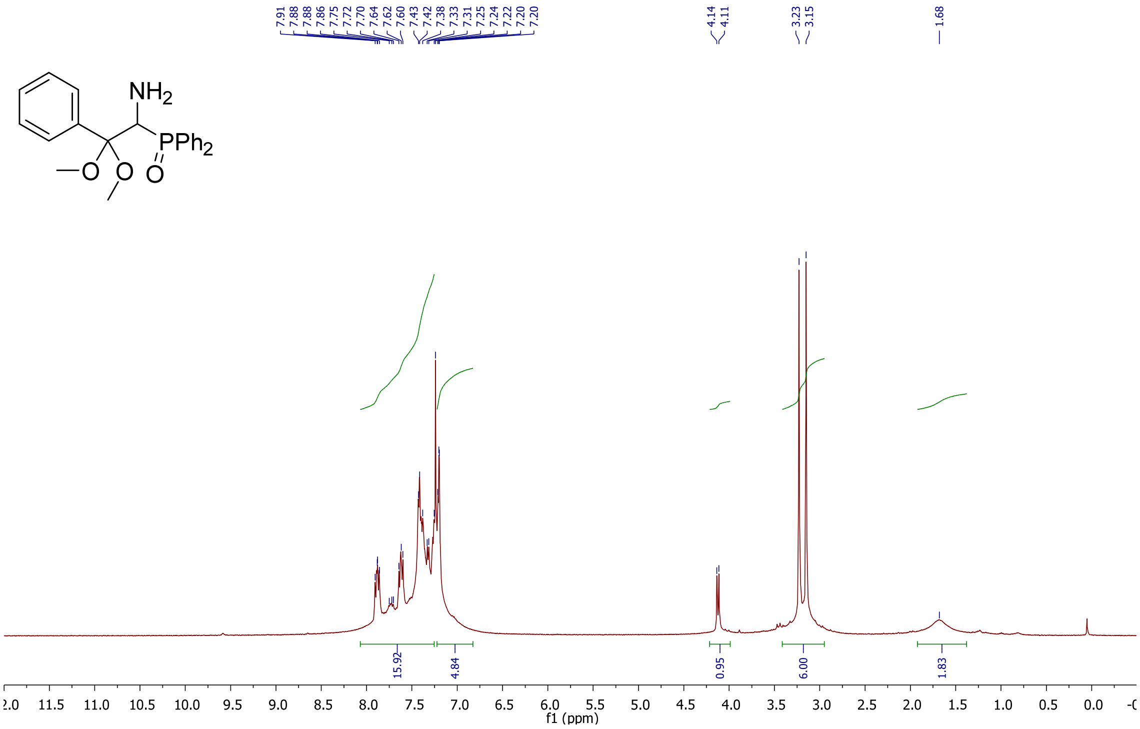

^{13}C {^{1}H} NMR (75 MHz, $CDCl_3$) of compound **4c**

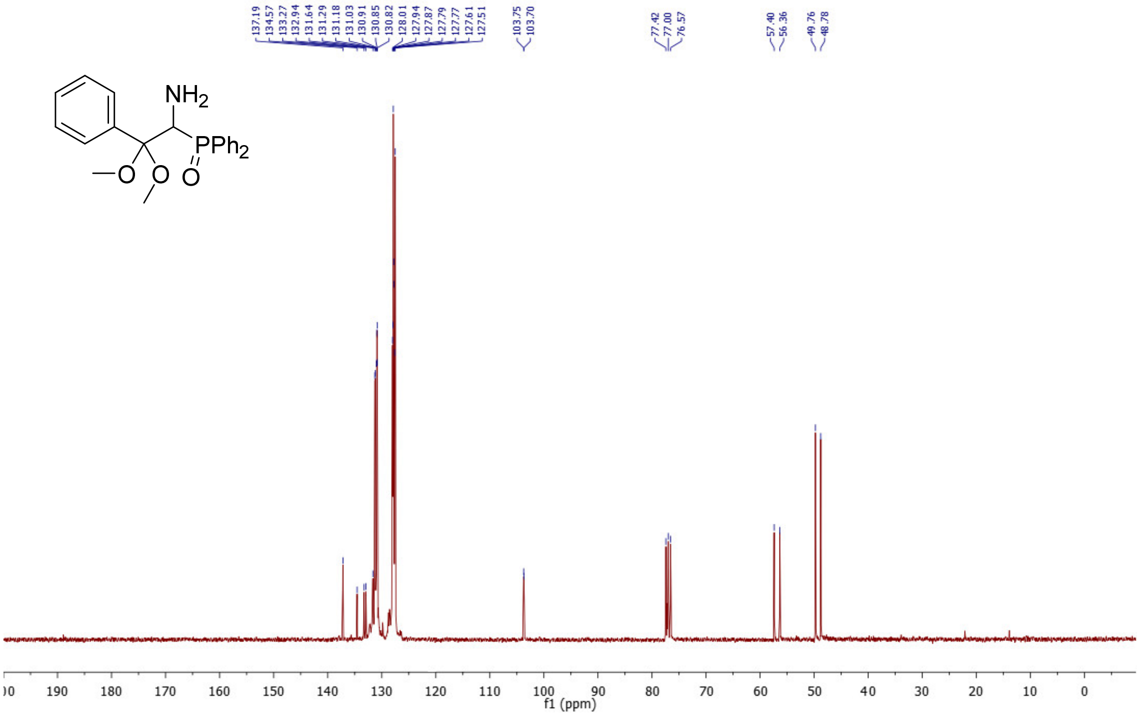

^{1}H NMR (300 Hz, $CDCl_3$) of compound **4d**

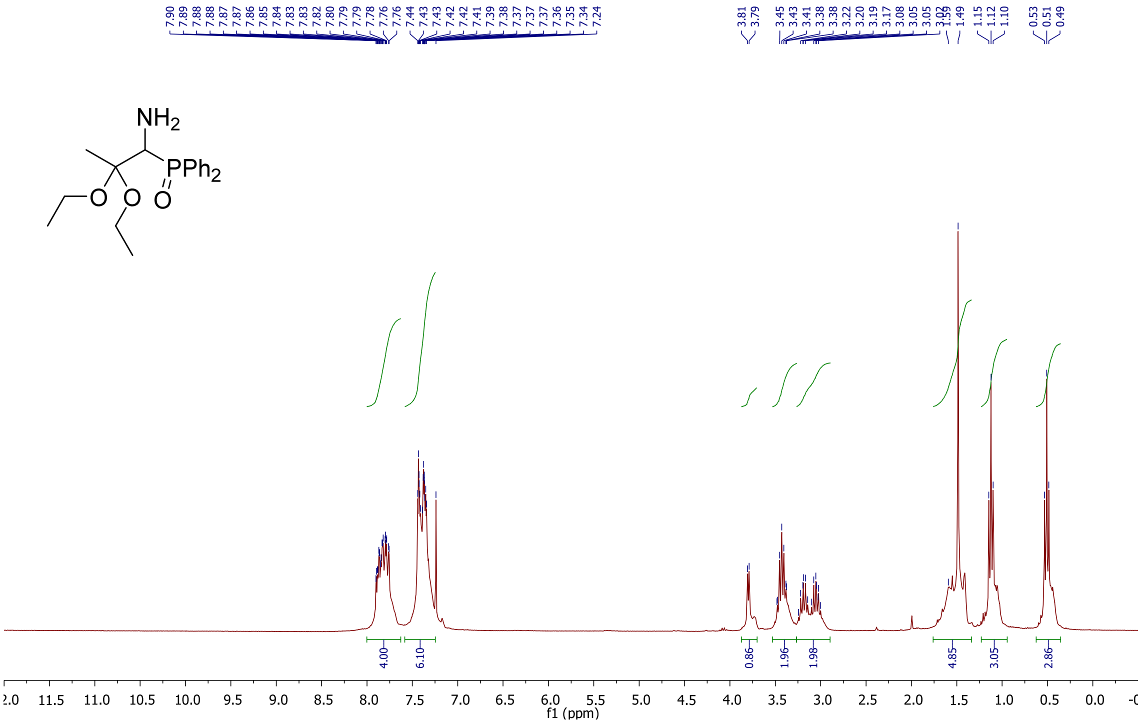

^{13}C {^{1}H} NMR (75 MHz, $CDCl_3$) of compound **4d**

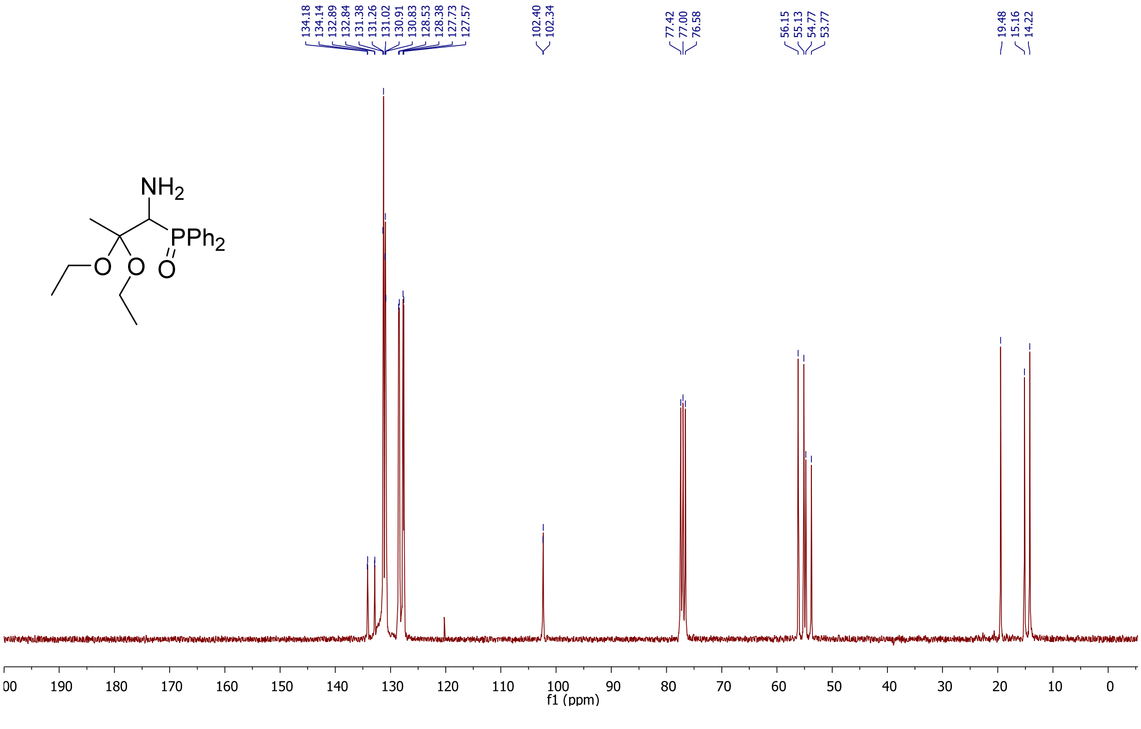

^{1}H NMR (400 MHz, $CDCl_3$) of compound **4e**

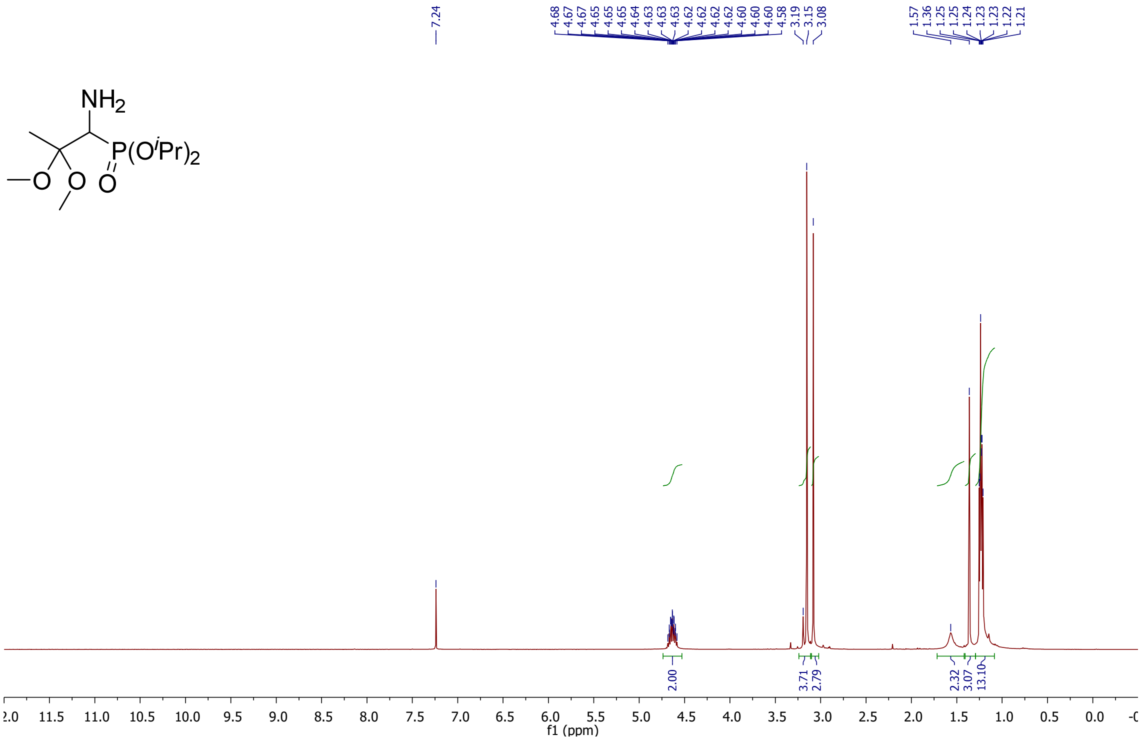

^{13}C $\{^{1}H\}$ NMR (100 MHz, $CDCl_3$) of compound **4e**

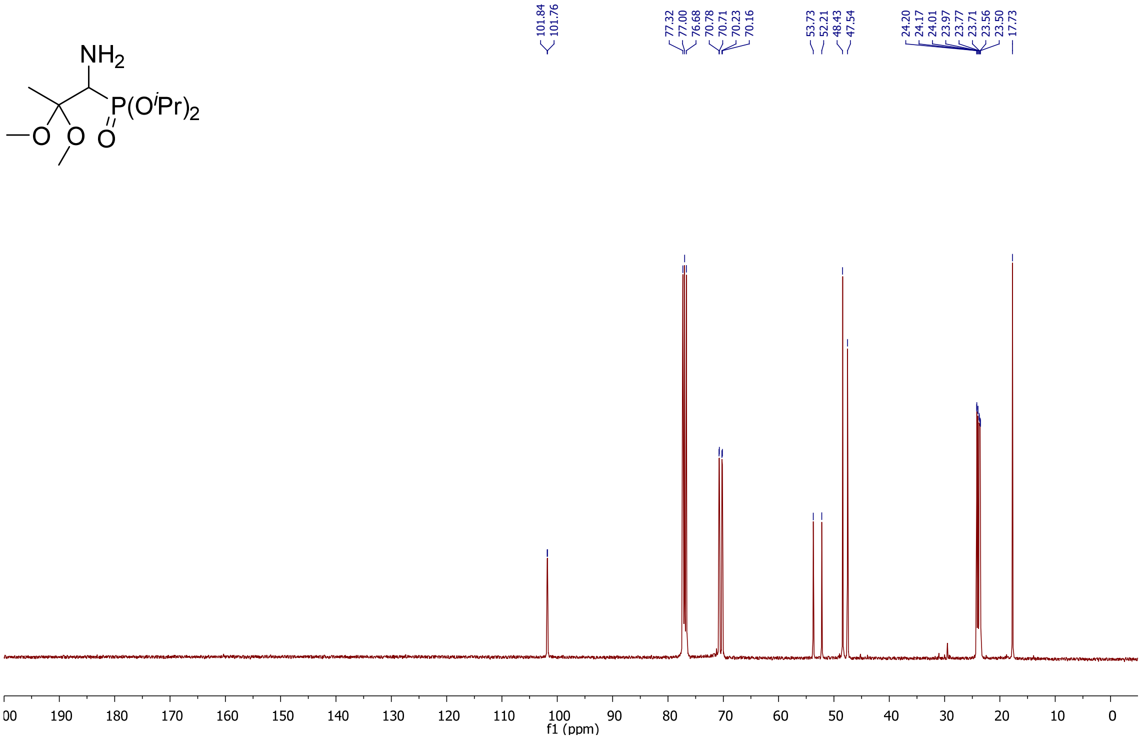

^{1}H NMR (400 MHz, $CDCl_3$) of compound **4f**

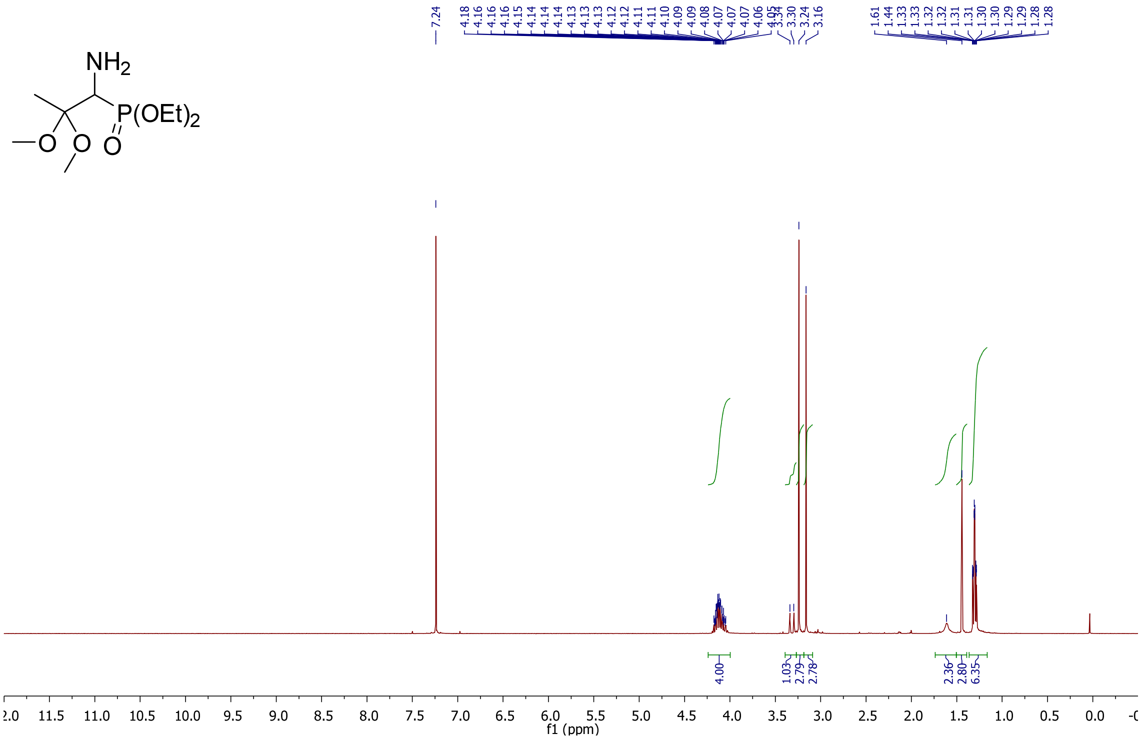

^{13}C {^{1}H} NMR (100 MHz, $CDCl_3$) of compound **4f**

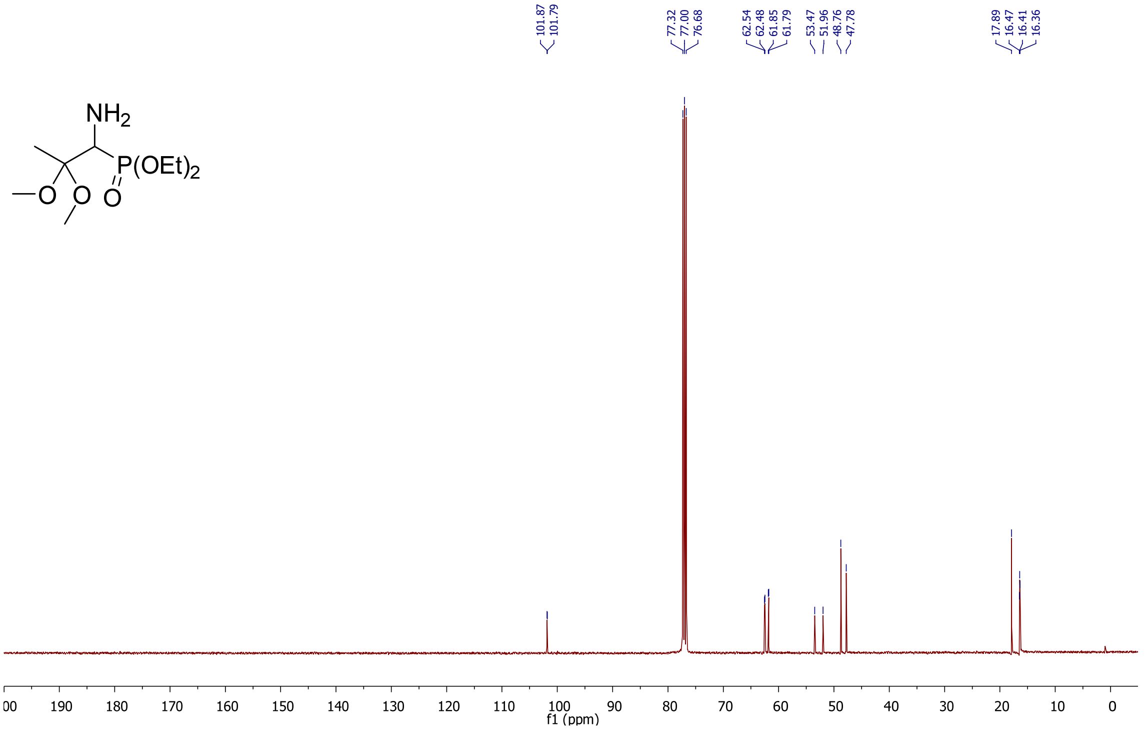

^{1}H NMR (400 MHz, $CDCl_3$) of compound **5a**

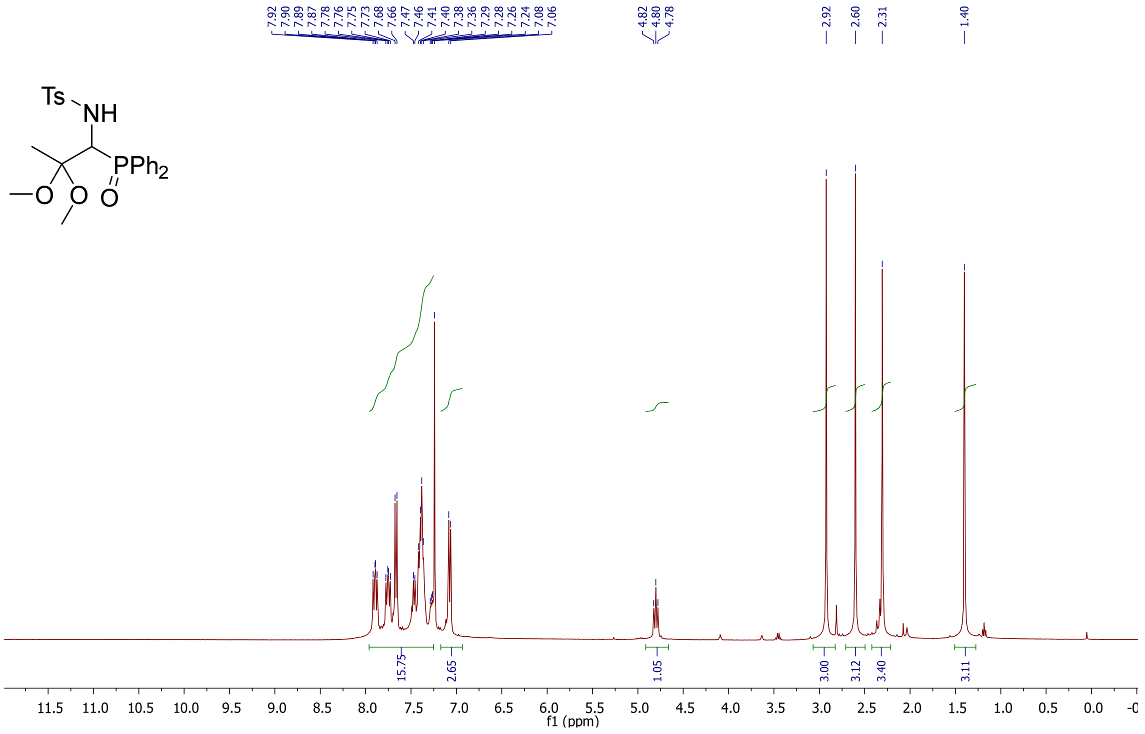

^{13}C {^{1}H} NMR (100 MHz, $CDCl_3$) of compound **5a**

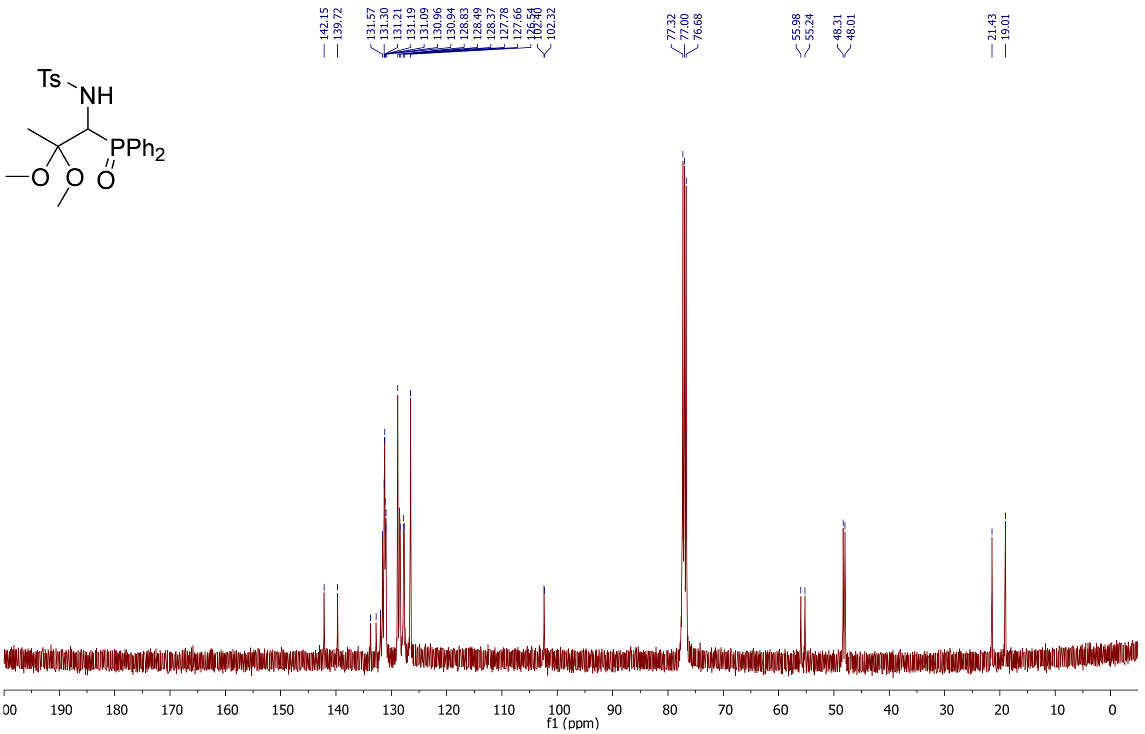

^{1}H NMR (400 MHz, $CDCl_3$) of compound **5b**

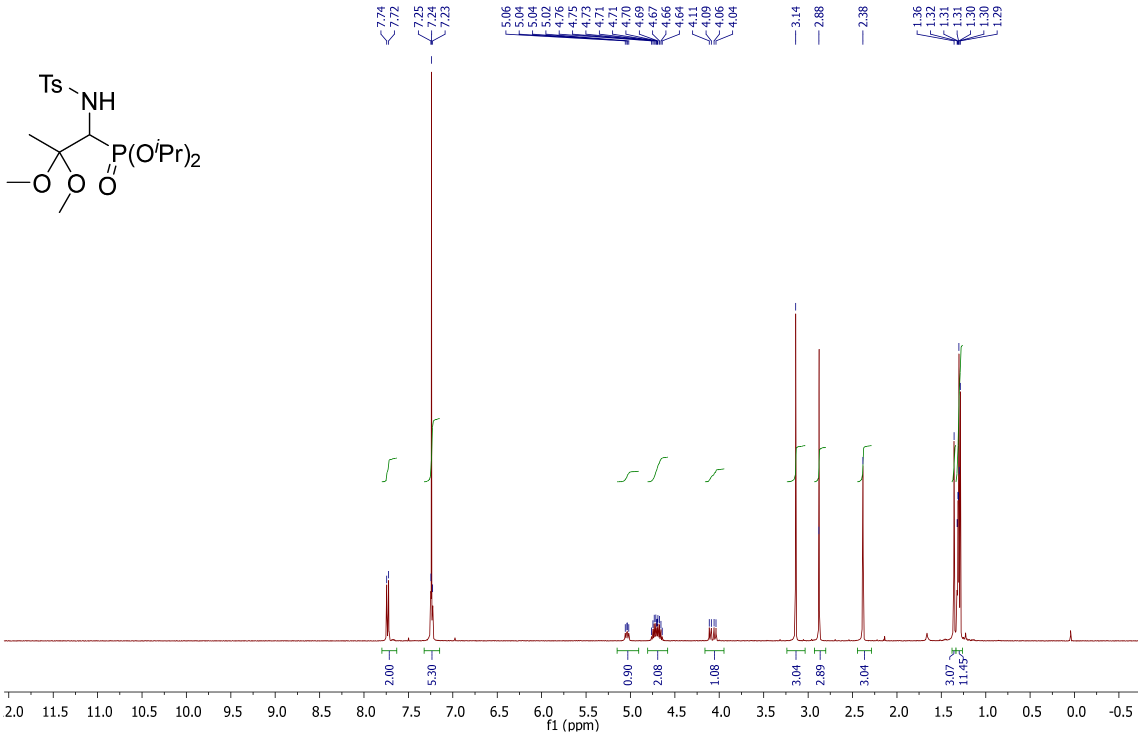

^{13}C {^{1}H} NMR (75 MHz, $CDCl_3$) of compound **5b**

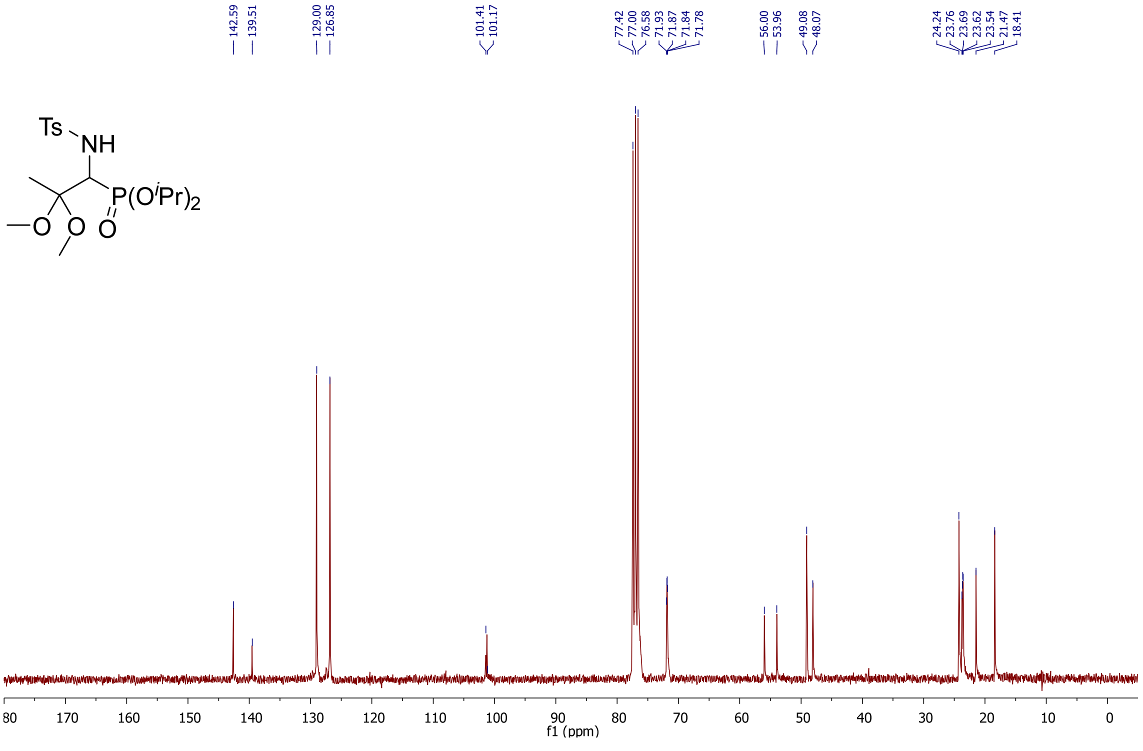

^{1}H NMR (400 MHz, $CDCl_3$) of compound **5c**

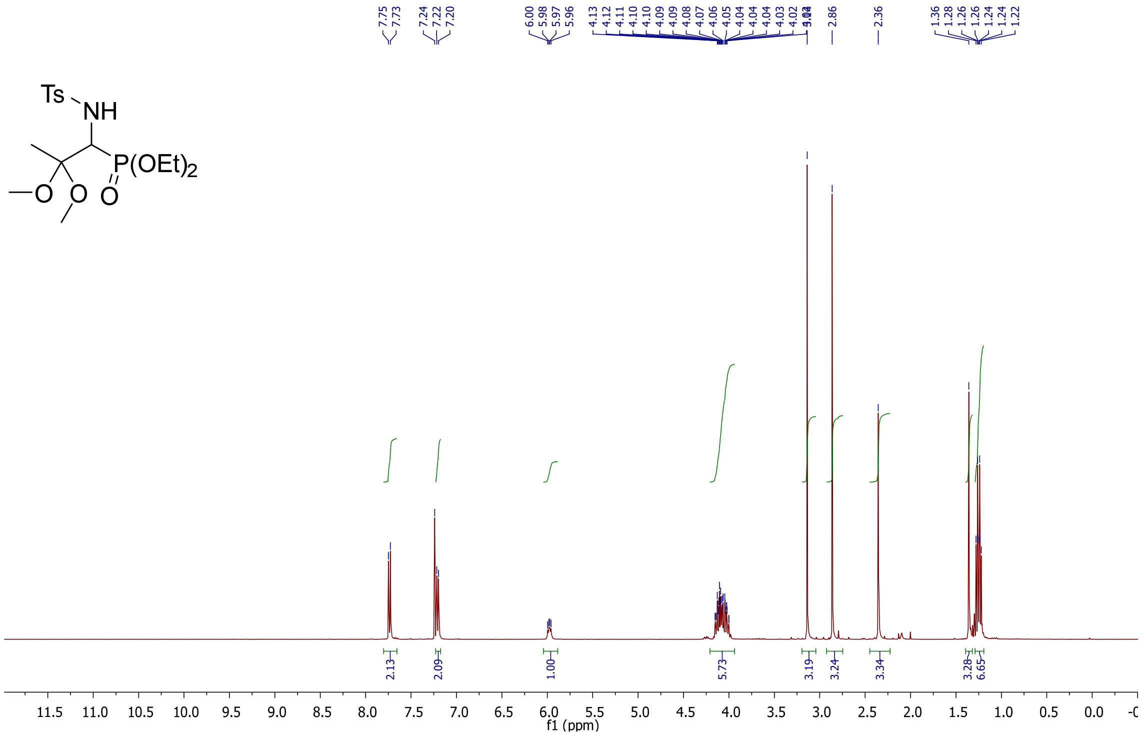

^{13}C {^{1}H} NMR (75 MHz, $CDCl_3$) of compound **5c**

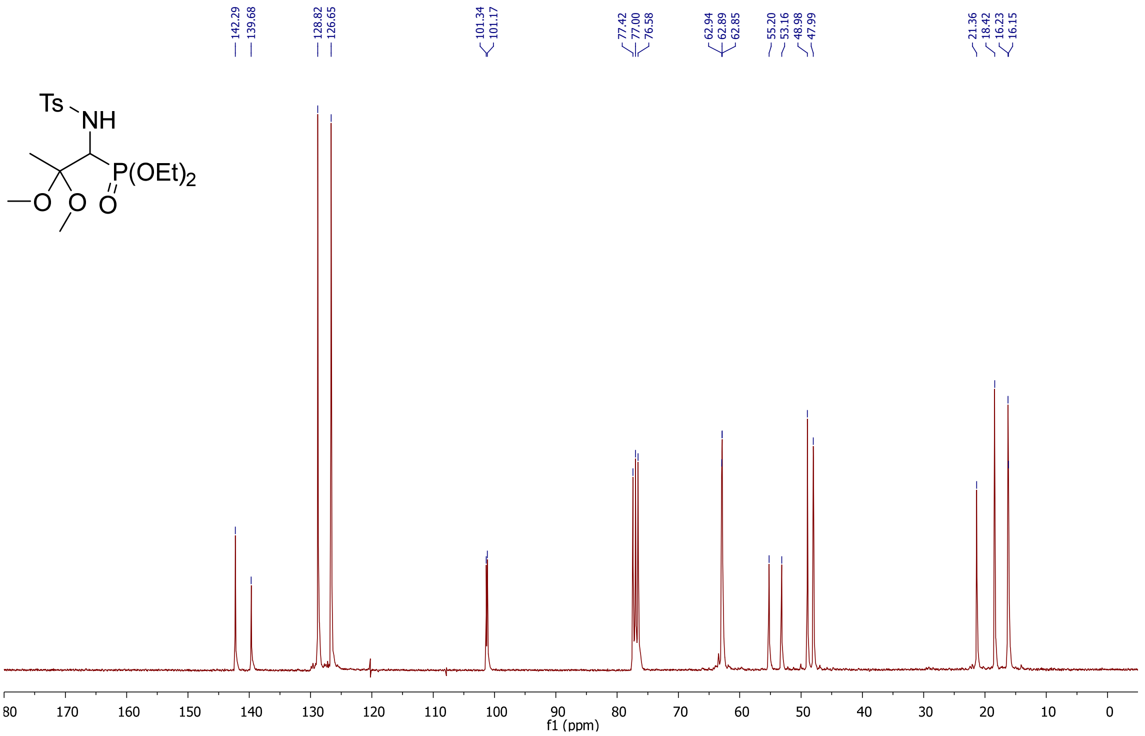

¹H NMR (300 Hz, $CDCl_3$) of compound **5d**

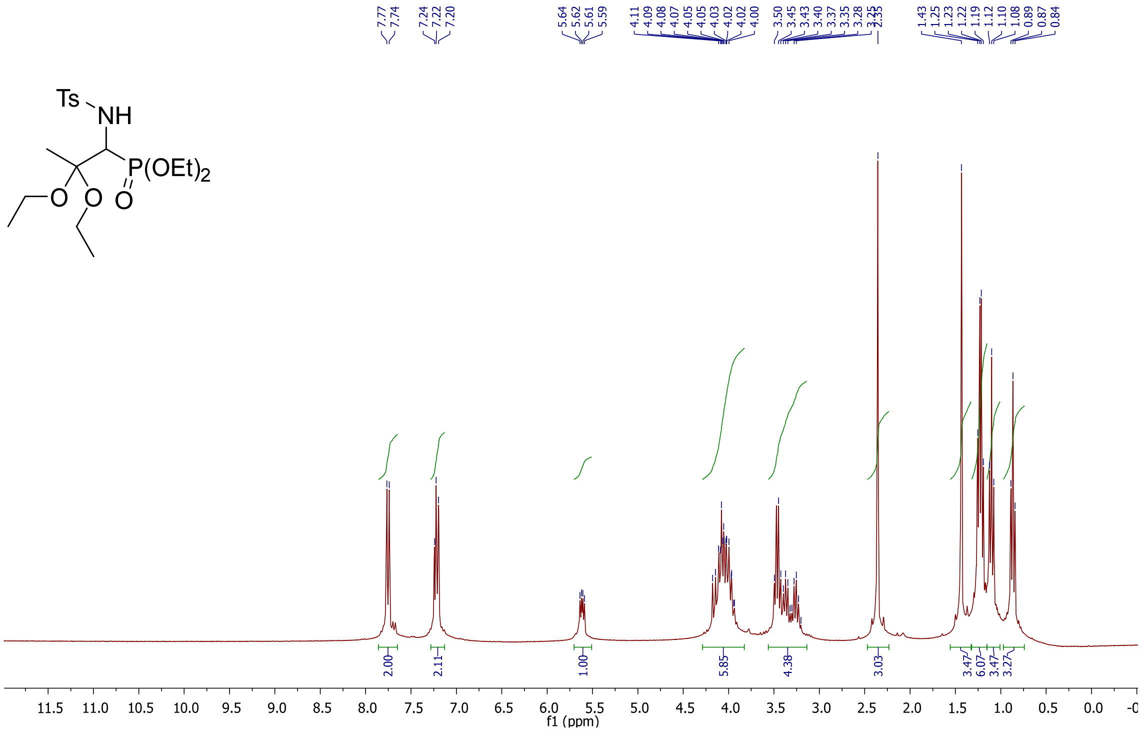

¹³C {¹H} NMR (75 MHz, $CDCl_3$) of compound **5d**

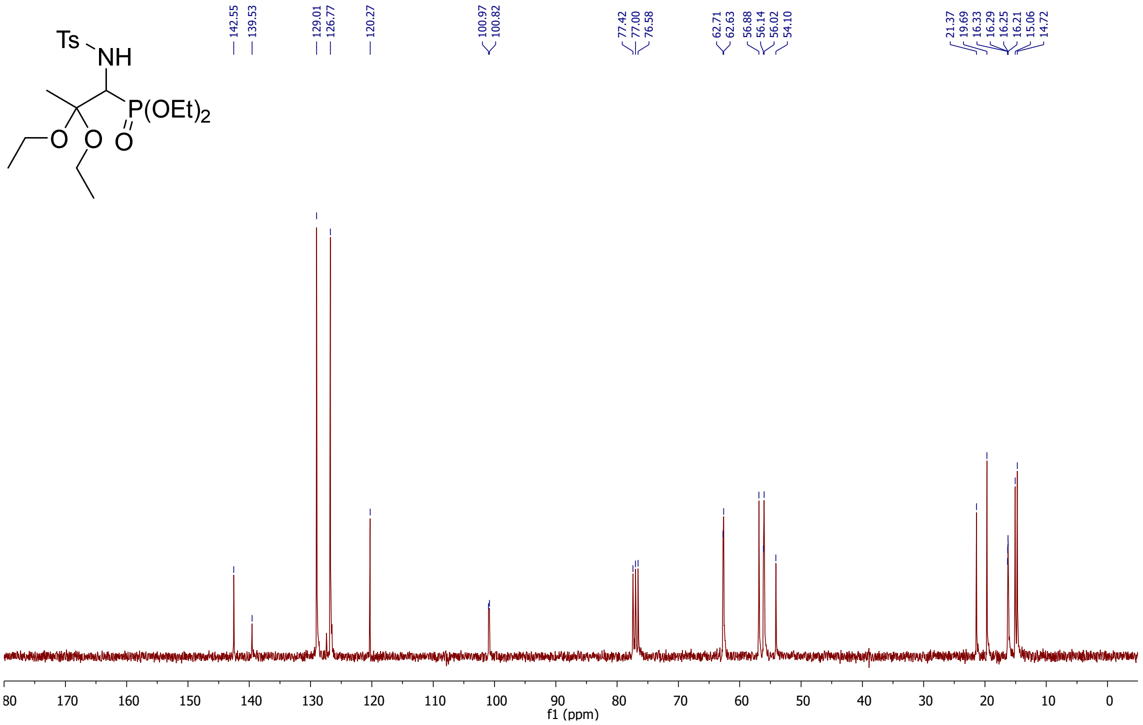

^{1}H NMR (400 MHz, $CDCl_3$) of compound **6**

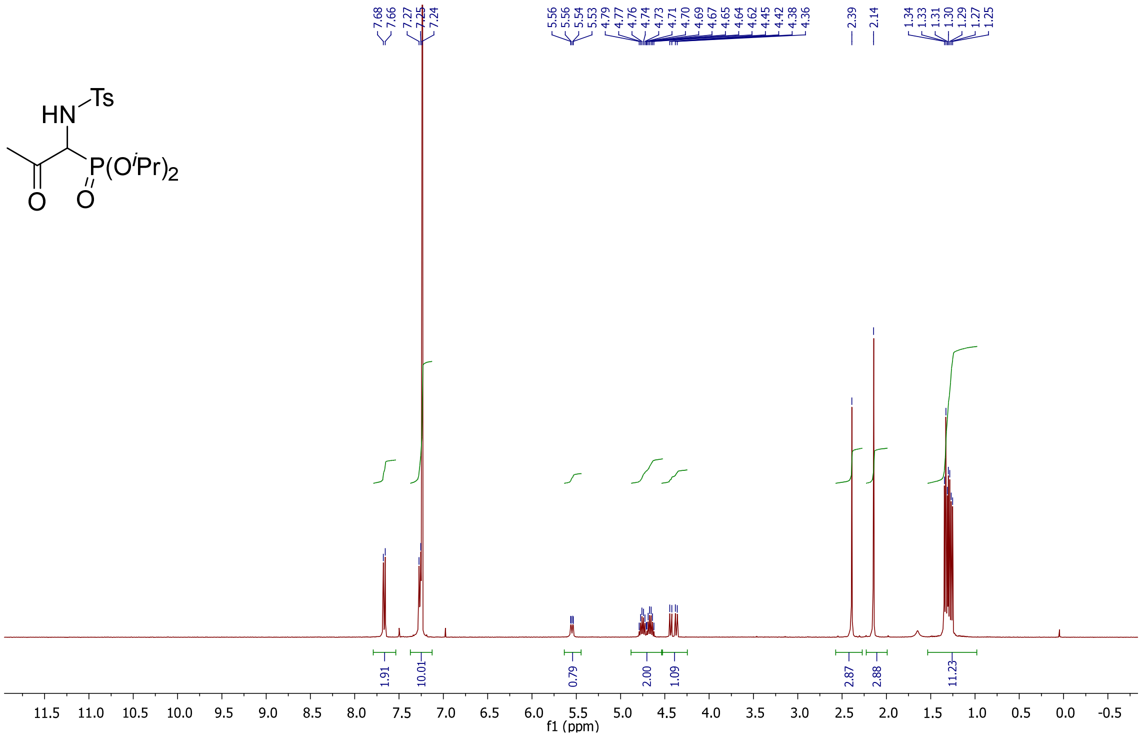

^{13}C {^{1}H} NMR (100 MHz, $CDCl_3$) of compound **6**

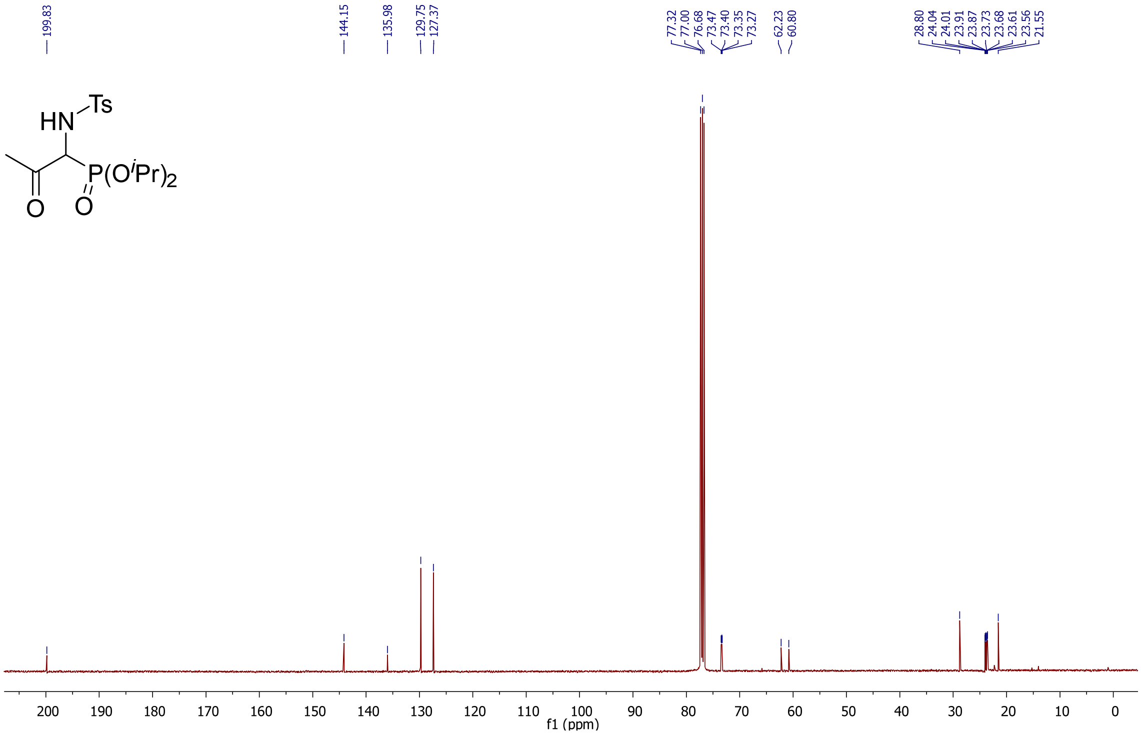

^{1}H NMR (300 Hz, $CDCl_3$) of compound **7**

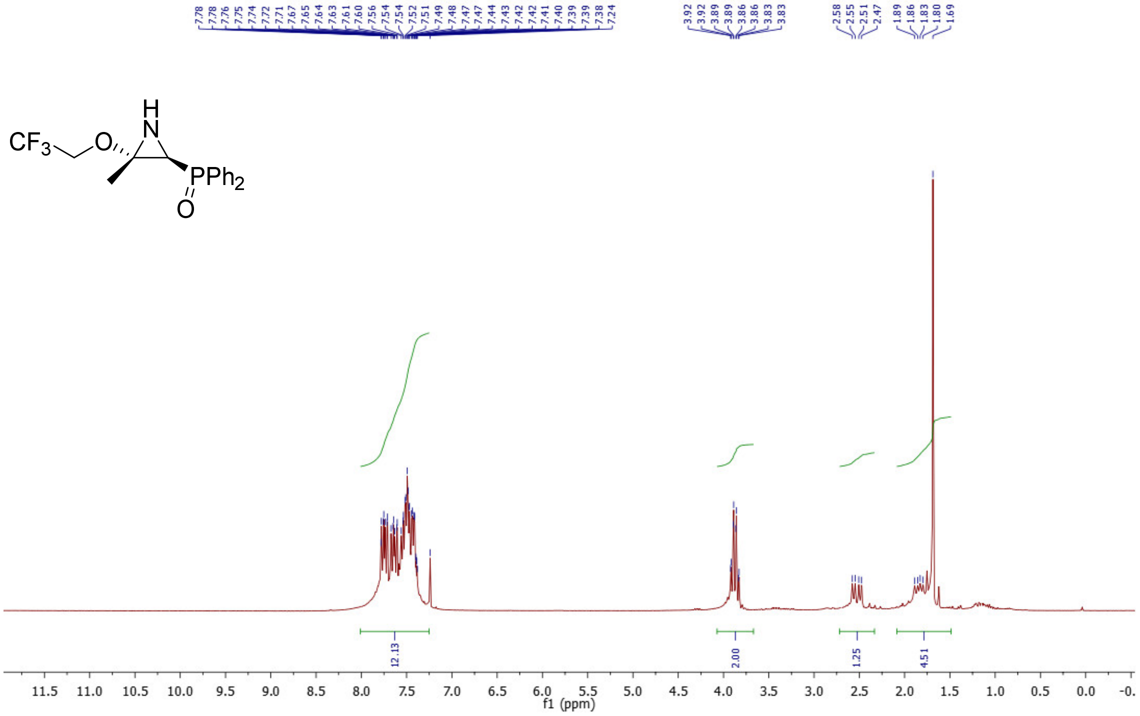

^{13}C {^{1}H} NMR (75 MHz, $CDCl_3$) of compound **7**

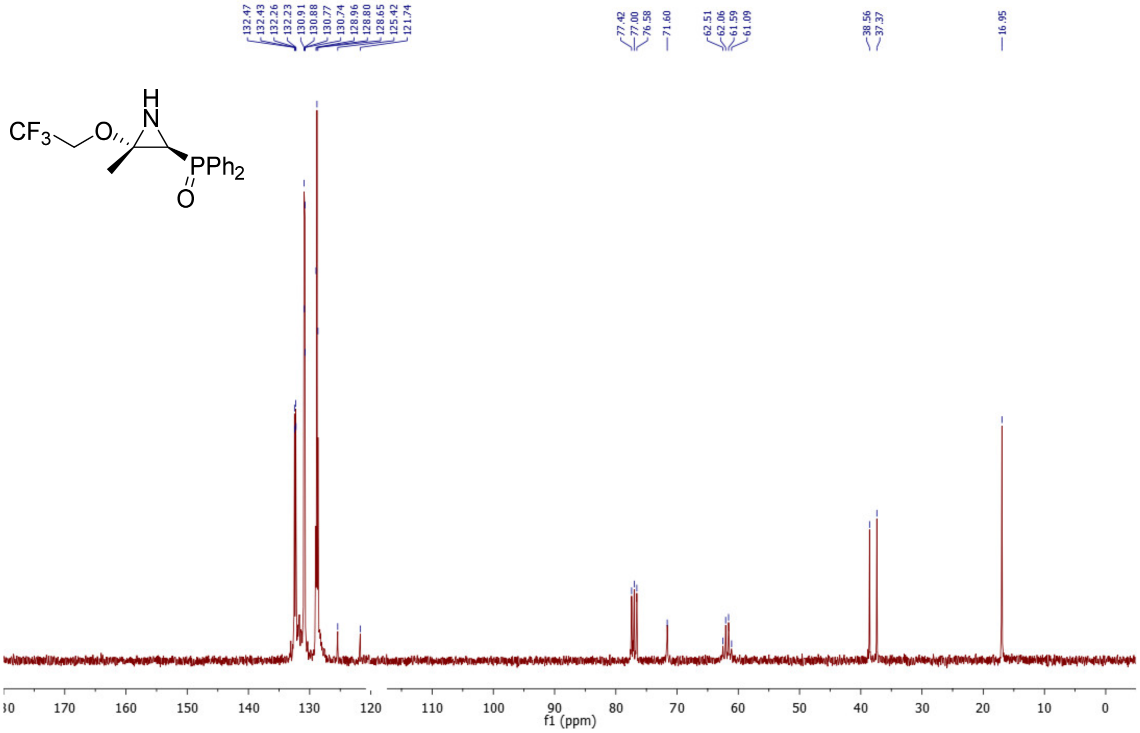

^{1}H NMR (300 Hz, $CDCl_3$) of compound **8**

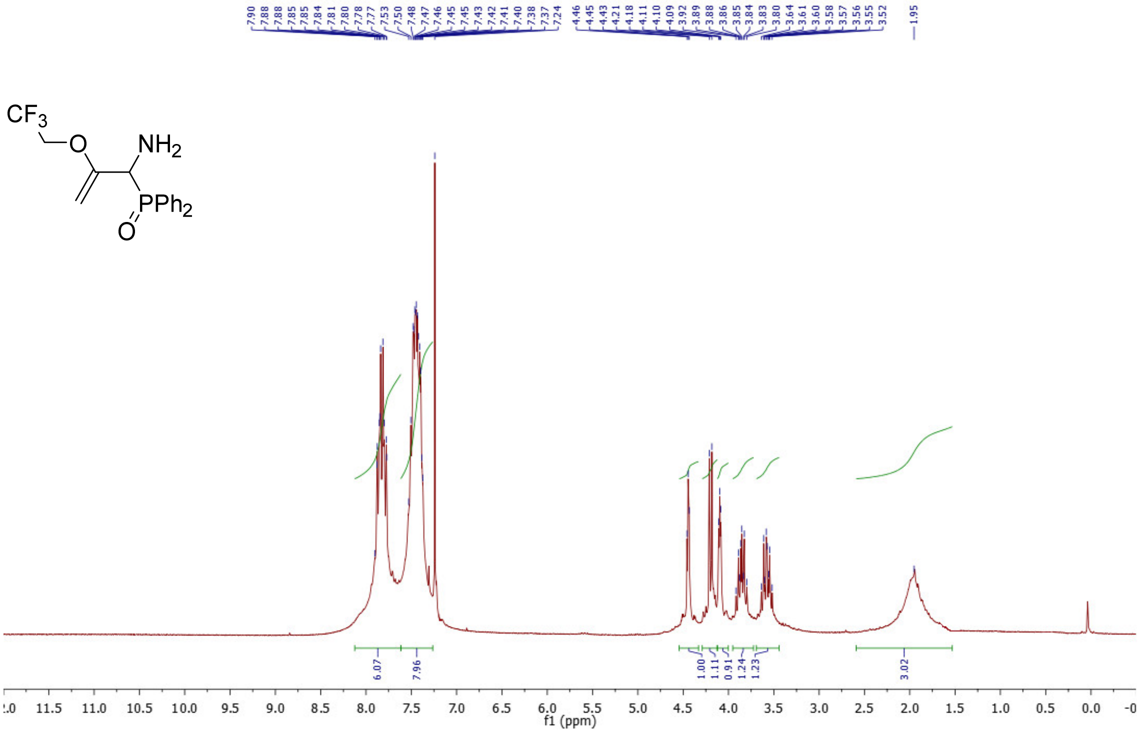

^{13}C {^{1}H} NMR (75 MHz, $CDCl_3$) of compound **8**

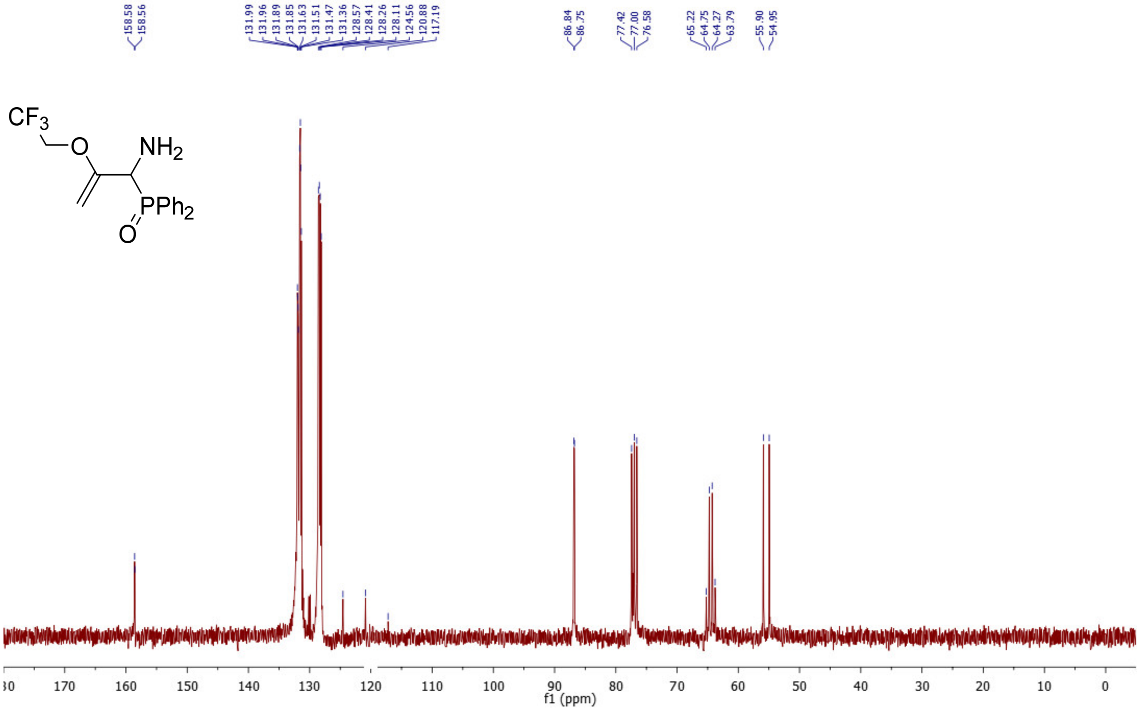

^{1}H NMR (400 Hz, $CDCl_3$) of compound **9**

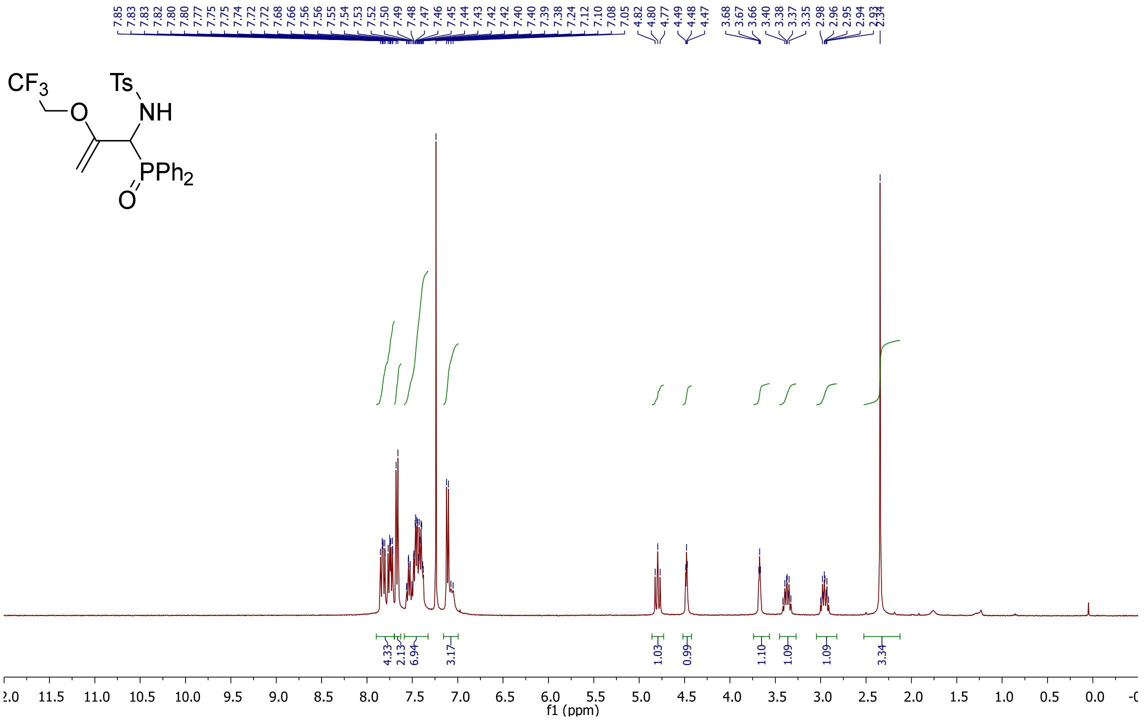

^{13}C {^{1}H} NMR (100 MHz, $CDCl_3$) of compound **9**

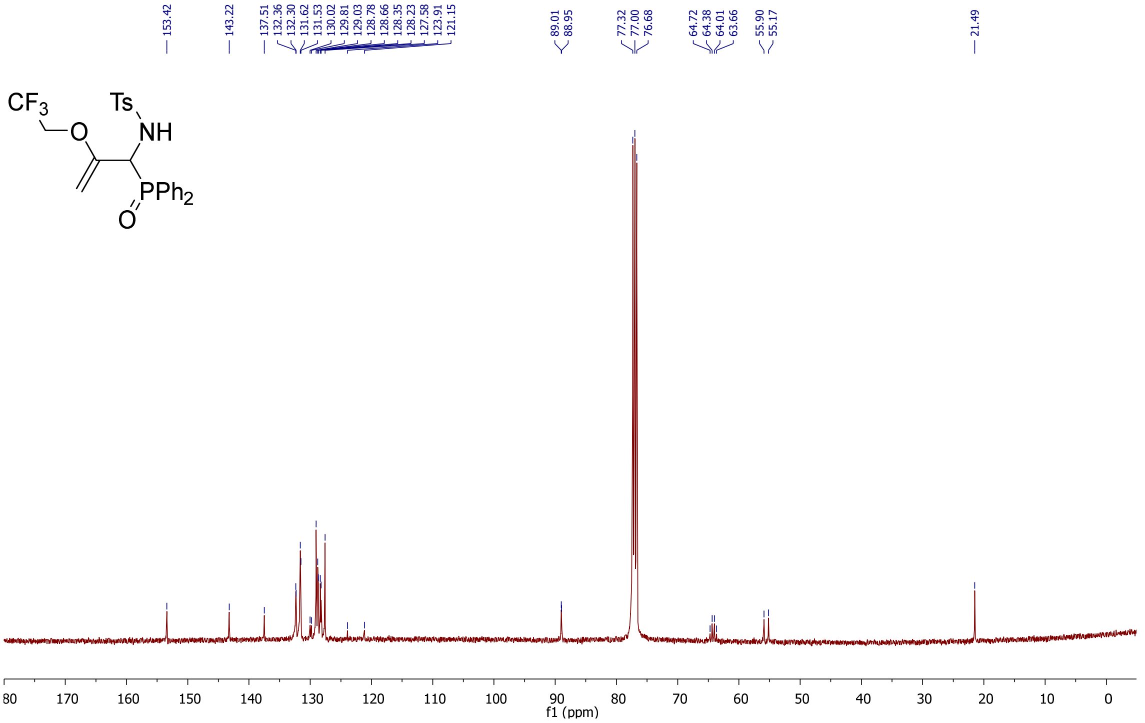

^{1}H NMR (300 Hz, $CDCl_3$) of compound **10a**

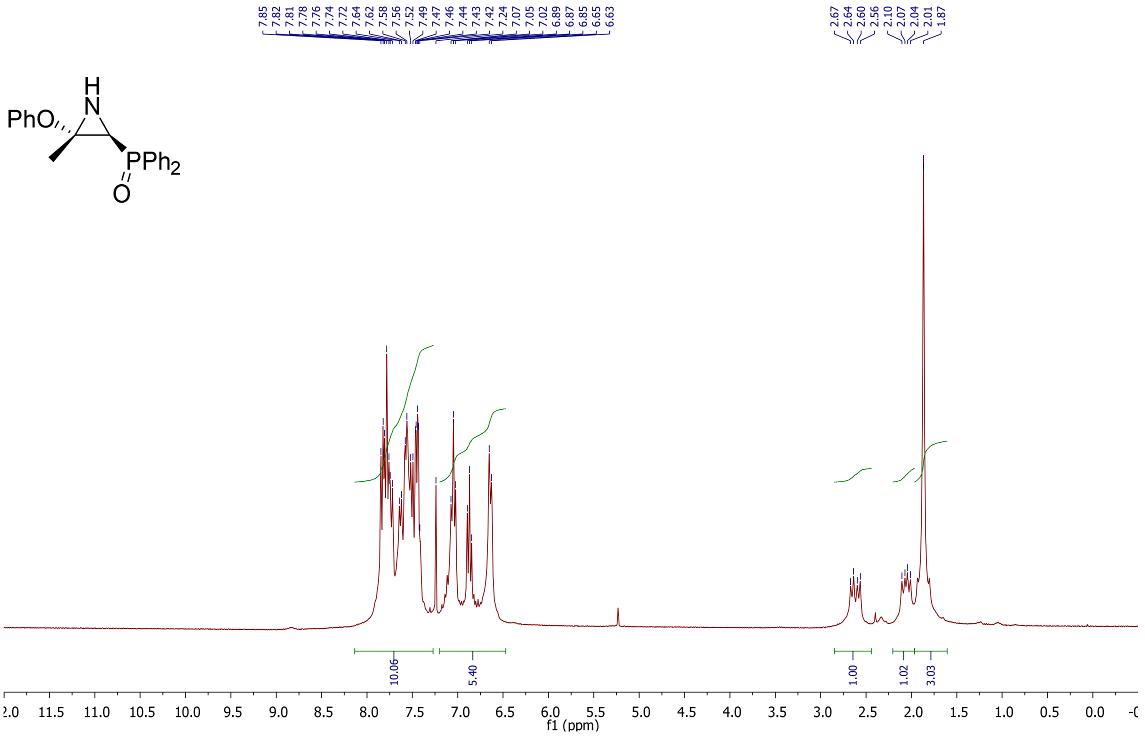

^{13}C {^{1}H} NMR (75 MHz, $CDCl_3$) of compound **10a**

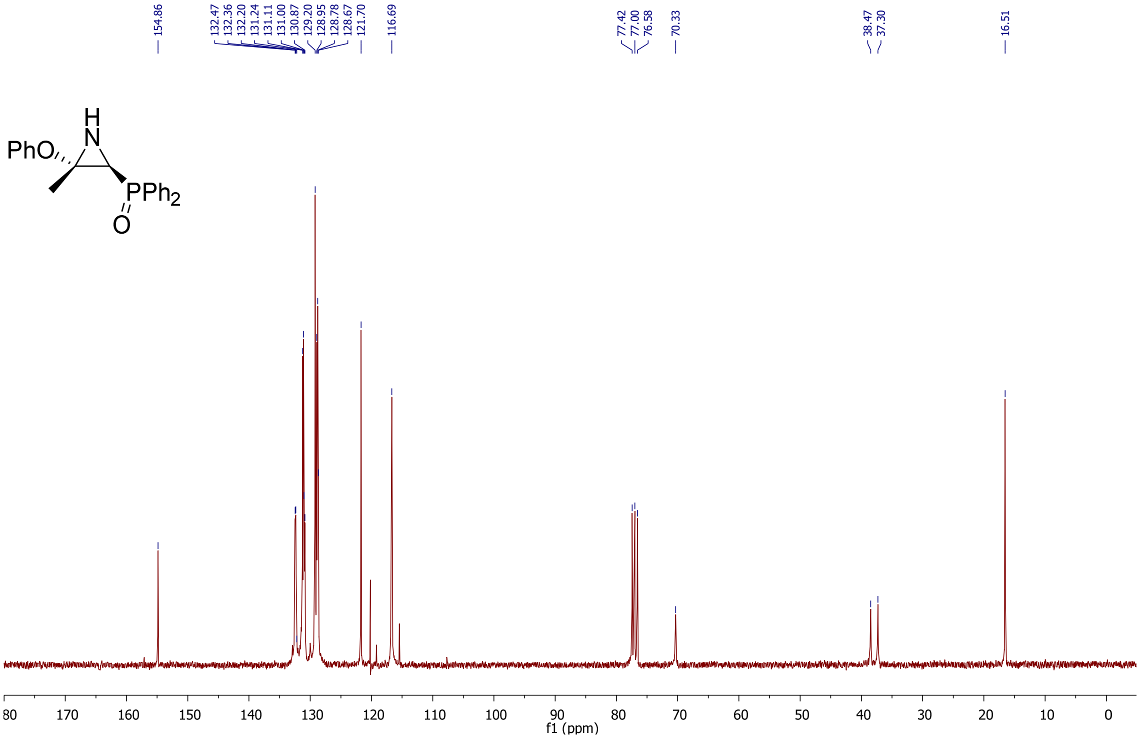

^{1}H NMR (300 Hz, CDCl3) of compound **10b**

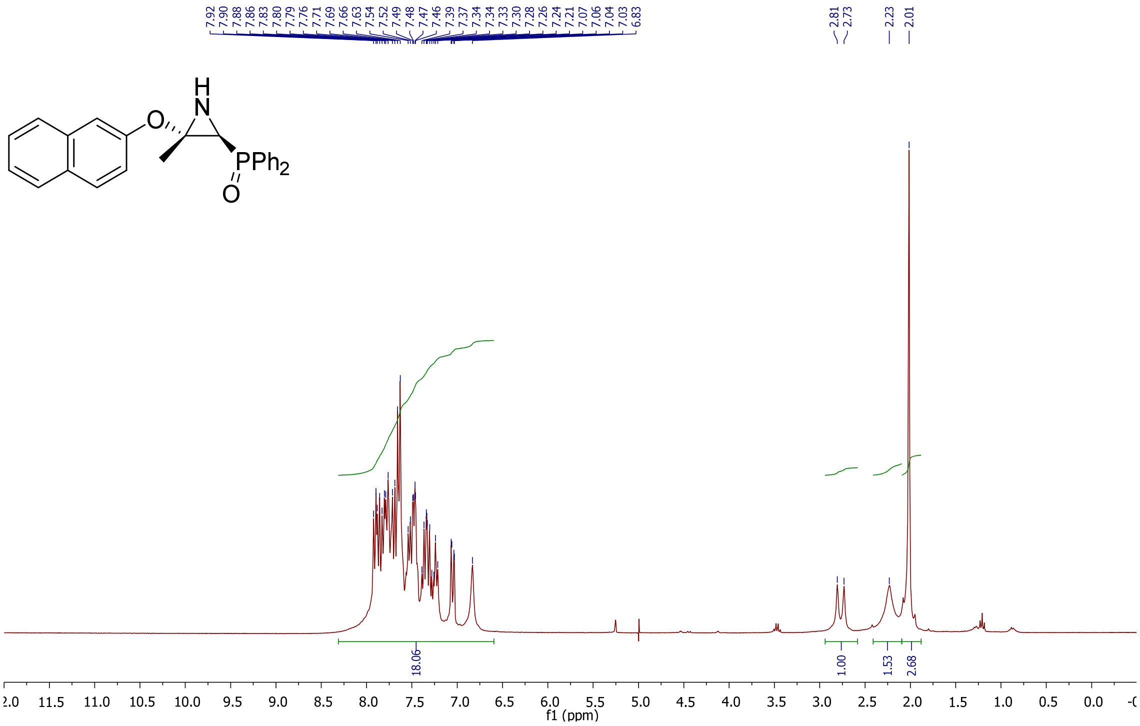

^{1}H NMR (300 Hz, $CDCl_3$) of compound **11a**

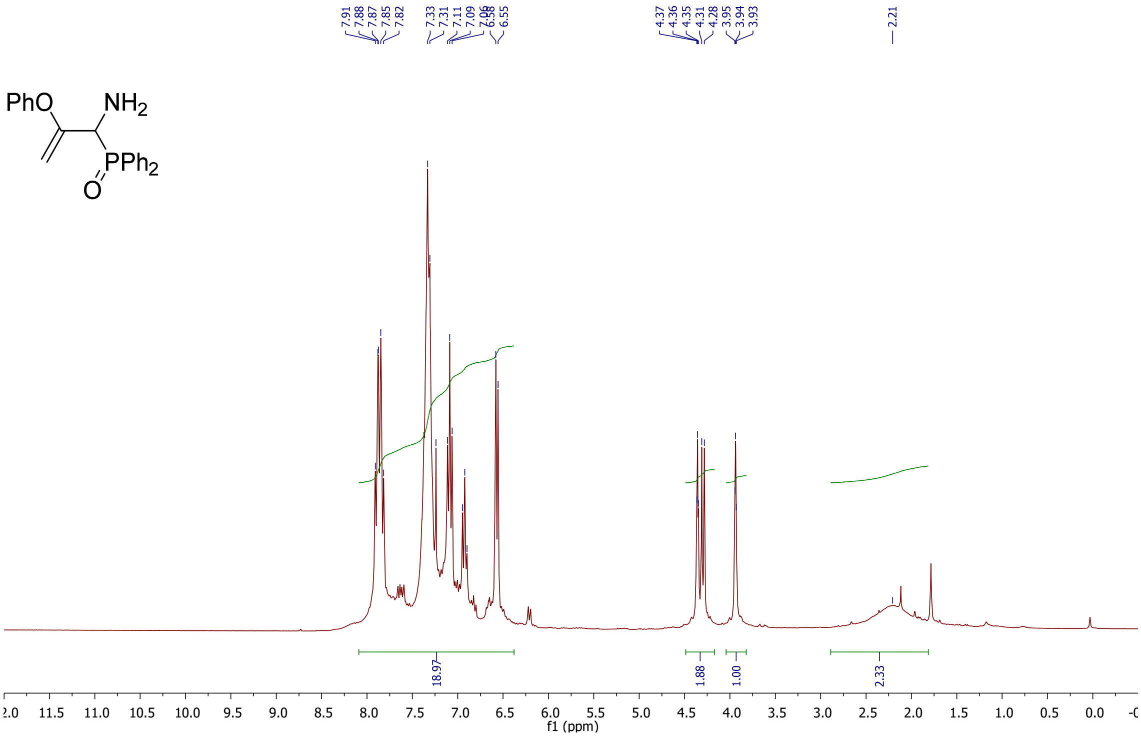

^{13}C {^{1}H} NMR (75 MHz, $CDCl_3$) of compound **11a**

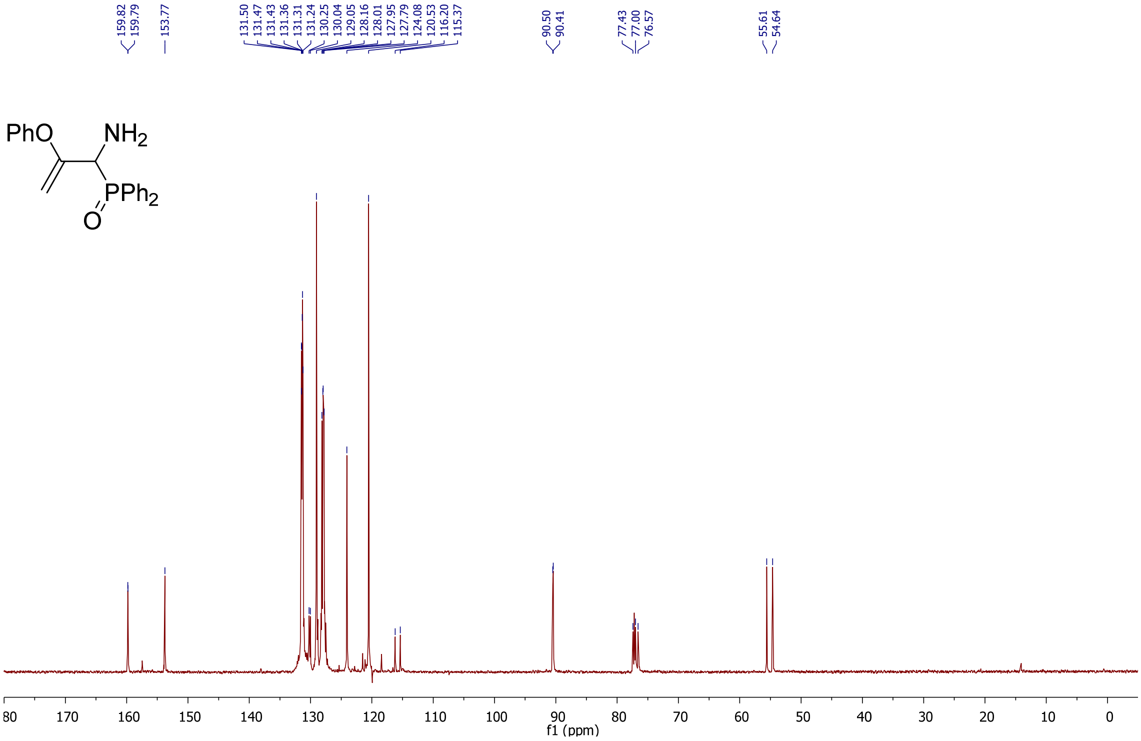

^{1}H NMR (300 Hz, $CDCl_3$) of compound **11b**

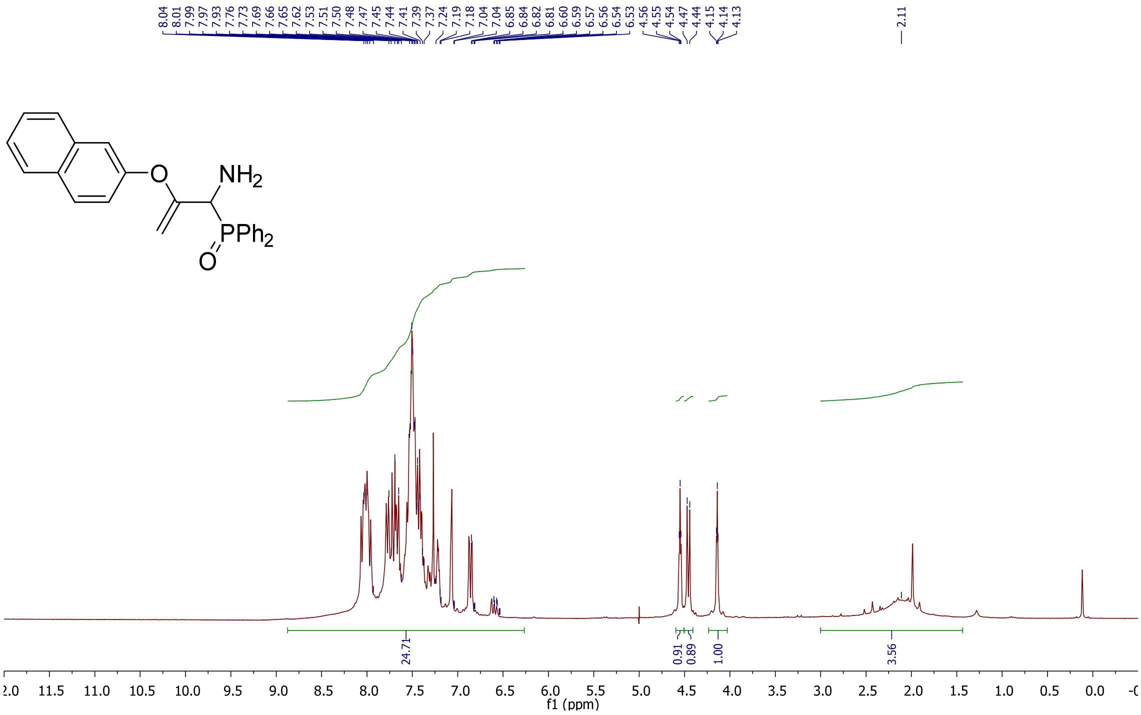

^{13}C {^{1}H} NMR (75 MHz, $CDCl_3$) of compound **11b**

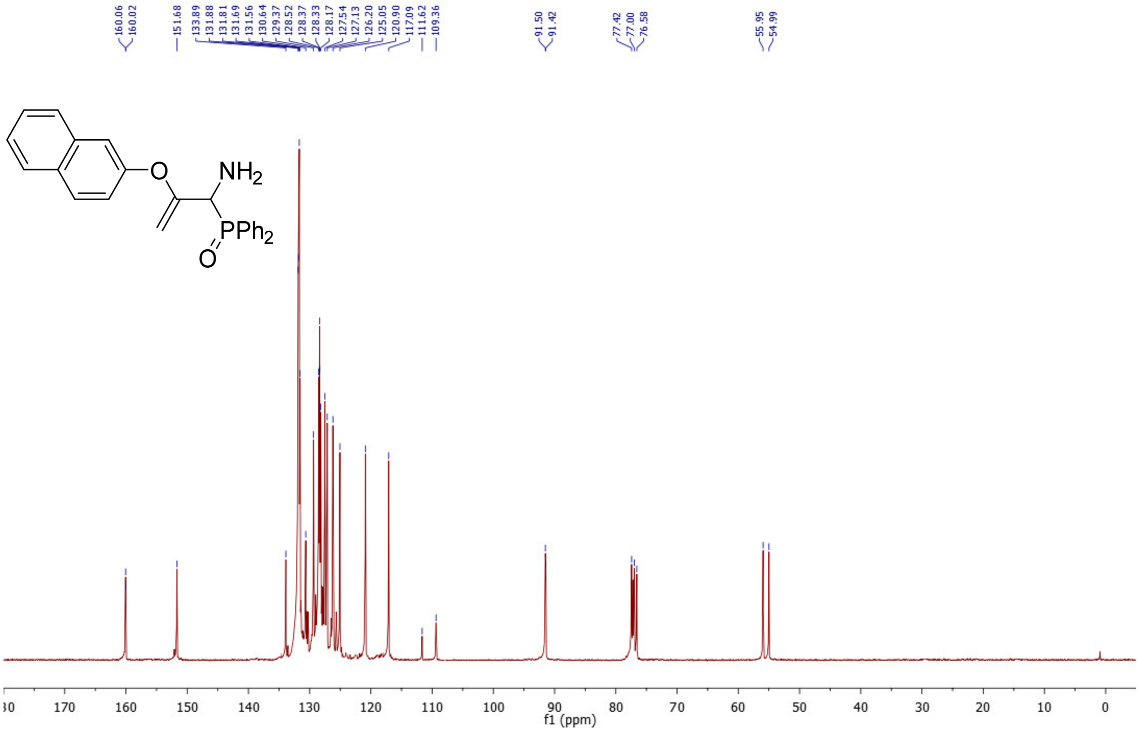

^{1}**H NMR** (400 Hz, $CDCl_3$) of compound **12a**

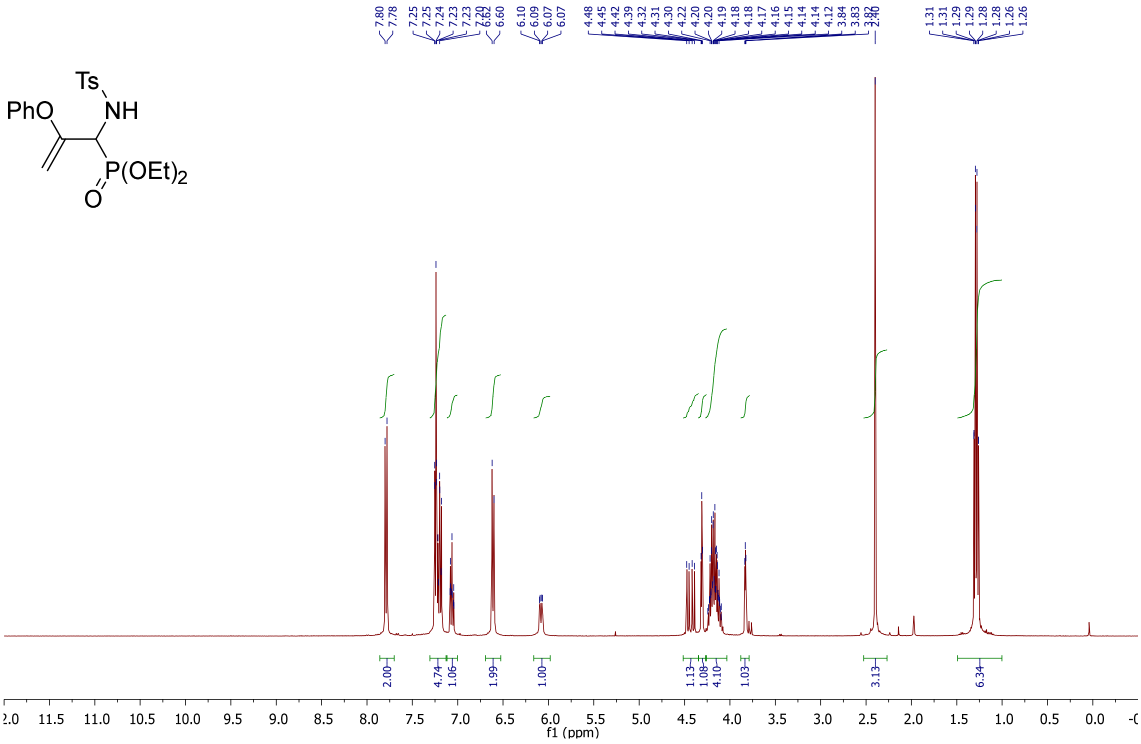

^{13}C {^{1}H} NMR (100 MHz, $CDCl_3$) of compound **12a**

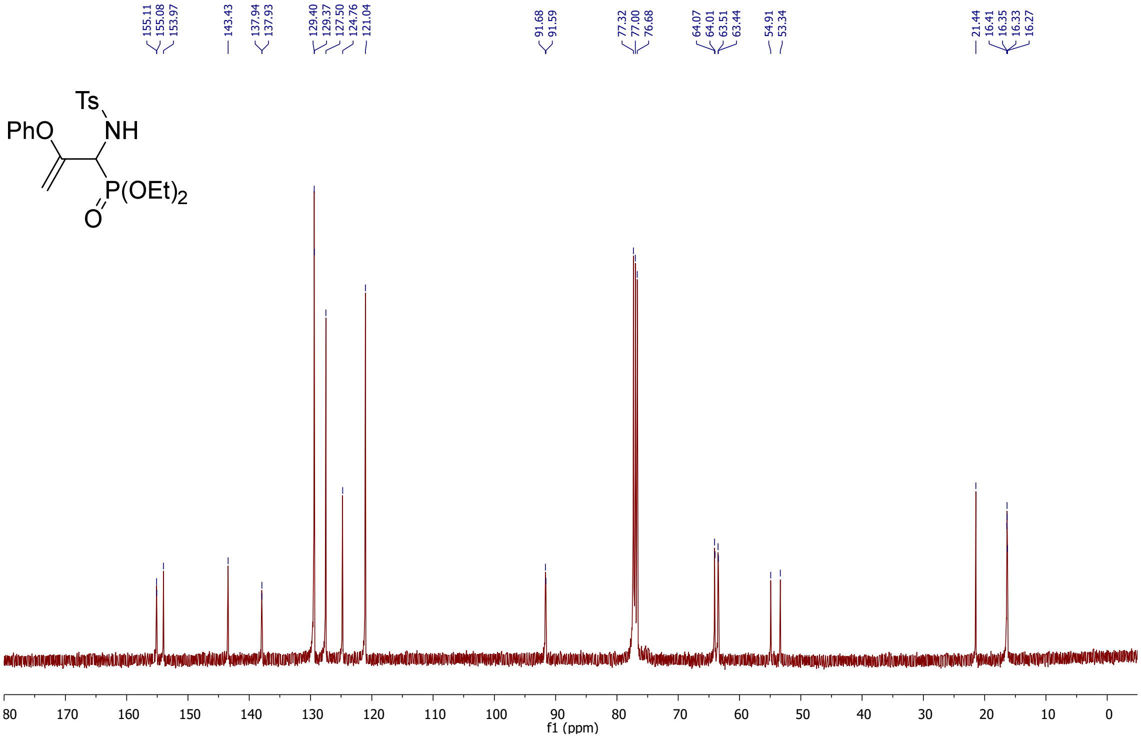

^{1}H NMR (300 Hz, $CDCl_3$) of compound **12b**

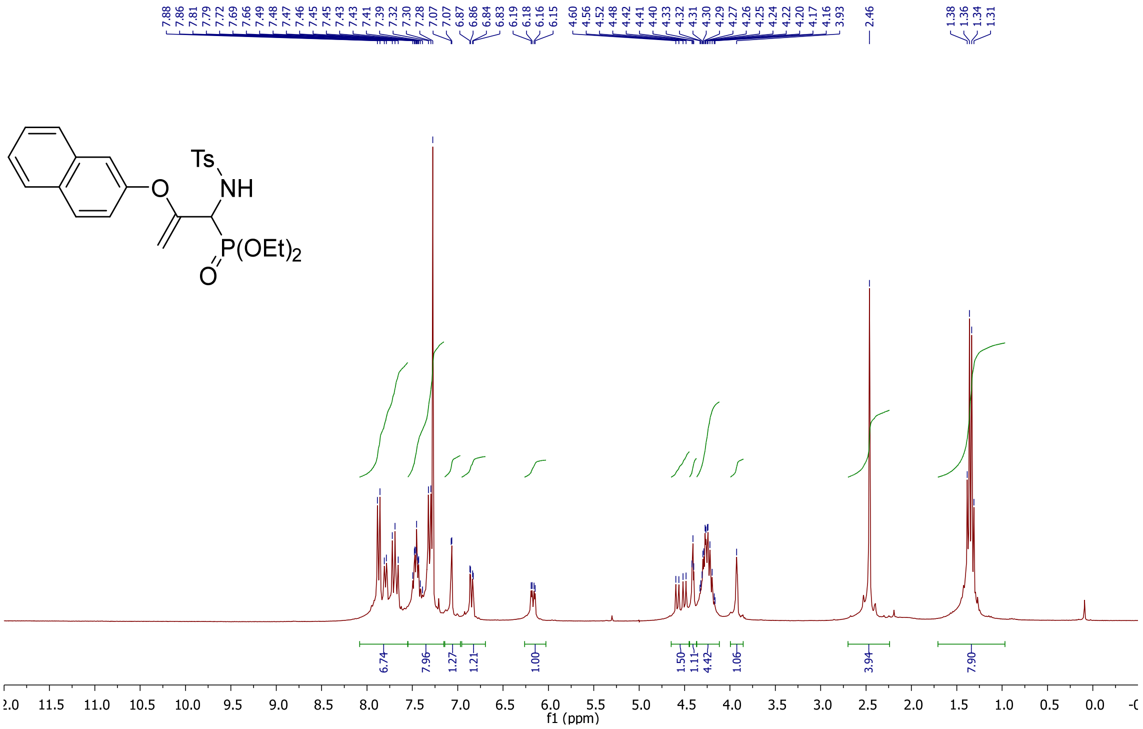

^{13}C {^{1}H} NMR (75 MHz, $CDCl_3$) of compound **12b**

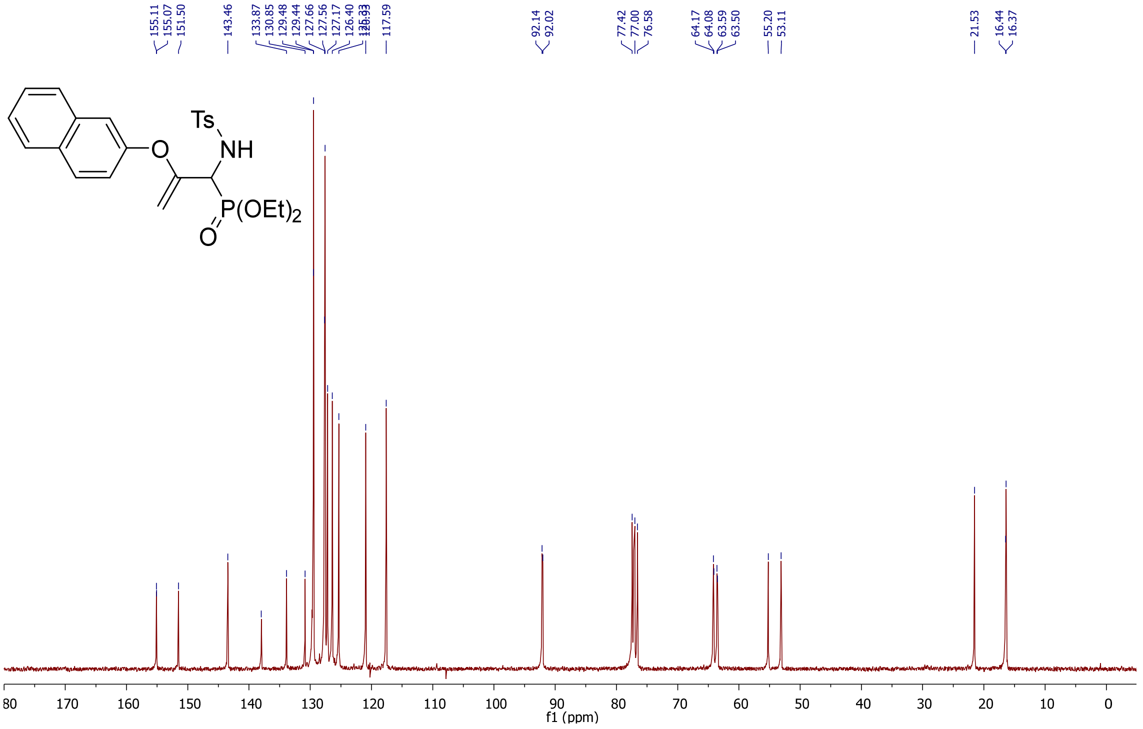

^{1}H NMR (400 Hz, $CDCl_3$) of compound **14a**

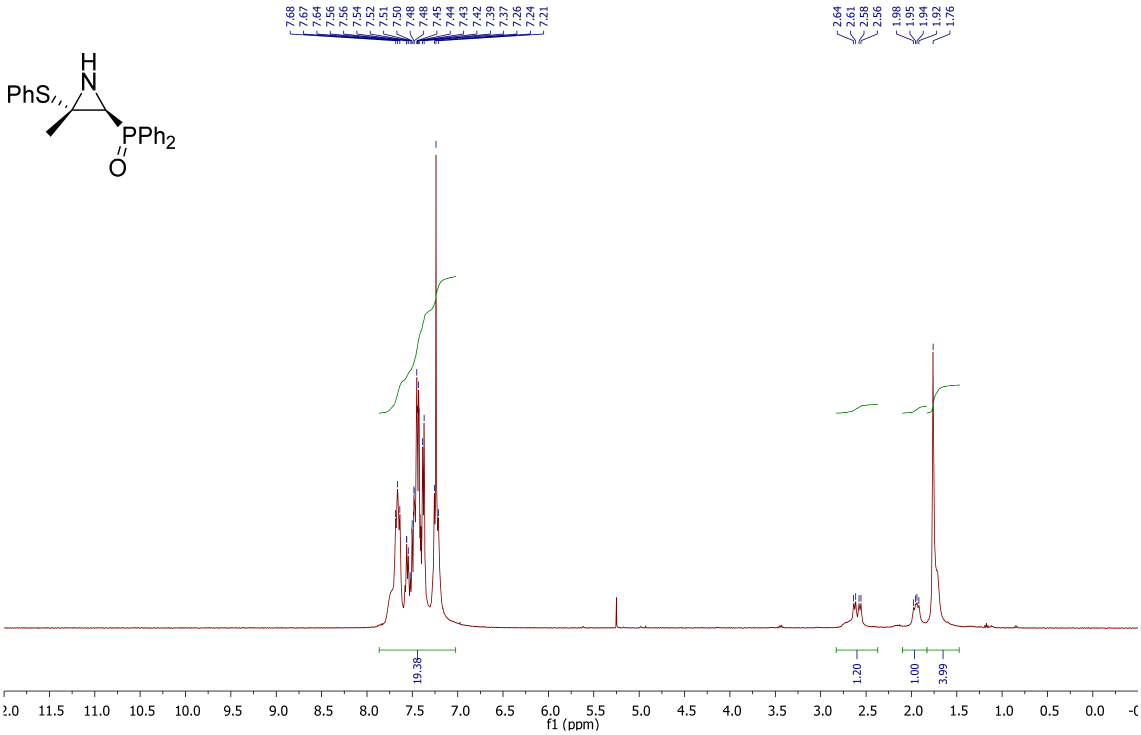

^{1}H NMR (300 Hz, $CDCl_3$) of a crude mixture of **14b** + **15b**.

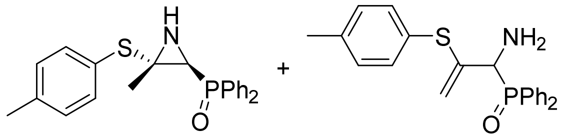

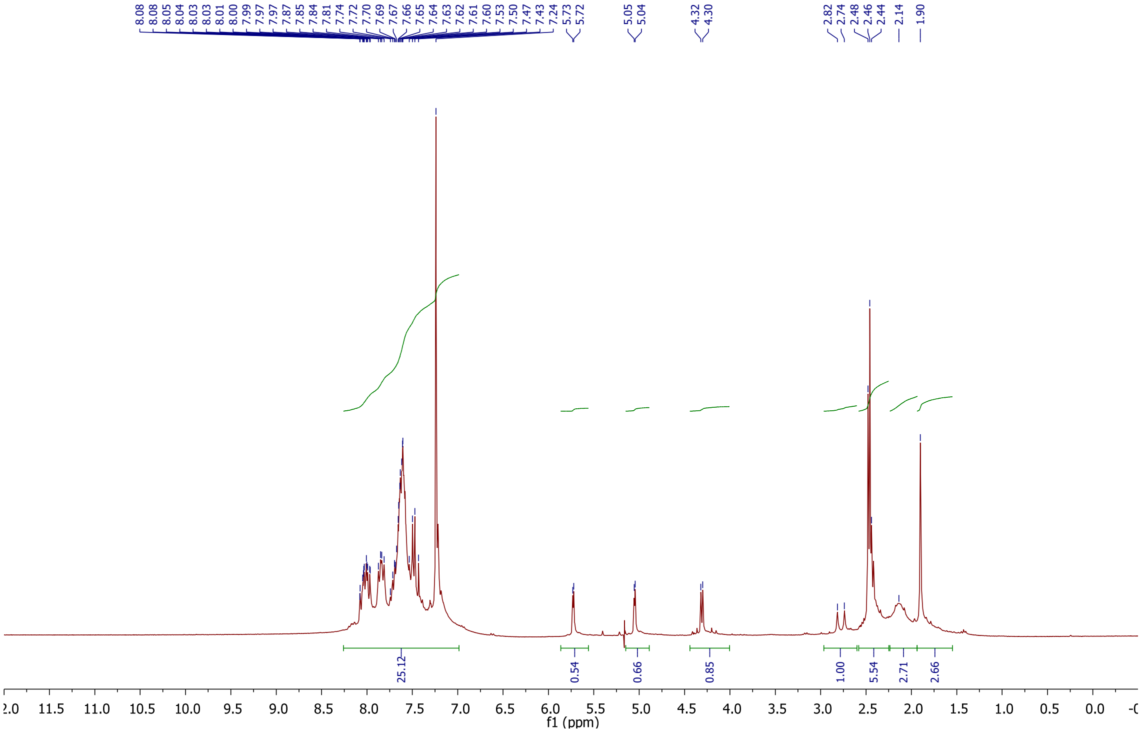

^{1}H NMR (300 Hz, $CDCl_3$) of compound **15b**

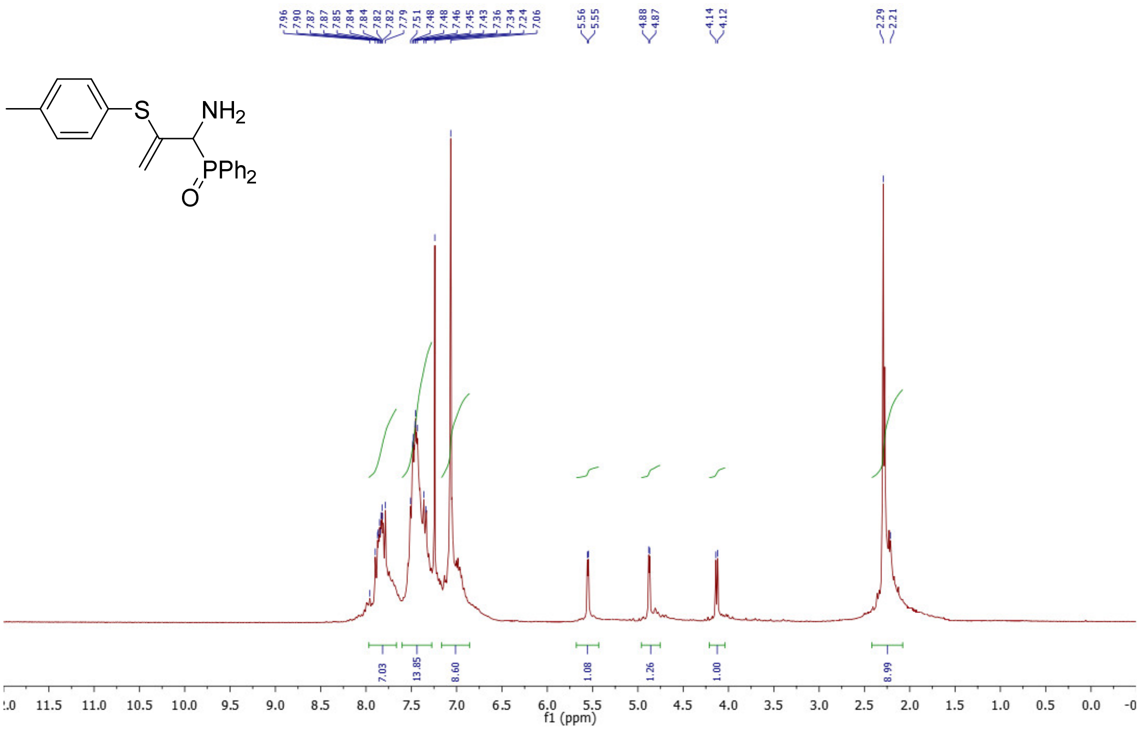

^{13}C {^{1}H} NMR (75 MHz, $CDCl_3$) of compound **15b**

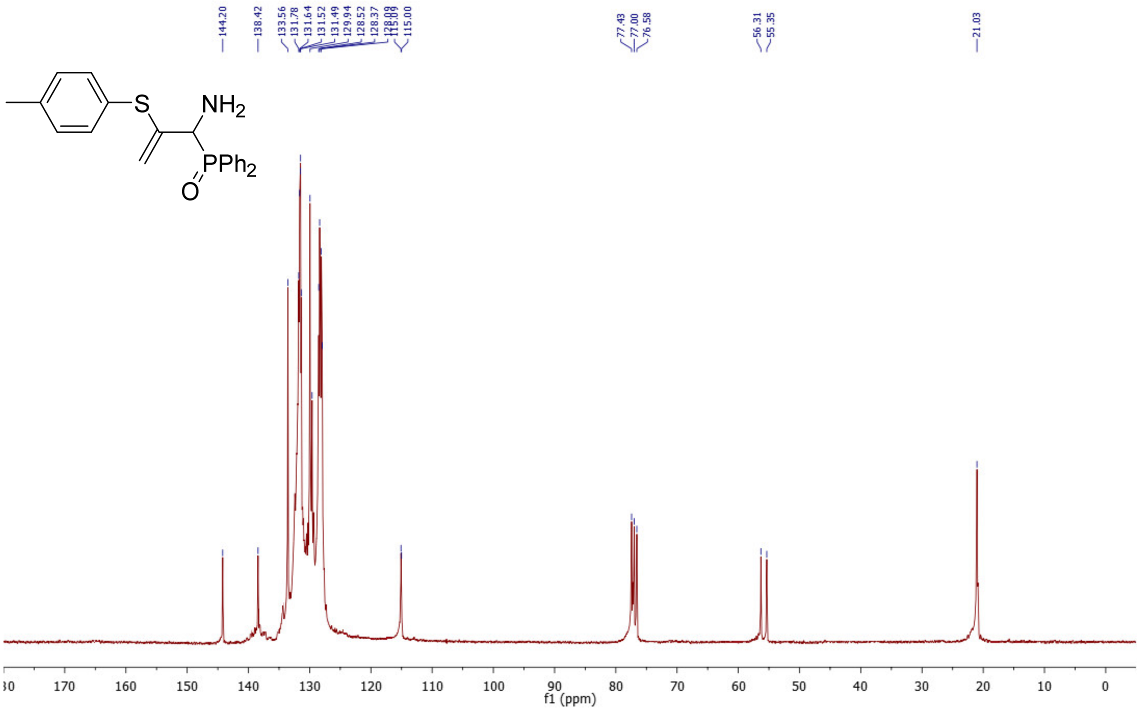

^{1}H NMR (300 Hz, $CDCl_3$) of compound **15c**

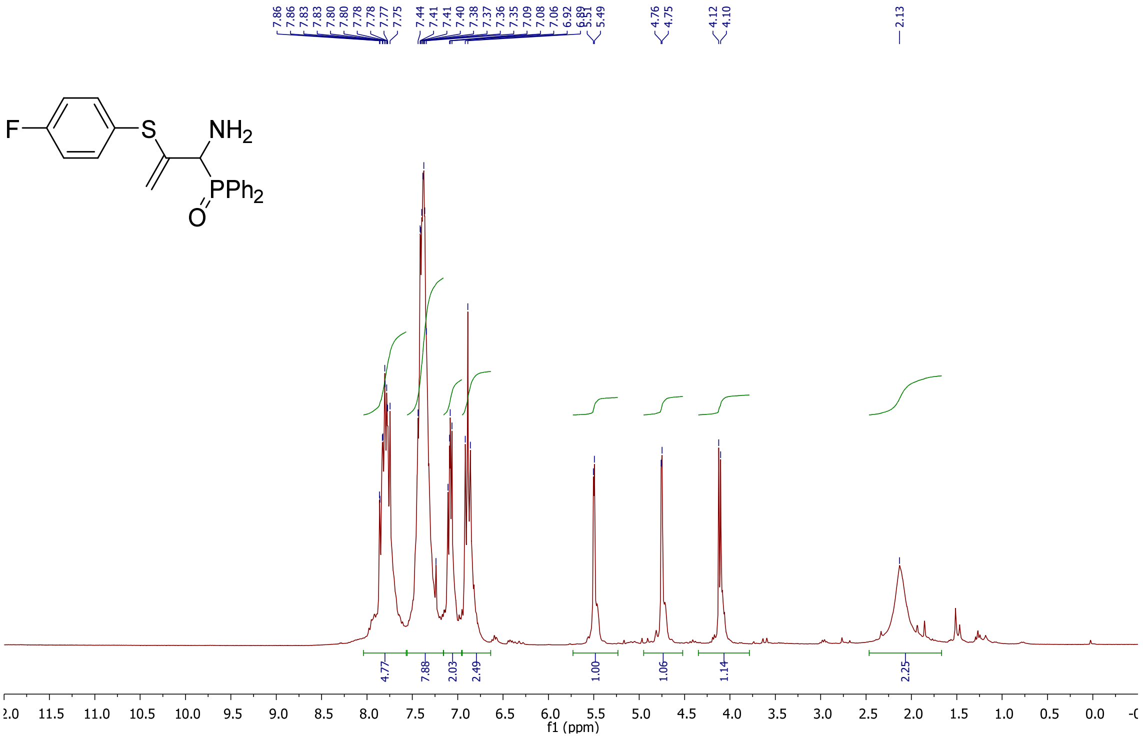

^{13}C {^{1}H} NMR (75 MHz, $CDCl_3$) of compound **15c**

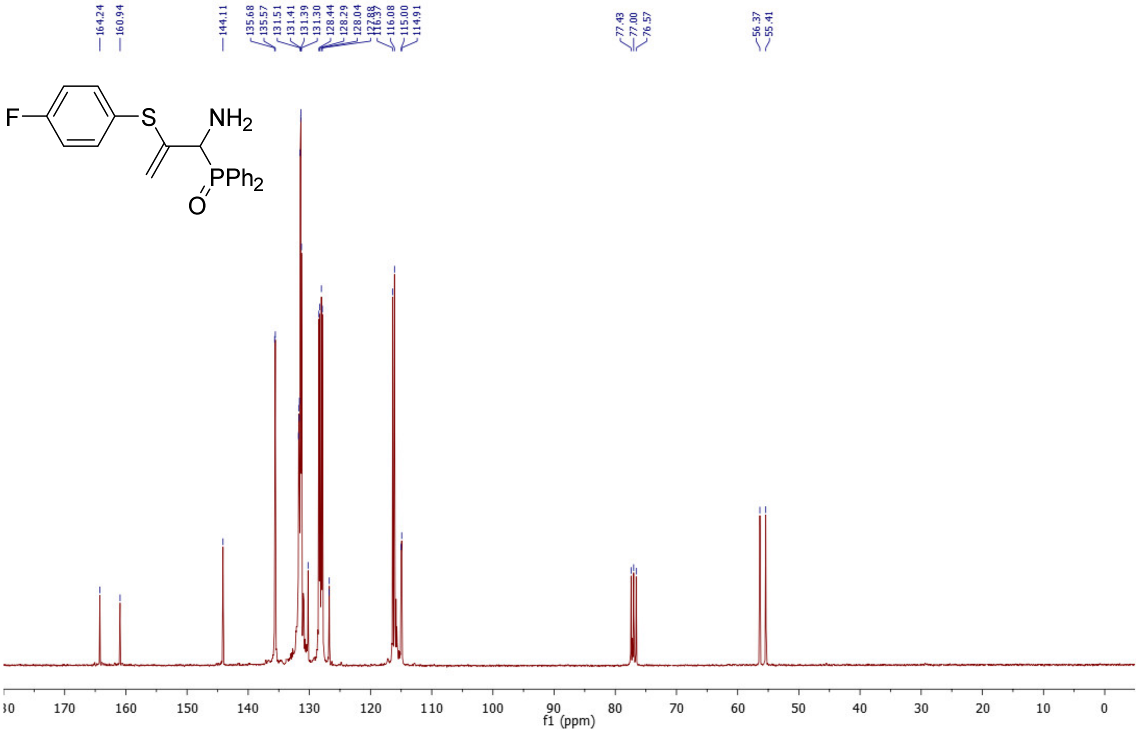

^{1}H NMR (400 Hz, $CDCl_3$) of compound **15d**

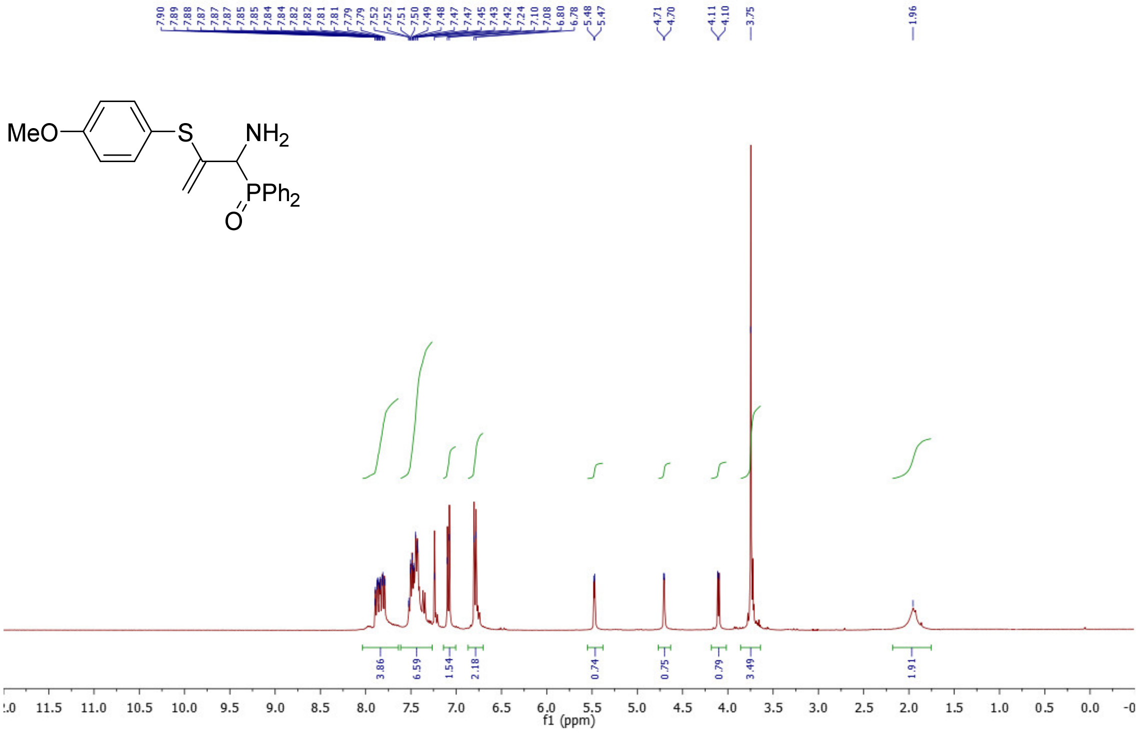

^{13}C {^{1}H} NMR (100 MHz, $CDCl_3$) of compound **15d**

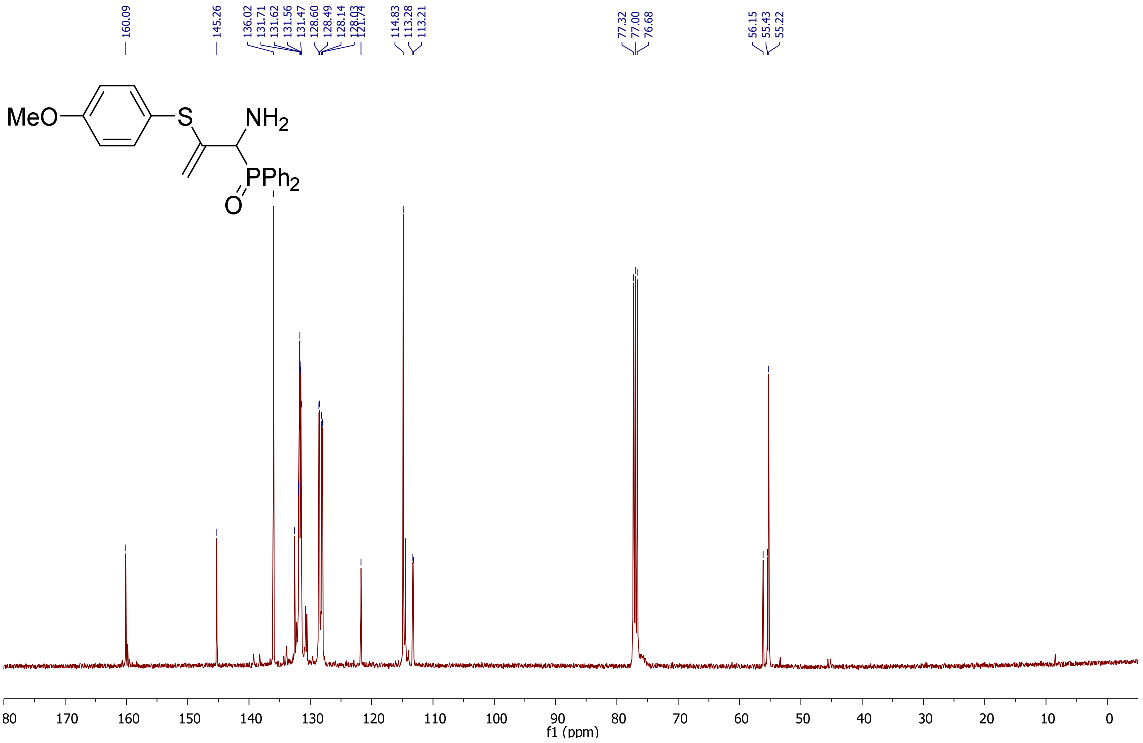

^{1}H NMR (300 Hz, $CDCl_3$) of compound **15f**

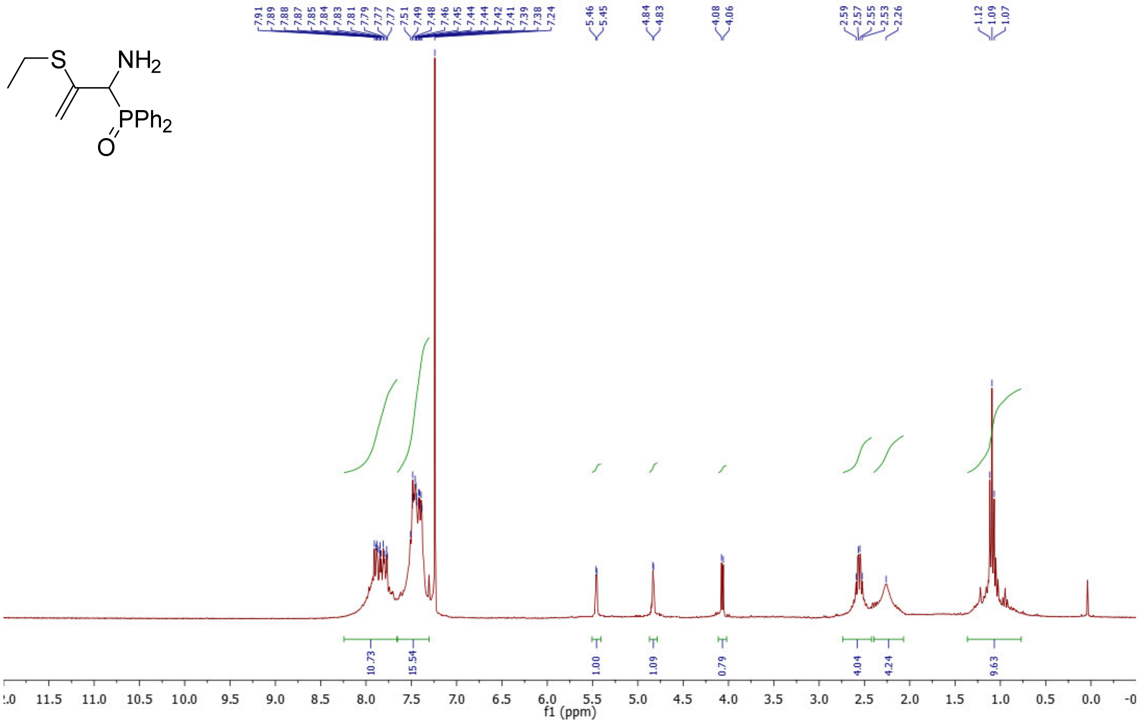

^{13}C {^{1}H} NMR (75 MHz, $CDCl_3$) of compound **15f**

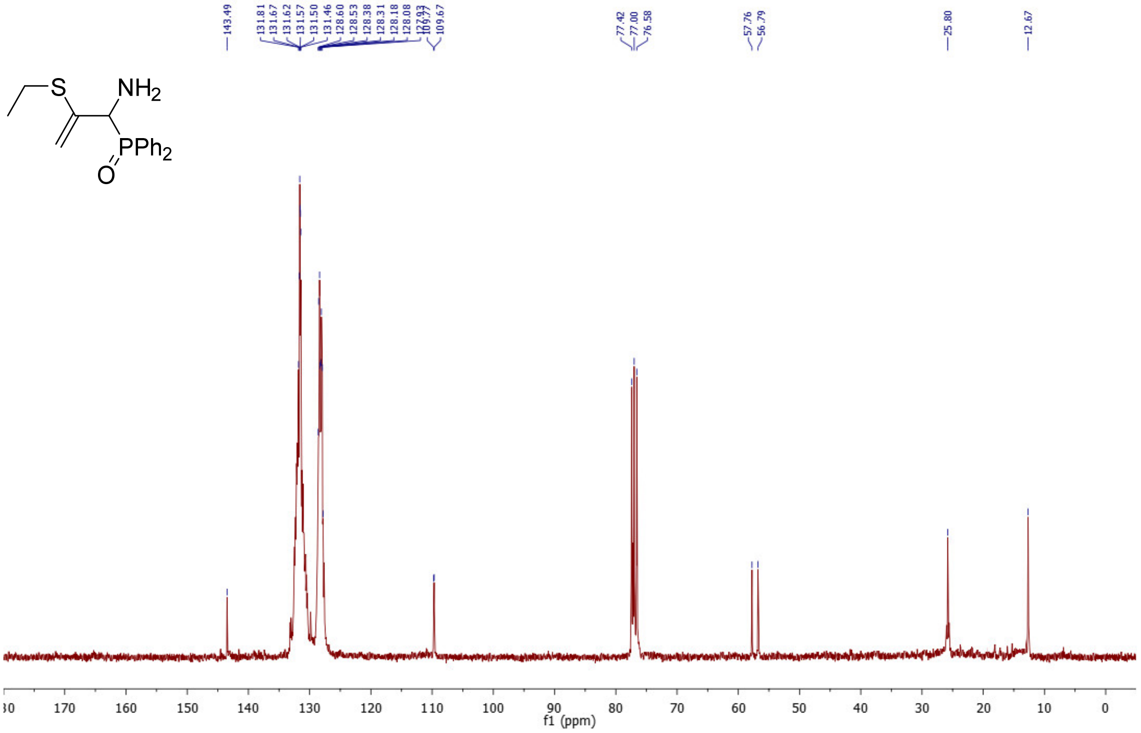

^{1}H NMR (400 Hz, $CDCl_3$) of compound **16a**

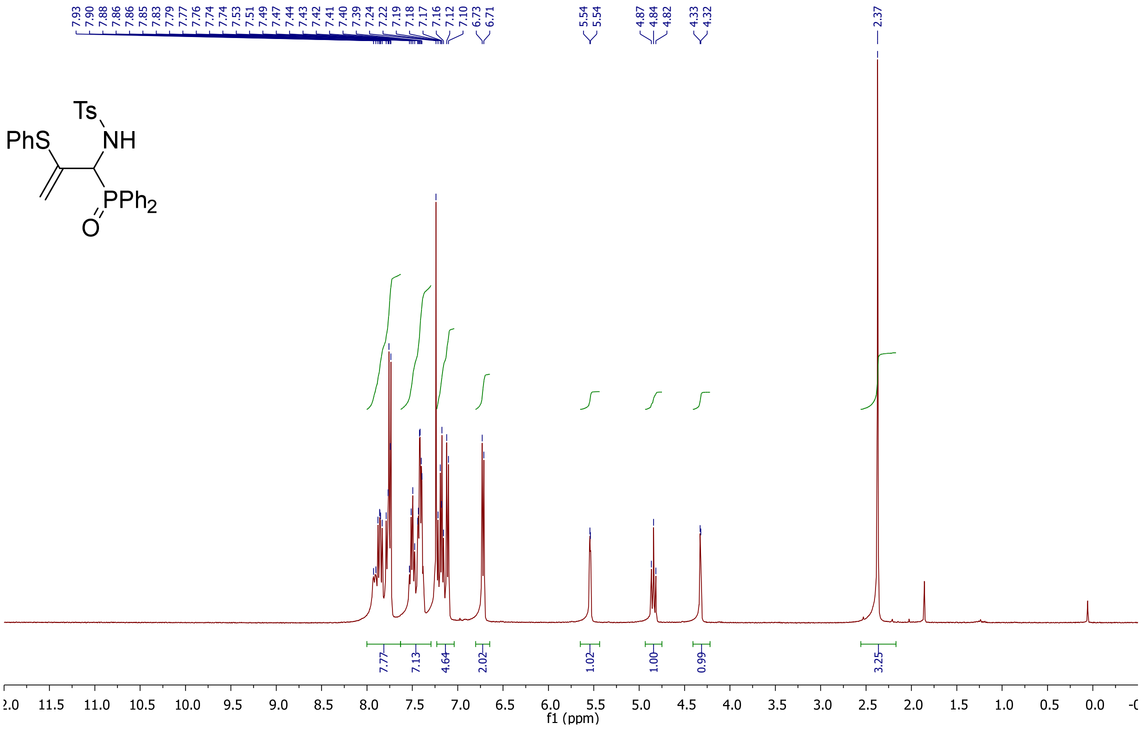

^{13}C {^{1}H} NMR (100 MHz, $CDCl_3$) of compound **16a**

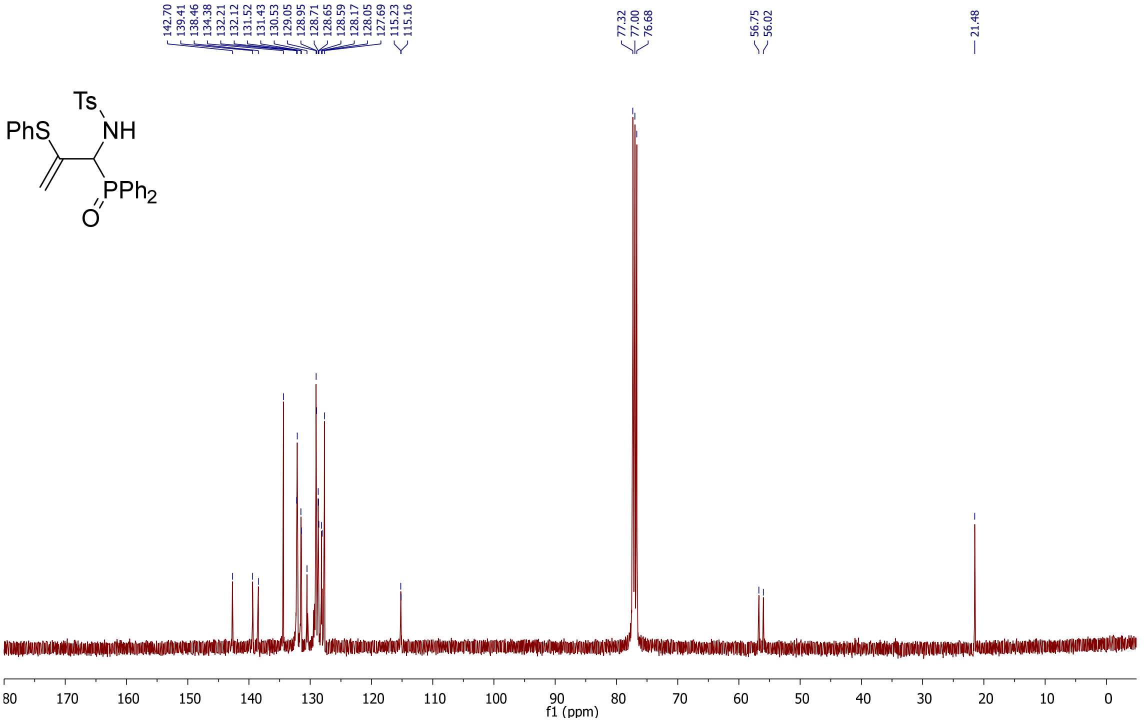

^{1}H NMR (400 Hz, CDCl3) of compound **16b**

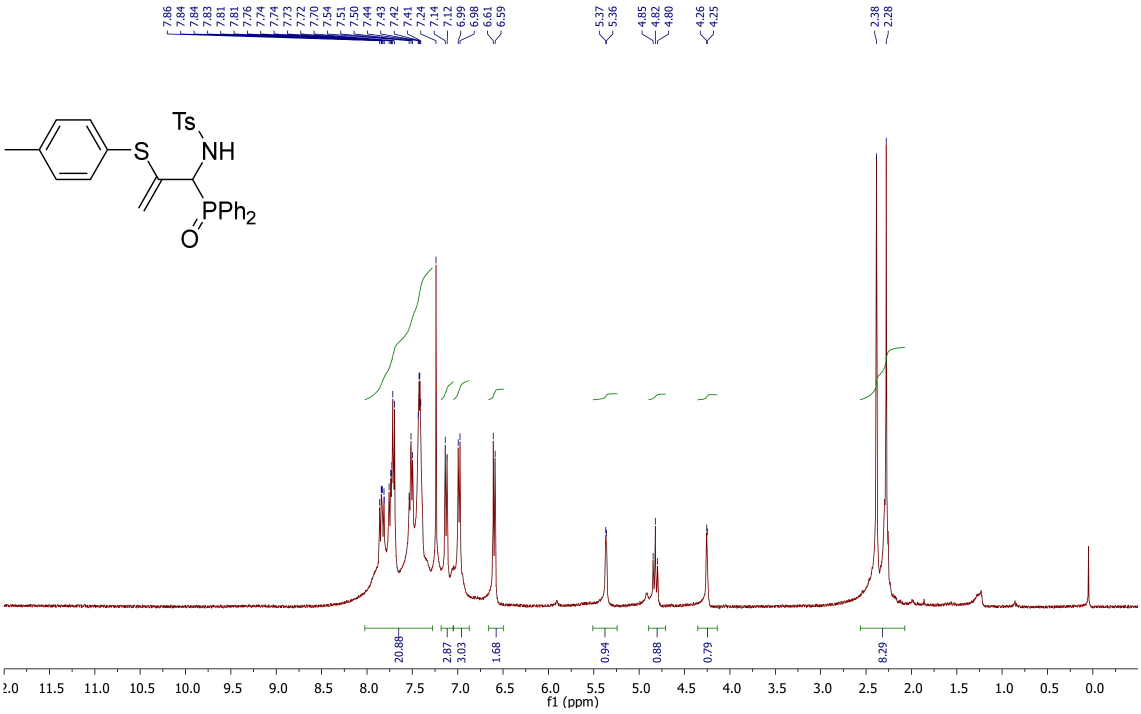

^{13}C {^{1}H} NMR (75 MHz, CDCl3) of compound **16b**

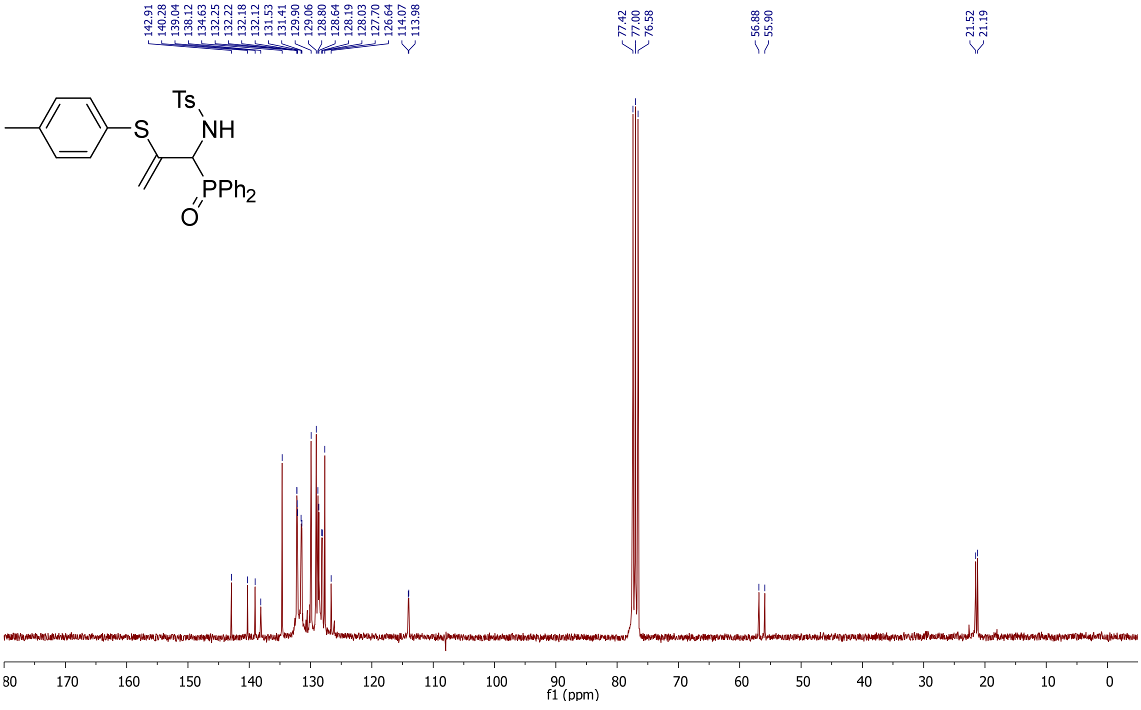

^{1}H NMR (300 Hz, CDCl3) of compound **16c**

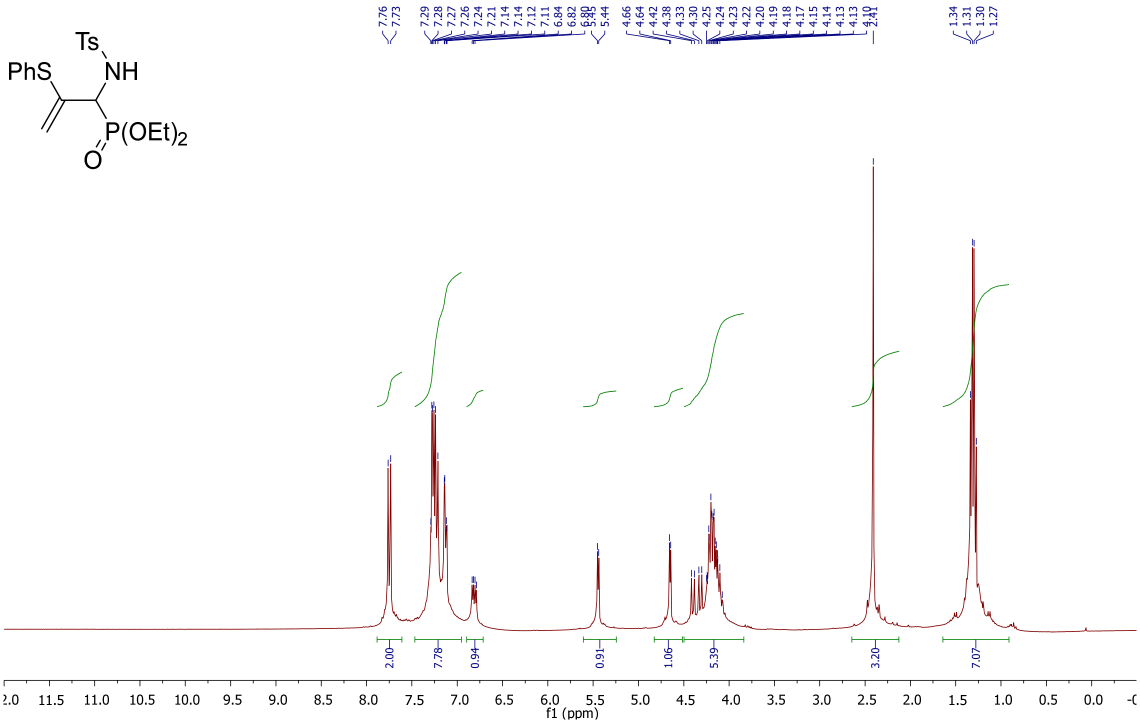

^{13}C {^{1}H} NMR (75 MHz, CDCl3) of compound **16c**

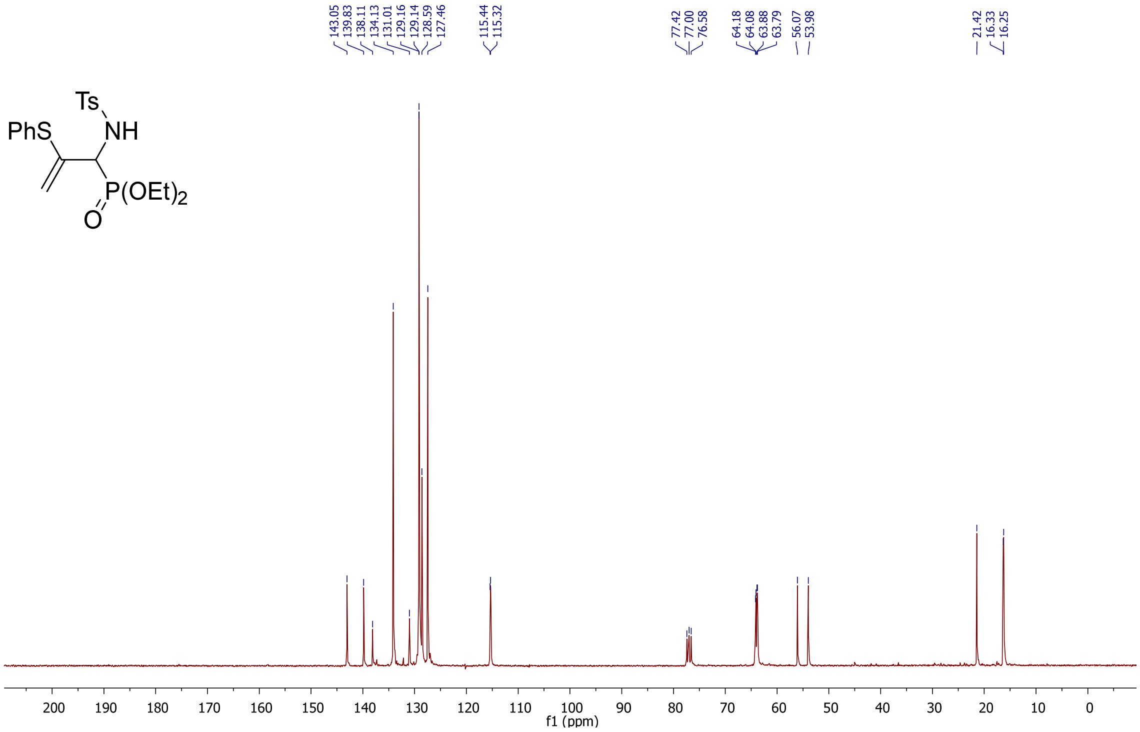

^{1}H NMR (300 Hz, $CDCl_3$) of compound **16d**

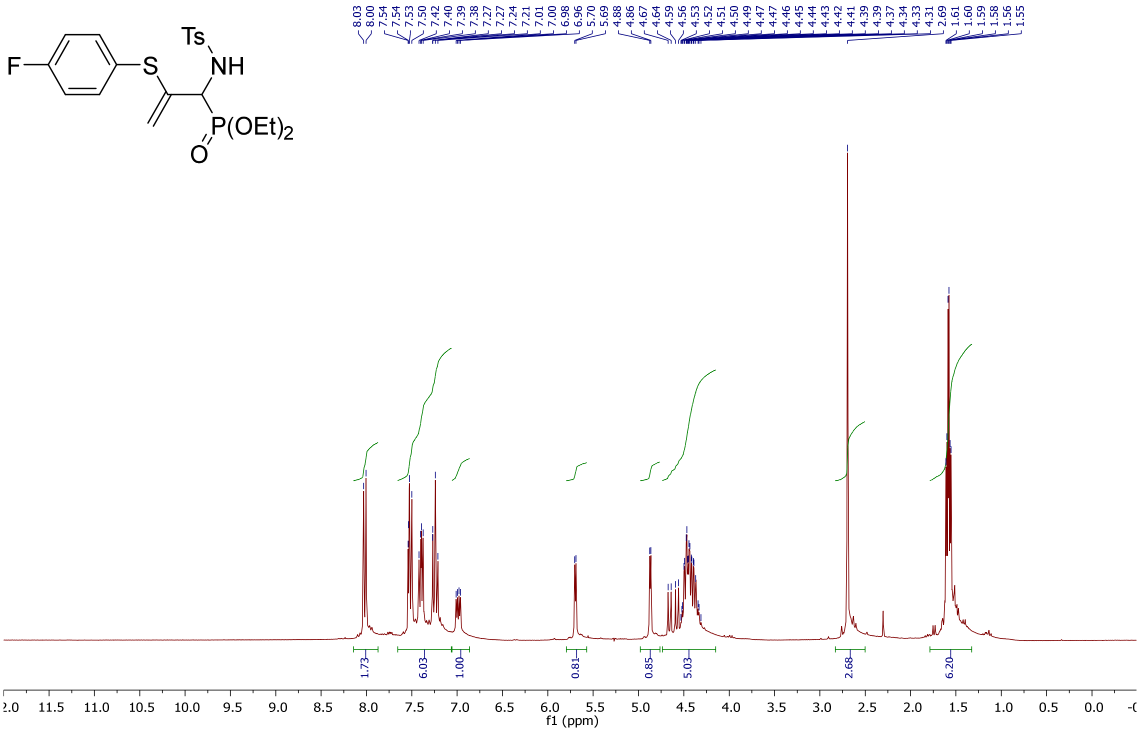

^{13}C {^{1}H} NMR (75 MHz, $CDCl_3$) of compound **16d**

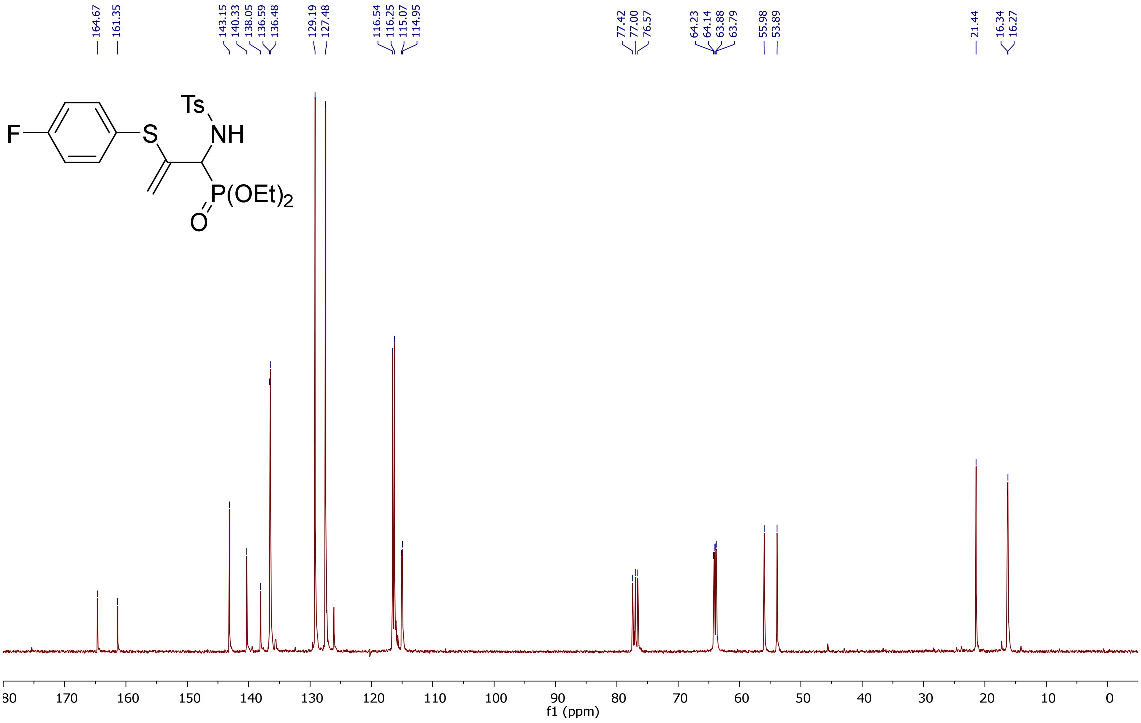

^{1}H NMR (300 Hz, $CDCl_3$) of compound **16e**

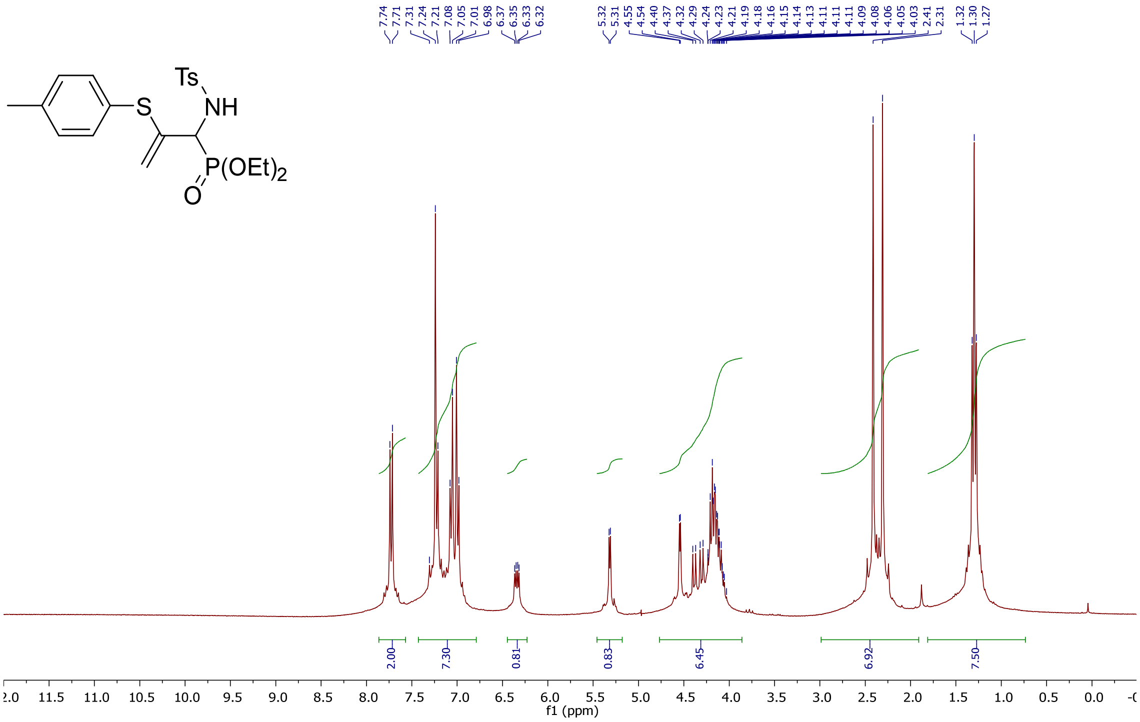

^{13}C {^{1}H} NMR (75 MHz, $CDCl_3$) of compound **16e**

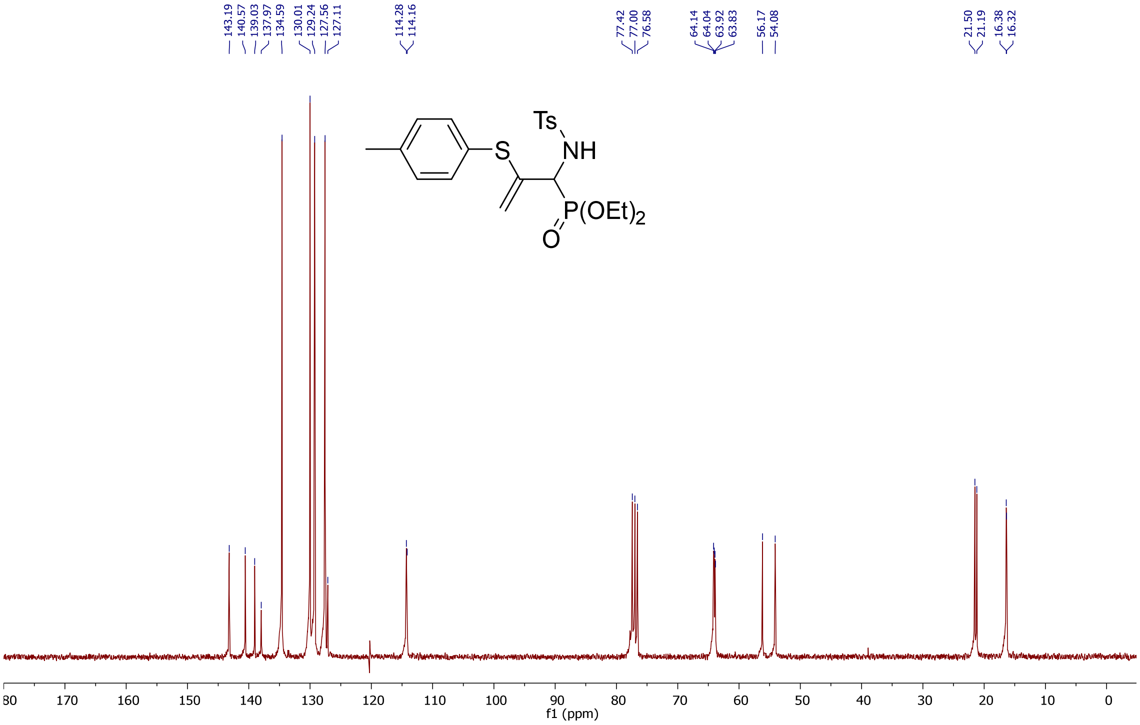

Supplement: Supplementary file 1 [file molecules-25-03332-s001.pdf]
